# Dispirooxindole-β-Lactams: Synthesis via Staudinger Ketene-Imine Cycloaddition and Biological Evaluation

**DOI:** 10.3390/ijms23126666

**Published:** 2022-06-15

**Authors:** Vadim E. Filatov, Dmitrii A. Iuzabchuk, Viktor A. Tafeenko, Yuri K. Grishin, Vitaly A. Roznyatovsky, Dmitrii A. Lukianov, Yulia A. Fedotova, Maxim A. Sukonnikov, Dmitry A. Skvortsov, Nikolai V. Zyk, Elena K. Beloglazkina

**Affiliations:** 1 Department of Chemistry, Lomonosov Moscow State University, Leninskie Gory 1-3, 119234 Moscow, Russia; nanovf@mail.ru (V.E.F.); dmitry.yuzabchuk@gmail.com (D.A.I.); tafeenko-victor@yandex.ru (V.A.T.); ykgris@mail.ru (Y.K.G.); vit.rozn@nmr.chem.msu.ru (V.A.R.); dmitrii.a.lukianov@gmail.com (D.A.L.); yuliya120503@gmail.com (Y.A.F.); sukonnikoff.maxim@yandex.ru (M.A.S.); skvorratd@mail.ru (D.A.S.); zyk@org.chem.msu.ru (N.V.Z.); 2 Center of Life Sciences, Skolkovo Institute of Science and Technology, 143028 Skolkovo, Russia

**Keywords:** spirooxindoles, β-lactams, Staudinger reaction, ketene-imine cycloaddition, oxo-pyrrolidine-3-carboxylic acids

## Abstract

In this work, we present the first synthesis of dispirooxindole-β-lactams employing optimized methodology of *one-pot* Staudinger ketene-imine cycloaddition with N-aryl-2-oxo-pyrrolidine-3-carboxylic acids as the ketene source. Spiroconjugation of indoline-2-one with β-lactams ring is considered to be able to provide stabilization and wide scope of functionalization to resulting scaffolds. The dispipooxindoles obtained demonstrated medium cytotoxicity in the MTT test on A549, MCF7, HEK293, and VA13 cell lines, and one of the compounds demonstrated antibacterial activity against *E. coli* strain LPTD.

## 1. Introduction

While cancer and diseases caused by bacterial infections have always been a substantial social threat, emerging targeted cancer therapies provide means to alleviate it. Strategies for selective targeting and elimination of malignant cells have been developed for the past three decades [1]. Amongst these strategies, an approach based on small-molecule agents has attracted major interest [2]. Some low molecular weight scaffolds may be the basis for the development of new antibacterial and anticancer drugs; these small-molecule therapeutic agents offer several significant advantages such as high stability in biological environments, allowing for oral administration and high potential of modification and functionalization [2,3]. The potential for modification and the functionalization of small-molecule therapeutic agents is especially important for reducing the toxicity toward healthy human cells, which is significant not only for antibacterial therapy, but improves efficiency in suppressing tumor cells [2,3].

Among antibiotics, one of the most well-known scaffolds is β-lactam. The main problem of these antibiotics is the common resistance the bacteria to this antibiotic. This problem may be overcome by combining antibiotics with beta-lactamase inhibitors or new antibiotics that are resistant to degradation [4]. The latter approach requires the development of new molecular scaffolds. The compounds considered below have a rigid three-dimensional framework while containing a β-lactam ring, and it would be interesting to consider whether it has antibacterial activity. Moreover, β-lactam-based compounds were described recently as anticancer prodrugs and enzyme inhibitors [5].

**Figure 1:**
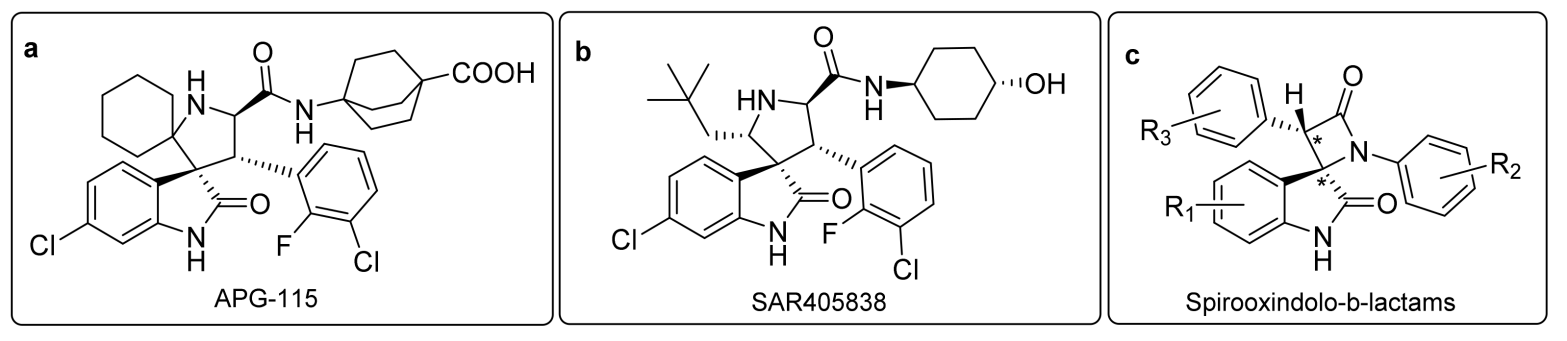
Spirooxindole-based small-molecule inhibitors of MDM2. Spirooxindole-pyrrolidines: (**a**) APG-115, (**b**) SAR405838; (**c**) bis-aryl spirooxindole-β-lactams.

Diversification of molecular targets has been recently discovered, resulting in the development of multitude of small-molecule targeted therapeutics [6]. Although different ways to inhibit tumor cell proliferation are known, the targeting apoptosis is considered to be the most effective since it potentially leads to a complete elimination of malignant tissues [7,8]. Thus, as a result, strategies based on up-regulation of apoptosis-inducing p53 protein (also known as “genome guardian”) through inhibition of its antagonist, cellular protein MDM2, has become a foundation for the design and synthesis of small-molecule agents for targeted therapy [9,10,11,12,13,14]. Several examples of different inhibitors belonging to different structure types are known to exhibit impressive antitumor activity [15,16,17,18]. Among these molecules, inhibitors based on spirooxindole scaffold attract major interest [19,20,21]. An oxindole ring is bioisosteric to indole moiety of tryptophan, interaction with which is fundamental for binding to MDM2, while spiro-conjugation provides synthesized molecules structural rigidity and wide scope of functionalization [21]. Several spirooxindole-based drugs for targeted therapy such as APG-115 (Figure 1a) and SAR405838 (Figure 1b) exhibited great tumor-suppressing activity in recent clinic trials [22,23,24,25].

Despite design and development being primarily focused on spirooxindoles conjugated with pyrrolidine ring, other structuraltypes of spiroindoline-2-ones are known to exhibit antiproliferative activity [26]. Recently, we have reported convenient methodologies for the synthesis of novel bis-aryl spiro[azetidine-2,3′-indoline]-2′,4-diones (Figure 1c) displaying good affinity with targets at docking studies and preliminary in vitro tests [27].

The development of the latter compounds, in which the oxindole moiety is spiro-conjugated with the β-lactam ring, are based on the idea of β-lactams ring stability in the cell environment, as proven by work of O’Boyle and coworkers on β-lactams combretastatin-A4 analogues [28,29]. Note that, although the introduction of additional spiro-conjugation in synthesized compounds to enhance rigidity and functionalization of resulting molecules is common for spirooxindole-pyrrolidines, it is hitherto unknown for spirooxindole-β-lactams. In this article, we report the design and synthesis of a novel dispirooxindole-β-lactams scaffold.

## 2. Results and Discussion

### 2.1. Synthesis of Dispirooxindole-β-Lactams

**Figure 2:**
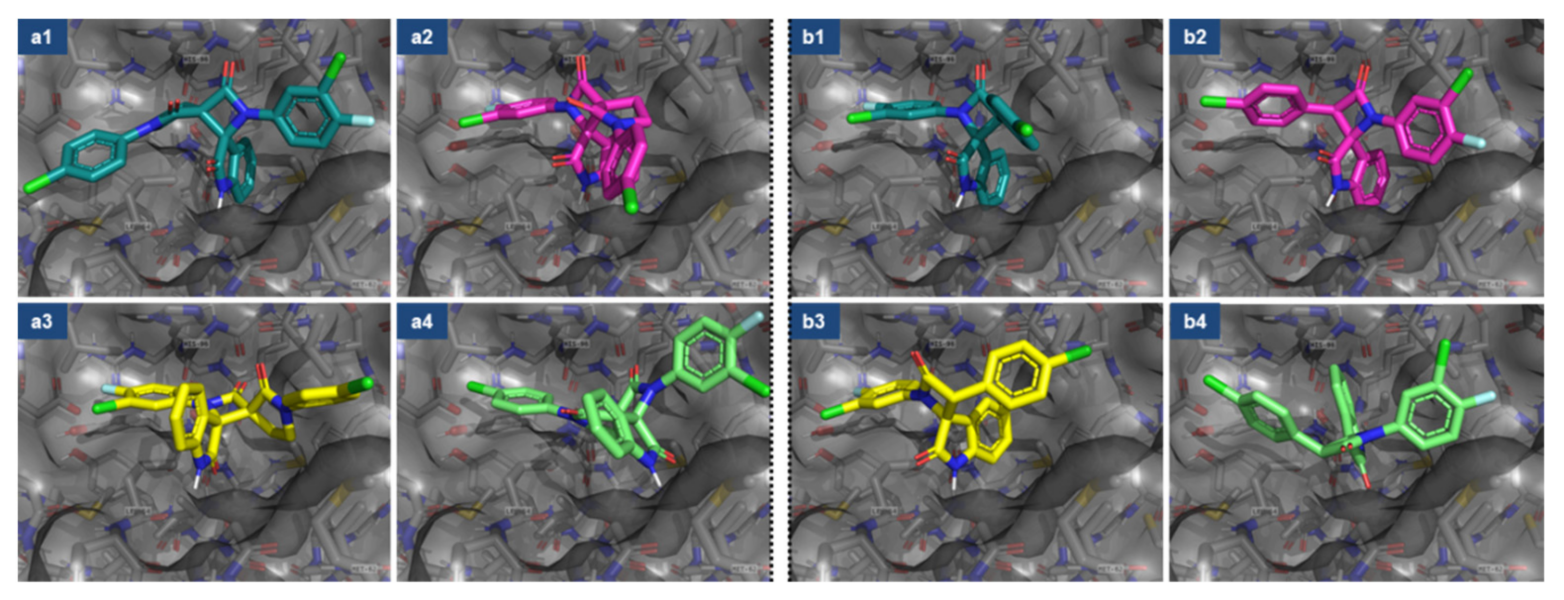
Molecular docking studies of dispiro- (**a**) and spiro- (**b**) indolin-2-ones (see Table 1). Binding modes of four stereoisomers of dispirooxindole-β-lactam (**a1**–**a4**) are compared to isomers of previously studied spirooxindole-β-lactam (**b1**–**b4**) with same-side aryl substituents: (**a1**) (*3R,3′S*), (**a2**) (*3S,3′R*), (**a3**) (*3R,3′R*), (**a4**) (*3S,3′S*); (**b1**) (*2R,3S*), (**b2**) (*2S,3R*), (**b3**) (*2R,3R*), (**b4**) (*2S,3S*).

To synthesize spiro-oxindole-β-lactams with the additional spiro conjugation, it seemed convenient to use N-substituted 2-oxopyrrolidine-3-carboxilic acids as starting reagents, introducing them to the Staudinger ketene-imine cycloaddition reactions as a source of ketenes. We started our research with the comparison of the results of docking to the MDM2 binding site for the forming in such reactions dispiro compound **a** and the previously synthesized [27] spiro derivative **b** with the same set of exocyclic aryl substituents (see Table 1). According to docking studies conducted with AutoDock, Vina’s [30] introduction of the N-aryl-2-oxopyrrolidine moiety provides a noticeable increase in affinity to the binding site of MDM2 compared to previously studied spiro-β-lactams [27] (Figure 2, Table 1).

It can be seen from Figure 2 that *cis*-diastereomer of spirooxindole (**b1** (*2R,3S*) and **b2** (*2S,3R*)) is better at occupying the deepest pocket of binding site than the*cis*-diastereomer of dispirooxindole-β-lactam (**a1** (*3R,3′S*) and **a2** (*3S,3′R*)).However, due to the additional N-aryl-2-oxopyrrolidine ring, dispirooxindole-β-lactam **a** can cover a larger surface area of the binding pocket compared to their mono-spiro analogues. This results in higher affinity even for less plausible *cis*-diastereomer (Table 1).

**Scheme 1:**
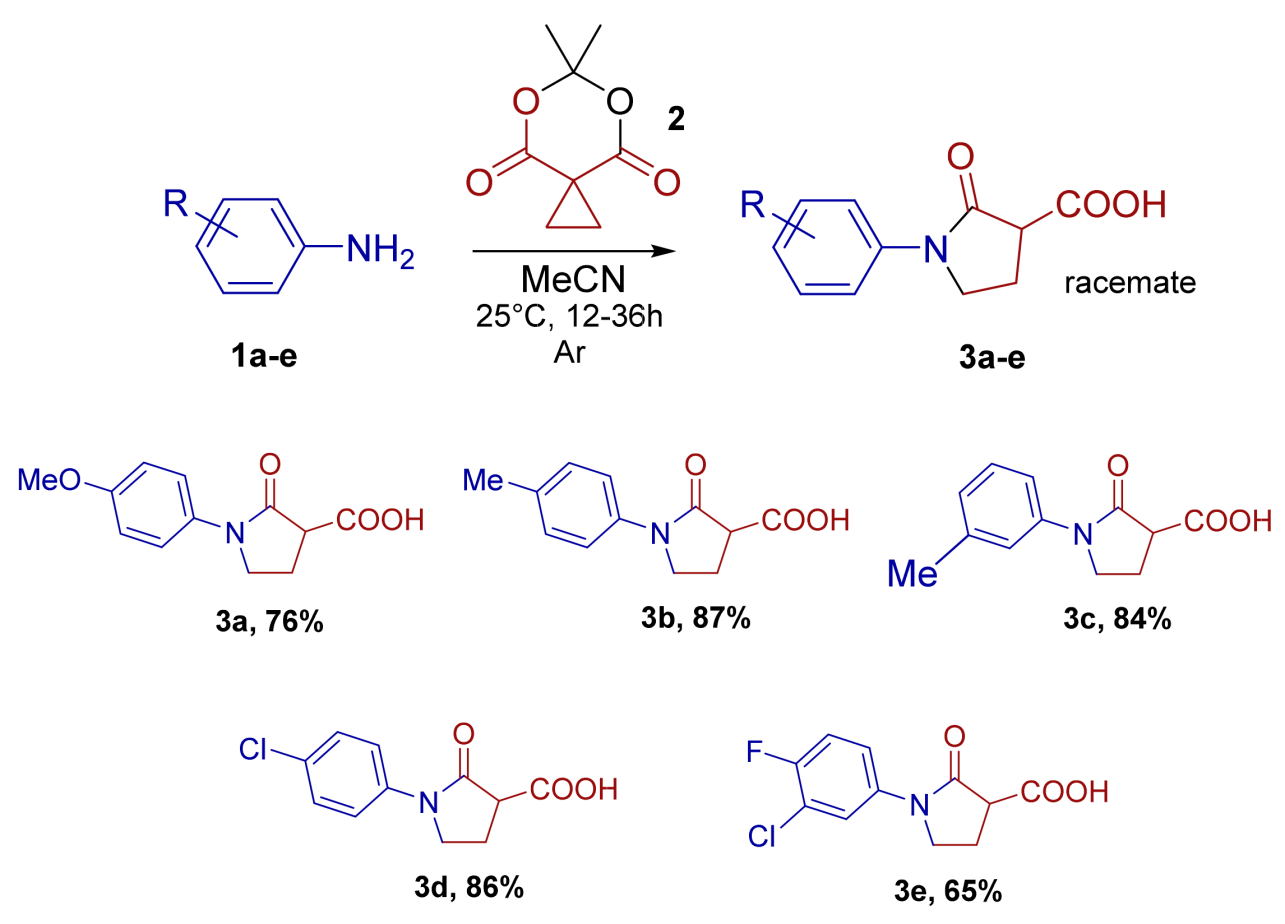
Synthesis of N-aryl substituted 2-oxopyrrolidine-3-carboxilic acids (**3a**–**e**) through homoconjugate addition of anilines (**1a**–**e**) to cyclopropane-1,1-dicarboxylate **2**.

The starting N-substituted 2-oxopyrrolidine-3-carboxilic acids for the subsequent Staudinger keten-imine cycloaddition were synthesized using slightly modified experimental techniques described in [31,32,33,34]. In general, all these methodologies are variations on thehomoconjugate addition of nucleophiles to cyclopropane-1,1-dicarboxylate derivatives reported by Rajendra K. Singh and Samuel Danishefsky [35]. We implemented this approach to the synthesis of a series of N-aryl-2-oxopyrrolidine-3-carboxilic acids **3a**–**e** from anilines **1a**–**e** and cyclopropane-1,1-dicarboxylate **2** (Scheme 1), which have not been previously described.

Initial cyclopropane-1,1-dicarboxylate **2** was prepared using methodologies reported in [35,36,37]. The addition of anilines **1a**–**e** to **2** proceeds under mild conditions, resulting in the formation of products **3a**–**e** with good yields.

**Scheme 2:**
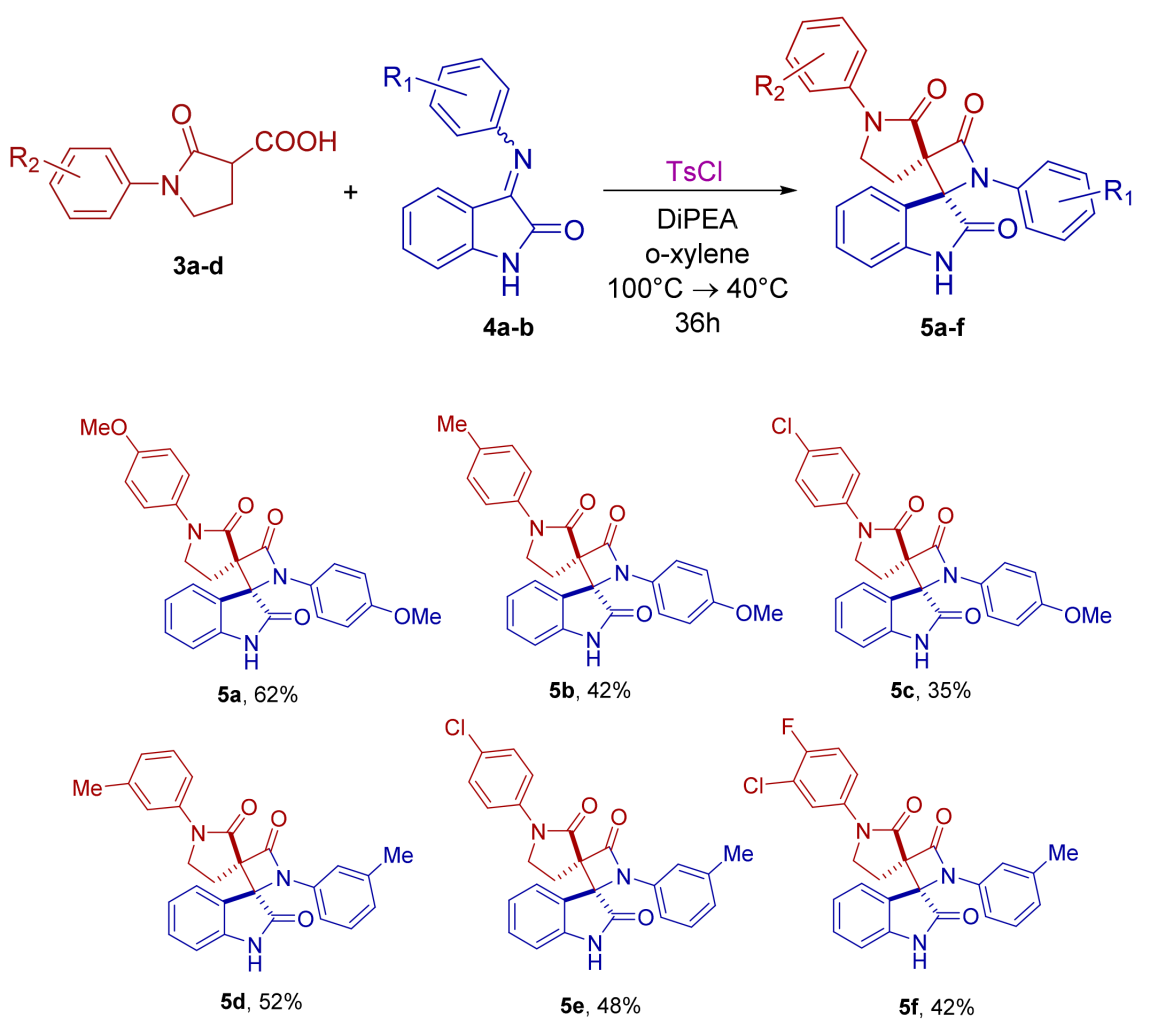
Synthesis of *bis*-aryl spirooxindole-β-lactams via Staudinger ketene-imine cycloaddition using N-aryl-2-oxopyrrolidine-3-carboxilic acids **3** as a source of ketene.

Next, compounds **3a**–**e** were introduced into *one-pot* Staudinger ketene-imine cycloaddition reactions with isatinimines **4a**,**b**. Reacting ketenes were produced from keto-acids **3** in situ using TsCl as an activating co-reagent (Scheme 2). 

For this procedure, we implemented the previously used diastereoselective synthesis of bis-aryl spirooxindole-β-lactams [38]. After slight optimization of reaction conditions, namely solvent and reaction temperature selection, we obtained f series novel dispirooxindole-β-lactams **5a**–**f** in reasonable yields.

### 2.2. Structural Determination of Compounds **5** and Other Reaction Products

**Figure 3:**
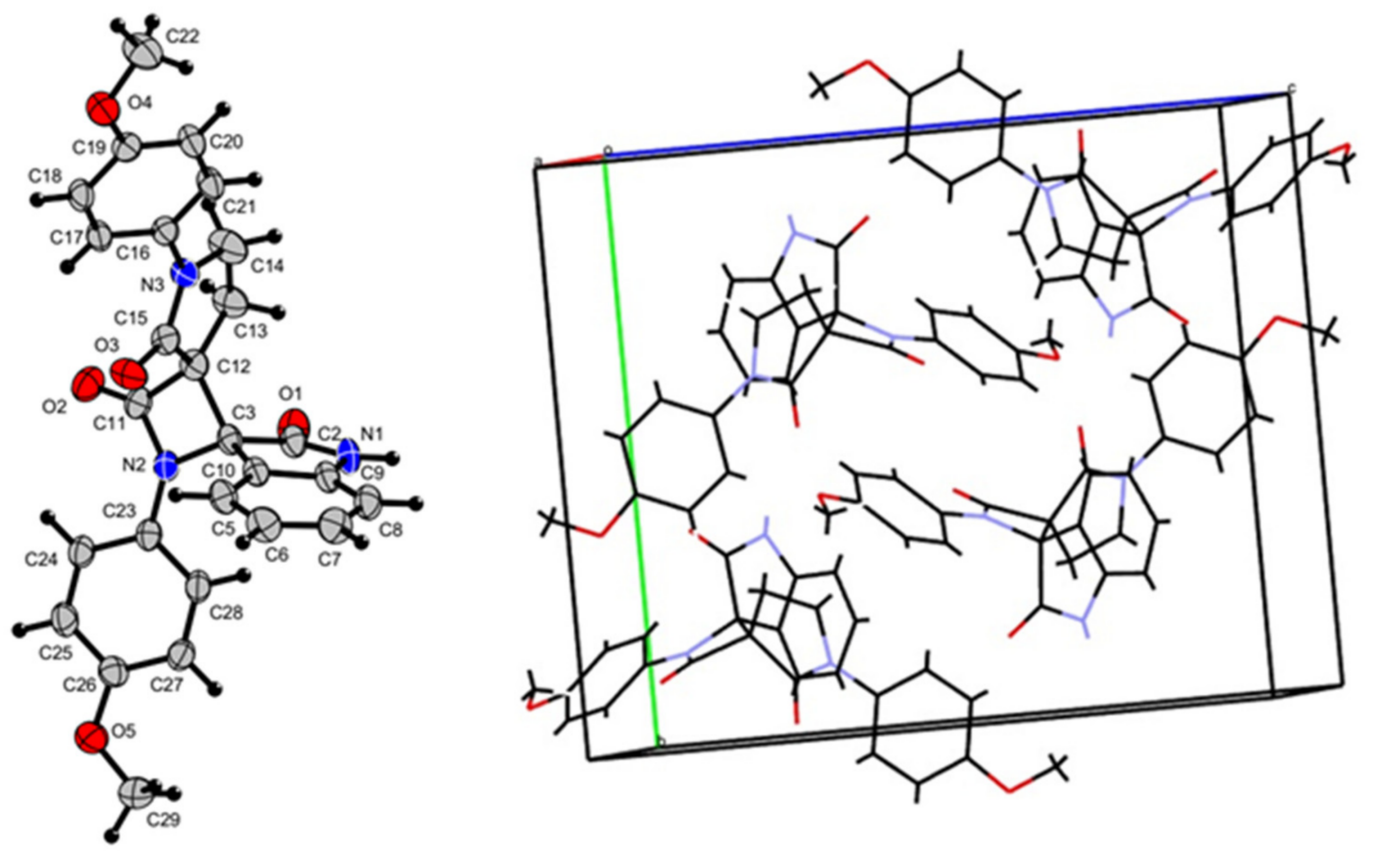
Molecular structure and unit cell of compound **5a**.

In order to establish the structure of synthesized compounds and evaluate diastereoselectivity of process, we conducted the detail studies of compound **5a**. Dispiro-β-lactam **5a** forms monoclinic unit cell belonging to space group P 21/n according to X-ray analysis data (Figure 3).

The molecular structure of compound **5a** corresponds to *trans*-configuration of substituents adjacent to β-lactam ring or (3R*,*3*′R*)-1′,1″-bis(4-methoxyphenyl)dispiro[indoline-3,2′-azetidine-3′,3″-pyrrolidine]-2,2″,4′-trione (Scheme 1, Figure 3). The four-membered β-lactam cycle and the spiro-linked five-membered rings are practically planar. The neighboring spiro-jointed cycles are practically perpendicular to each other (Table 2).

**Figure 4:**
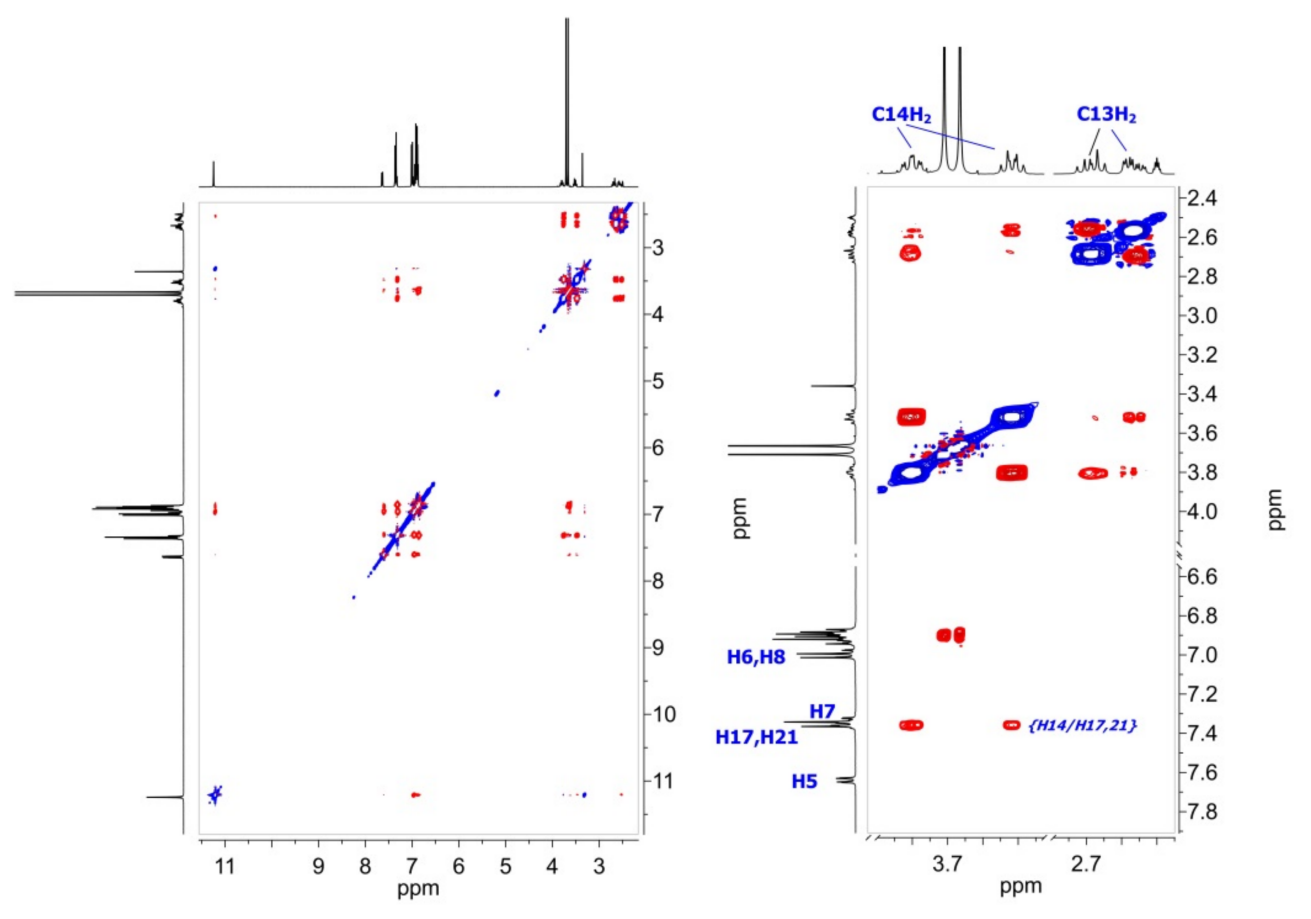
ROESYAD spectra of compound **5a**. Atom labels correspond to the crystal structure from Figure 3.

The structure of compound **5a** obtained for a single crystal was also confirmed by NMR data for a DMSO solution. Spectral assignments were achieved using 2D correlation techniques, gCOSY, ROESYAD, gHSQCAD, and gHMBCAD (see  Supplementary Information Figures S12 and S13). Among the most important results of NMR research, note that the Overhauser effect was observed for the interaction between the protons of the dimethylene fragment of the pyrrolidinone ring, even between the most distant protons (the maximum distance is 3.06 Å on the *trans*-isomer of compound **5a**). However, there is no cross peak in the ROESYAD spectrum (Figure 4) corresponding to the interaction of methylene protons with the most closely located aromatic proton of indolin-2-one due to considerable distance (>5 Å) between pyrrolidine one and isatin protons. This interaction is to be expected for *cis*-diastereomer where the corresponding distance should be ≤3 Å.

Thus, it can be argued that the reaction of N-aryl-2-oxopyrrolidine-3-carboxilic acids 3a-e with isatinimines in the presence of tosyl chloride proceeds diastereospecifically, yielding only the *trans*-diastereomer of dispiro-β-lactams **5**. 

**Figure 5:**
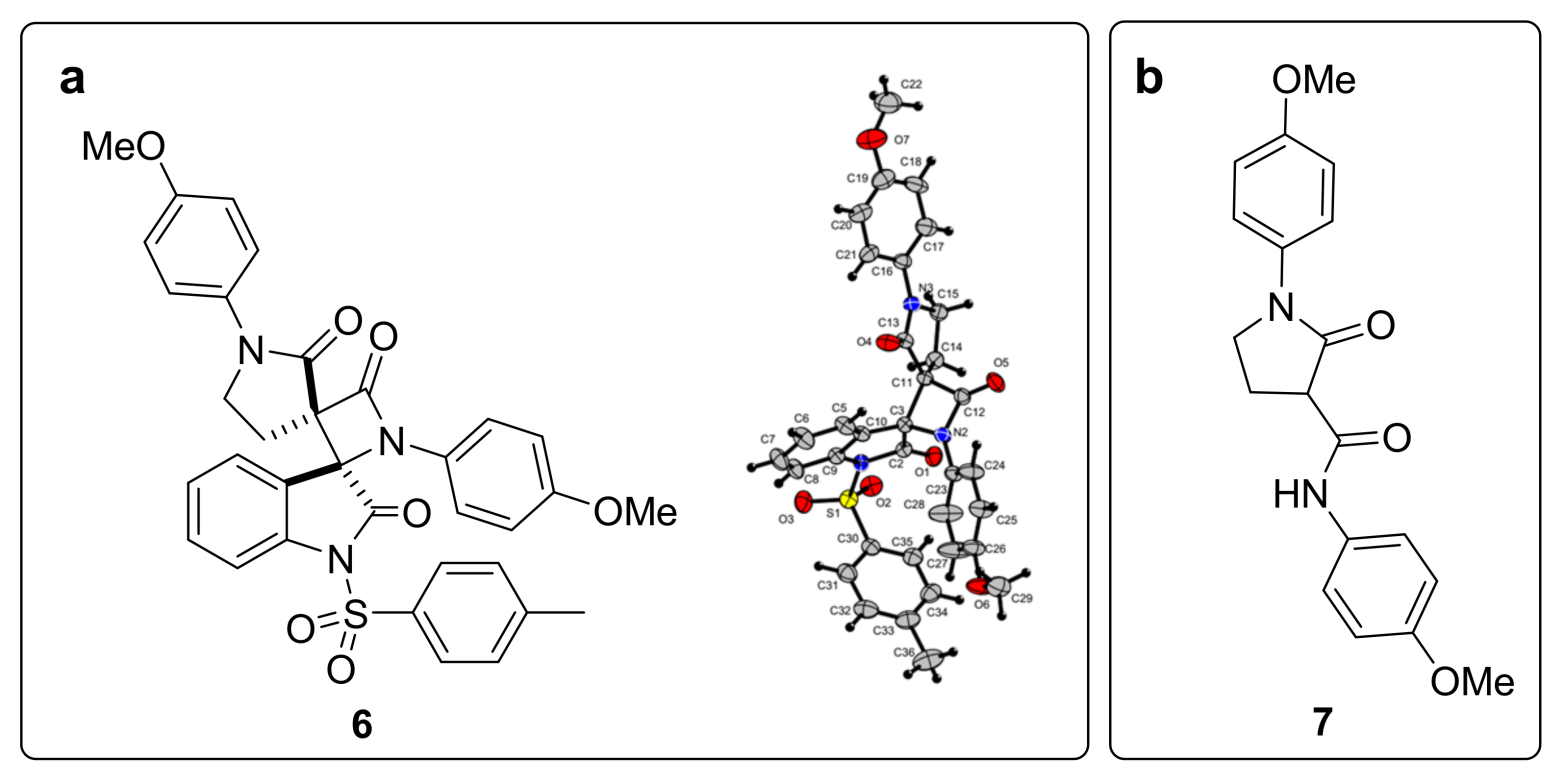
By-products of compound **5a** synthesis: (**a**) *p*-toluene sulfonylamide **6**; (**b**) N,1-bis(4-methoxyphenyl)-2-oxopyrrolidine-3-carboxamide **7**.

Additionally, we managed to isolate some by-products of the acid **3a**’s interaction with isatinimines and TsCl, namely *p*-toluene sulfonylamide **6** and N,1-bis(4-methoxyphenyl)-2-oxopyrrolidine-3-carboxamide **7** (Figure 5). These compounds were isolated from the reaction mixtures in amounts not exceeding 5–7%. The structure of compound **6** was confirmed by X-ray diffraction data.

Compound **6** is a product of the isatin amide group tosylation of the dispiro-β-lactam **5a**. The formation of tosylated product **6** in the form of only *trans*-diastereomer can also serve as an additional confirmation of the diastereoselectivity of the formation of dispiro-β-lactam moieties. Compound **7** is possibly a product of the hydrolysis of starting isatinimine followed by the reaction of the formed aniline **1a** and acid **3a** with amide formation. 

**Figure 6:**
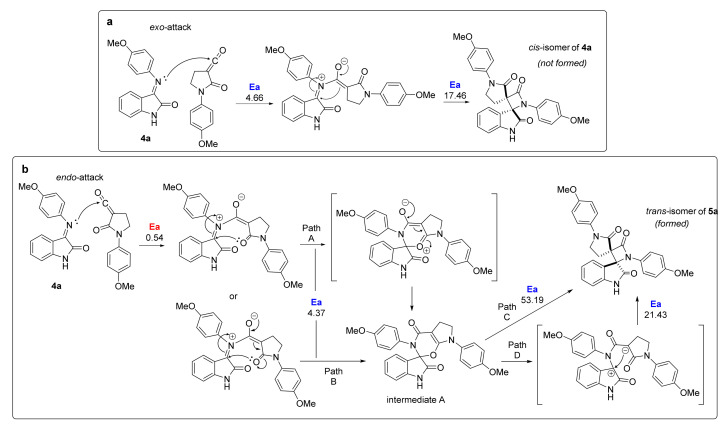
The presumable reaction mechanism for the compound **5a**’s formation and calculated activation barrier values (Ea, kcal/mol) for the *exo-* (**a**) and *endo-* (**b**) attack of *E*-imine **4a** on ketene carbonyl group with the subsequent cyclization of the resulting zwitterionic intermediate.

To explain the observed stereochemical outcome, we applied quantum chemical calculations of the two-stage process of ketene–imine cycloaddition, with a consistent nucleophilic addition of imine nucleophile to the ketene carbonyl droup in two possible addition modes—*exo*- or *endo*-attack, followed by cyclization of resulting zwitterion intermediate (Figure 6).

Geometrical optimization for the compounds, vibrational frequency analysis, and transition state (TS) searches of the studied transformations and intrinsic reaction coordinate calculations (IRC) were carried out using PRIRODA-04 software [39] with the Perdew–Burke–Ernzerhof (PBE) [40] functional in combination with a polarization-consistent basis set (PC-1) for the model reaction of compound **5a** synthesis.

While the *E*-imine *exo*-attack pathway showed conventional energy profiles with two activation barriers corresponding to the formation of the zwitterionic intermediate and its cyclization (Figure 6a), the *endo*-attack calculation showed unexpected details of the reaction mechanism. First, compared with the *exo*-attack of *E*-imine, which proceeds with a relatively low activation barrier, the formation of zwitterion through the *endo*-attack begins without barriers at all (Figure 6b; the value of 0.54 kcal/mol lies within the accuracy of the calculation). However, then, the resulting zwitterion can be cyclized to form the *cis*-isomer of dispiro-β-lactam **5a** only with a sufficiently high activation energy (53.19 kcal/mol), which limits the spontaneous process. In contrast, the zwitterion formed as a result of the *E*-imine *endo*-attack (Figure 6b) does not cyclize directly to *trans*-β-lactam, but undergoes an alternative cyclization via a nucleophilic attack of the lone pair of the amide oxygen of the pyrrolidin-2-one ring on the iminium carbon atom. This leads to the formation of intermediate **A** (Figure 6b) in two possible ways: through the formation of a spirocyclic zwitterion (Figure 6b, path A) or by the synchronous rearrangement of the initial zwitterion (Figure 6b, path B). This stage proceeds with a significantly low activation energy and stabilizes the spatial configuration of the substituents, promoting the formation of the *trans*-isomer of dispiro-β-lactam. Although direct cyclization of intermediate **A** via the 1,3-oxazinon ring closure is also possible (Figure 6b, path C), it is less energetically favorable than the two-step process of opening the 1,3-oxazinon compound and the subsequent cyclization of the resulting zwitterion (Figure 6b, path D). The activation barrier of the latter conversion is comparable to the conventional zwitterion intermediate cyclization demonstrated for the*exo*-attack (Figure 6a).

Thus, it was observed that the stereochemical outcome where the reaction yields only *trans*-diastereomer of **5a** may be due to barrierless formation of the zwitterion undergoing subsequent stabilization through the formation of a cyclic intermediate with low activation energy.

### 2.3. Biological Investigations of Compounds **5**

We carried out in vitro cytotoxicity tests for synthesized dispiroindolin-2-ones **5a**–**f**. Cytotoxicity was evaluated using the standard MTT method [41] using Mcf7 (breast adenocarcinoma), A549 (lung cancer), Hek293t (derivative human cell line that expresses a mutant version of the SV40 large T antigen), and VA13 (noncancerous epithelial lung fibroblasts) cell lines. For comparison, we also studied the cytotoxicity on these cell lines of some spiro-β-lactams with one spiro-junction, which we synthesized earlier [27,38]. The results are shown in Table 3.

According to the data presented in Table 3, dispirooxindole-β-lactam **5a** is the least potent of the series, possibly meaning that the stereo configuration provided by the two side 4-MeO-C_6_H_4_ substituents is less complementary to a target binding site. A similar effect is observed in the case of **5b**. This allows to conclude that large lipophilic substituents in the fourth position of the aryl side rings hinder the binding of dispirooxindole to the active site of the target. Meanwhile, placing smaller lipophilic substituents in the fourth position of even one aryl ring drastically increases cytotoxicity. It can also be seen that the introduction of 3-substituted instead of 4-substituted aryl side rings results in relatively high toxicity values. It is important to notice that the very same correlation was previously observed for *bis*-aryl spirooxindole-β-lactams [27]. The most essential result is that some dispirooxindole-β-lactams demonstrated a higher antiproliferative activity compared to oxindole-β-lactams with single spiroconjugation despite the formation of less potent (according to docking studies; see Table 1) diastereomers. Thus, the introduction of an additional functionalized spiro-conjugated ring will improve the cytotoxic activity of oxindole β-lactam derivatives with the same aryl substituents in spiro-linked rings.

Synthesized dispiroindolin-2-ones **5a**–**f** were also tested for antibacterial activity. Tests were carried out on two *E. coli* strains that were hypersensitive to antibiotics, *DTC* [42] and *LPTD* [43]. *E. coli DTC* strain lacks tolC gene, which encodes the outer membrane component of several multidrug transporters [44]. *E. coli LPTD* has the 23-amino-acid deletion in lptD gene, anessential protein functioning in the final stages of the assembly of lipopolysaccharides into the outer membrane [45]. On a plate test, dispirooxindole-β-lactam provided with 3-Me-C_6_H_4_ and 4-F,3-Cl-C_6_H_3_ **5f** showed a small amount of activity on the LPTD strain. Then, minimal inhibitory concentration was measured, and for **5f,** it was 1,30 ± 0,45 μM; compounds **5a**–**e** did not show any activity even at high concentrations. Absence of **5f** activity on *E. coli DTC* can be explained by low penetration of this molecule in cells with normal lptD protein. It correlates with higher LogP of the one bacteriotoxic compound **5f** in the series. Compound **5f** was analyzed for the mechanism of antibacterial activity by means of the pDualrep2 [46] reported system, which allows to detect translational inhibitors and SOS-response inducers, but none of these mechanisms is involved.

## 3. Materials and Methods

### 3.1. General Information

All solvents used were purified and dehydrated using the methods described in [47]. All starting reagents were purchased from commercial sources (Sigma-Aldrich, ABCR, AKSci, Burlington, VT, USA). Microwave-heated reactions for the preparation of isatinimines were performed in a Monowave 300—Anton Paar reactor in sealed glass vessels with external temperature control. Reactions were checked by TLC analysis using silica plates with a fluorescent indicator (254 nm) and visualized with a UV lamp. ^1^H and ^13^C NMR spectra were recorded on a BrukerAvance and Agilent 400-MR spectrometers (400 MHz for ^1^H, 100 MHz for ^13^C) in DMSO-d_6_. Chemical shifts are reported in parts per million relative to TMS. IR spectra were recorded using a Fourier transform spectrometer Nicolet iS5 (Thermo Fisher Scientific, Waltham, MA, USA) using an internal reflectance attachment with a diamond optical element—attenuated total reflection (ATR, iD7) with 45o angle of incidence. Resolution was 4 cm^−1^, and the number of scans is 20.

Electrospray ionization high-resolution mass spectra were recorded in positive ion mode on a TripleTOF 5600+ quadrupole time-of-flight mass spectrometer (ABSciex, Concord, Vaughan, ON, Canada) equipped with DuoSpray ion source. The following MS parameters were applied: capillary voltage 5.5 kV; nebulizing and curtain gas pressure—15 and 25 psi, respectively; ion source temperature—ambient; declustering potential 20 V; *m*/*z* range 100–1200. Elemental compositions of the detected ions were determined based on accurate masses and isotopic distributions using Formula Finder software (ABSciex, Concord, ON, Canada). The maximum allowed deviation of the experimental molecular mass from the calculated one was 5 ppm.

The X-ray data for the compounds **5a** and **6** were collected at room temperature using STOE diffractometer Pilatus100K detector, focusing mirror collimation CuKα (1.54086Å) radiation, rotation method mode. STOE X-AREA software was used for cell refinement and data reduction. Data collection and image processing wereperformed with X-Area 1.67 (STOE &Cie GmbH, Darmstadt, Germany, 2013). Intensity data were scaled with LANA (part of X-Area) in order to minimize differences in theintensities of symmetry-equivalent reflections (multi-scan method). The structures were solved with SHELXT and refined with SHELX. The non-hydrogen atoms (for both substances) were refined by using the anisotropic full-matrix least-square procedure. All hydrogen atoms were placed in the calculated positions and allowed to ride on their parent atoms (C-H 0.93–0.98; Uiso 1.2 Ueq (parent atom)).

### 3.2. General Procedure for thePreparation of 6,6-Dimethyl-5,7-Dioxaspiro [2.5]Octane-4,8-Dione (2)

Starting 6,6-dimethyl-5,7-dioxaspiro[2.5]octane-4,8-dione **2** was prepared according to synthetic procedures reported in [35,36,37].

### 3.3. General Procedure for Preparation of Isatinimines **4a**,**b**

Iminoindolin-2-ones **4a**,**b** were prepared according to the synthetic procedures reported in [38].

### 3.4. General Procedure for Preparation of N-Aryl-2-Oxopyrrolidine-3-Carboxylic Acids **3a**–**e**

Acids **3a**–**e** were synthesized according to slightly modified procedures, described in [31,48]. A cyclopropane-1,1-dicarboxylate **2** (4 mmol) was dissolved at room temperature in anhydrous acetonitrile (10 mL) in a round-bottom flask. The resulting solution was blown through with dry argon for 10 min. Then, substituted aniline (8 mmol) was added at room temperature, and additional dry argon was bubbled through the reaction solution for 30 min. The reaction mixture was then stirred for 48 h at room temperature. An additional control of the reaction completion via TLC may be employed. The solvent was then removed under reduced pressure to afford a beige to brown solid product. This solid was dissolved in dichloromethane (30 mL) and washed with two portions of 10% HCl (10 mL). The organic layer was separated and dried with Na_2_SO_4_.The solvent was then removed under reduced pressure to afford a white to beige solid product. An additional purification via column chromatography using petroleum ether-ethyl acetate (2:1) as eluent can be employed to provide N-aryl-2-oxopyrrolidine-3-carboxylic acids of higher purity.

2-Oxo-1-(4-methoxyphenyl)-pyrrolidine-3-carboxylic acid (**3a**) [48]. A reaction of 4-methoxyaniline **1a** (985 mg, 8 mmol) and cyclopropane-1,1-dicarboxylate **2** (681 mg, 4 mmol) yields 1-(p-methoxyphenyl)-2-oxopyrrolidine-3-carboxylic acid as a beige solid (715 mg, 76%).

^1^H NMR (400 MHz, Chloroform-*d*) δ 9.57 (br.s, 1H), 7.49–7.42 (m, 2H), 6.96–6.89 (m, 2H), 3.95–3.82 (m, 2H), 3.81 (s, 3H), 3.64 (t, *J* = 9.9 Hz, 1H), 2.62–2.41 (m, 2H).

^13^C NMR (101 MHz, Chloroform-*d*) δ 170.6, 169.1, 157.3, 130.5, 122.0, 113.9, 55.1, 47.1, 46.9, 20.5.

2-Oxo-1-(4-methylphenyl)pyrrolidine-3-carboxylic acid (**3b**) [31]. A reaction of p-toluidine **1b** (857 mg, 8 mmol) and cyclopropane-1,1-dicarboxylate **2**(681 mg, 4 mmol) yields 2-oxo-1-(p-tolyl)pyrrolidine-3-carboxylic acid as a white solid (763 mg, 87%).

^1^H NMR (400 MHz, Chloroform-*d*) δ 9.87 (s, 1H), 7.43 (d, *J* = 8.5 Hz, 2H), 7.20 (d, *J* = 8.3 Hz, 2H), 3.95–3.83 (m, 2H), 3.64 (t, *J* = 9.7 Hz, 1H), 2.60–2.44 (m, 2H), 2.34 (s, 3H).

^13^C NMR (101 MHz, Chloroform-*d*) δ 171.2, 169.9, 136.1, 135.6, 129.8, 120.8, 47.8, 47.4, 21.2, 21.1.

2-Oxo-1-(3-methylphenyl)pyrrolidine-3-carboxylic acid (**3c**) [48]. A reaction of m-toluidine **1c** (857 mg, 8 mmol) and cyclopropane-1,1-dicarboxylate **2** (681 mg, 4 mmol) yields 2-oxo-1-(m-tolyl)pyrrolidine-3-carboxylic acid as a beige solid (737 mg, 84%).

^1^H NMR (400 MHz, Chloroform-*d*) δ 10.22 (s, 1H), 7.37 (s, 1H), 7.32 (d, *J* = 8.3 Hz, 1H), 7.24 (t, *J* = 7.7 Hz, 1H), 7.01 (d, *J* = 7.5 Hz, 1H), 3.91–3.79 (m, 2H), 3.64 (t, *J* = 9.2 Hz, 1H), 2.50–2.39 (m, 2H), 2.34 (s, 3H).

^13^C NMR (101 MHz, Chloroform-*d*) δ 171.0, 170.7, 139.0, 138.1, 128.8, 126.7, 121.4, 117.8, 48.5, 47.5, 21.5, 21.5.

2-Oxo-1-(4-chlorophenyl)-pyrrolidine-3-carboxylic acid (**3d**) [31]. A reaction of 4-chloroaniline **1d** (1021 mg, 8 mmol) and cyclopropane-1,1-dicarboxylate **2** (681 mg, 4 mmol) yields 1-(4-chlorophenyl)-2-oxopyrrolidine-3-carboxylic acid as a white solid (824 mg, 86%).

^1^H NMR (400 MHz, Chloroform-*d*) δ 7.55 (d, *J* = 8.9 Hz, 2H), 7.38 (d, *J* = 8.8 Hz, 2H), 3.90 (q, *J* = 9.7, 8.7 Hz, 2H), 3.66 (t, *J* = 9.8 Hz, 1H), 2.66–2.44 (m, 2H).

^13^C NMR (101 MHz, DMSO-*d*_6_) δ 171.4, 131.5, 129.4, 121.7, 47.7, 47.1, 21.0.

2-Oxo-1-(3-chloro-4-fluorophenyl)-pyrrolidine-3-carboxylic acid (**3e**) [31]. A reaction of 3-chloro-4-fluoroaniline **1e** (1164 mg, 8 mmol) and cyclopropane-1,1-dicarboxylate **2** (681 mg, 4 mmol) yields 1-(3-chloro-4-fluorophenyl)-2-oxopyrrolidine-3-carboxylic acid as a white solid (670 mg, 65%).

^1^H NMR (400 MHz, DMSO-*d*_6_) δ 12.85 (s, 1H), 7.94 (dd, *J* = 6.7, 2.7 Hz, 1H), 7.62 (ddd, *J* = 9.1, 4.2, 2.8 Hz, 1H), 7.45 (t, *J* = 9.1 Hz, 1H), 3.93–3.78 (m, 2H), 3.64–3.58 (m, 1H), 2.42–2.24 (m, 2H).

^13^C NMR (101 MHz, DMSO-*d*_6_) δ 171.1, 169.9, 155.0, 152.6, 136.3, 121.4, 120.1, 120.0, 119.5, 119.3, 117.0, 116.7, 49.8, 46.8, 21.9.

### 3.5. General Procedure for Preparation of Dispirooxindole-β-Lactams **5a**–**f**

N-Aryl-2-oxo-pyrrolidine-3-carboxylic acid **3** (1.5 mmol) was dissolved in anhydrous *o*-xylene (15 mL) at room temperature. To the resulting solution N,N-diisopropylethylamine (DIPEA; 1.5 mmol) was added. The resulting mixture was heated to 40 °C and stirred at this temperature for 10 min. 4-Toluenesulfonyl chloride (1.6 mmol) was then added in one portion, and the resulting solution was heated to 100 °C, stirred for 30 min and then cooled down to 40 °C. The additional DIPEA (1.5 mmol) in *o*-xylene (15 mL) was added, and the reaction mixture was stirred for 5 min. Isatinimine solution in *o*-xylene (5 mL) was then added and the mixture was stirred at 40 °C for 48–72 h (a completion of the reaction was checked using TLC). The solvent was removed under reduced pressure to give a viscous oil, which was purified with gradient column chromatography using petroleum ether-ethyl acetate (2:1) to ethyl acetate as eluent. The resulting solid was recrystallized from diethyl ether to afford target compound.

(3S*,3′S*)-1′*,1″-di(4-Methoxyphenyl)dispiro[indoline-3,2*′*-azetidine-3*′*,3″-pyrrolidine]-2,2″,4*′*-trione (**5a**)*. A reaction of 3-((4-methoxyphenyl)imino)indolin-2-one **4a** (252 mg, 1 mmol), 1-(4-methoxyphenyl)-2-oxopyrrolidine-3-carboxylic acid **3a** (353 mg, 1.5 mmol), 4-toluenesulfonyl chloride (305 mg, 1.6 mmol) and DIPEA (388 mg, 523 mkl, 3 mmol) yields dispiro-β-lactam **5a** as a white solid (291 mg, 62%) and by-products **6** (44 mg, 7%) and **7** (7 mg, 2%).

*(**5a**)*^1^H NMR (400 MHz, DMSO-*d*_6_) δ 10.95 (s, 1H), 7.36–7.32 (m, 1H), 7.09–7.01 (m, 3H), 6.74–6.54 (m, 8H), 3.50 (ddd, *J* = 9.9, 8.7, 2.9 Hz, 1H), 3.41 (s, 3H), 3.37 (s, 3H), 3.22 (dt, *J* = 9.8, 7.6 Hz, 1H), 2.39 (dt, *J* = 14.4, 8.4 Hz, 1H), 2.26 (ddd, *J* = 14.5, 7.3, 2.8 Hz, 1H).

^13^C NMR (101 MHz, DMSO-*d*_6_) δ 174.1, 166.3, 163.0, 156.6, 156.2, 142.7, 131.4, 131.2, 129.4, 127.8, 122.0, 122.0, 118.2, 114.8, 114.0, 110.9, 71.6, 68.1, 55.3, 55.2, 45.9, 22.1.

IR (cm^−1^): 3208, 1769, 1732, 1675, 1510, 1246, 1022, 829.

HRMS (ESI) Calculated for C_27_H_24_N_3_O_5_^+^ [M + H+]: 470.1710, found: 470.1704.

*(3S,3*′*S)-1*′,*1*″*-bis(4-methoxyphenyl)-1-tosyldispiro[indoline-3,2*′*-azetidine-3*′,*3*″*-pyrrolidine]-2,2*″,*4*′*-trione (**6**)*^1^H NMR (400 MHz, DMSO-*d*_6_) δ 8.01 (d, *J* = 8.0 Hz, 1H), 7.97 (d, *J* = 8.4 Hz, 2H), 7.75 (dd, *J* = 7.7, 1.4 Hz, 1H), 7.58 (ddd, *J* = 8.3, 7.7, 1.4 Hz, 1H), 7.52 (dt, *J* = 8.6, 2.1 Hz, 2H), 7.36–7.31 (m, 2H), 7.26 (td, *J* = 7.7, 0.9 Hz, 1H), 6.93–6.88 (m, 2H), 6.62–6.57 (m, 2H), 6.47–6.42 (m, 2H), 3.79–3.73 (m, 1H), 3.72 (s, 3H), 3.65 (s, 3H), 3.43–3.37 (m, 1H), 2.69–2.58 (m, 2H), 2.42 (s, 3H), 2.27 (ddd, *J* = 14.5, 7.5, 3.2 Hz, 2H).

^13^C NMR (101 MHz, DMSO-*d*_6_) δ 172.3, 165.1, 156.4, 146.9, 139.1, 133.5, 132.2, 131.2, 130.5, 128.4, 128.0, 127.5, 125.2, 122.0, 119.8, 118.2, 114.6, 114.0, 113.7, 73.0, 55.3, 48.6, 45.6, 22.1, 21.2.

HRMS (ESI) Calculated for C_34_H_30_N_3_O_7_S^+^ [M + H+]: 624.1799, found: 624.1782.

*N,1-bis(4-methoxyphenyl)-2-oxopyrrolidine-3-carboxamide (**7**)*^1^H NMR (400 MHz, DMSO-*d*_6_) δ 10.14 (s, 1H), 7.56 (dd, *J* = 9.0, 4.3 Hz, 4H), 6.96 (d, *J* = 9.1 Hz, 2H), 6.90 (d, *J* = 9.0 Hz, 2H), 3.92–3.79 (m, 2H), 3.75 (s, 3H), 3.72 (s, 3H), 3.67 (d, *J* = 9.4 Hz, 1H), 2.46–2.27 (m, 2H).

HRMS (ESI) Calculated for C_19_H_21_N_2_O_4_^+^ [M + H+]: 341,1496, found: 341,1496.

(3S*,3′S*)-1′*-(4-Methoxyphenyl)-1*″*-(4-tolyl)dispiro[indoline-3,2*′*-azetidine-3*′,*3*″*-pyrrolidine]-2,2*″,*4*′*-trione (**5b**).* A reaction of 3-((4-methoxyphenyl)imino)indolin-2-one **4a** (252 mg, 1 mmol), 2-oxo-1-(4-tolyl)pyrrolidine-3-carboxylic acid **3b** (329 mg, 1.5 mmol), 4-toluenesulfonyl chloride (305 mg, 1.6 mmol) and DIPEA (388 mg, 523 mkl, 3 mmol) yields dispiro-β-lactam **5b** as a white solid (190 mg, 42%).

^1^H NMR (400 MHz, Chloroform-*d*) δ 11.24 (s, 1H), 7.62 (d, *J* = 7.5 Hz, 1H), 7.40–7.28 (m, 3H), 7.14 (d, *J* = 8.3 Hz, 2H), 7.02–6.94 (m, 2H), 3.82 (td, *J* = 9.8, 2.9 Hz, 1H), 3.67 (s, 3H), 3.59–3.48 (m, 1H), 2.74–2.62 (m, 1H), 2.56 (ddd, *J* = 14.5, 7.3, 2.9 Hz, 1H), 2.24 (s, 3H).

^13^C NMR (101 MHz, DMSO-*d*_6_) δ 174.1, 166.6, 156.3, 142.7, 135.9, 134.4, 131.2, 129.4, 129.3, 127.8, 122.1, 120.7, 120.1, 118.2, 114.8, 111.0, 71.8, 68.1, 55.3, 45.6, 22.1, 20.4.

IR (cm^−1^): 3247, 1763, 1683, 1509, 1241, 815.

HRMS (ESI) Calculated for C_27_H_24_N_3_O_4_^+^ [M + H+]: 454.1761, found: 454.1764.

(3S*,3′S*)-1″*-(4-Chlorophenyl)-1*′*-(4-methoxyphenyl)dispiro[indoline-3,2*′*-azetidine-3*′,*3″-pyrrolidine]-2,2″,4*′*-trione (**5c**).* A reaction of 3-((4-methoxyphenyl)imino)indolin-2-one **4a** (252 mg, 1 mmol), 1-(4-chlorophenyl)-2-oxopyrrolidine-3-carboxylic acid **3d** (359 mg, 1.5 mmol), 4-toluenesulfonyl chloride (305 mg, 1.6 mmol), and DIPEA (388 mg, 523 mkl, 3 mmol) yields dispiro-β-lactam **5c** as a white solid (166 mg, 35%).

^1^H NMR (400 MHz, DMSO-*d*_6_) δ 11.26 (s, 1H), 7.61 (d, *J* = 7.5 Hz, 1H), 7.58–7.53 (m, 2H), 7.44–7.38 (m, 2H), 7.33 (td, *J* = 7.8, 1.0 Hz, 1H), 6.98 (t, *J* = 7.6 Hz, 2H), 6.94–6.85 (m, 4H), 3.85 (td, *J* = 9.3, 8.8, 3.0 Hz, 1H), 3.66 (s, 3H), 3.63–3.54 (m, 1H), 2.75–2.64 (m, 1H), 2.58 (ddd, *J* = 14.4, 7.4, 3.0 Hz, 1H).

^13^C NMR (101 MHz, DMSO-*d*_6_) δ 174.0, 167.0, 162.7, 156.3, 142.8, 137.3, 131.28, 129.3, 129.0, 128.8, 127.7, 122.1, 121.5, 120.6, 118.3, 114.9, 111.0, 71.7, 68.1, 55.3, 45.5, 22.0.

IR (cm^−1^): 3230, 1763, 1713, 1684, 1510, 1493, 1376, 1241, 825, 753.

HRMS (ESI) Calculated for C_26_H_21_ClN_3_O_4_^+^ [M + H+]: 474.1215, found: 474.1210.

(3S*,3′S*)-1′,*1*″*-di(3-Tolyl)dispiro[indoline-3,2*′*-azetidine-3*′,*3*″*-pyrrolidine]-2,2*″,*4*′*-trione (**5d**).* A reaction of 3-(3-tolylimino)indolin-2-one **4b** (236 mg, 1 mmol), 2-oxo-1-(3-tolyl)pyrrolidine-3-carboxylic acid **3c** (329 mg, 1,5 mmol), 4-toluenesulfonyl chloride (305 mg, 1,6 mmol), and DIPEA (388 mg, 523 mkl, 3 mmol) yields dispiro-β-lactam **5d** as a white solid (227 mg, 52%).

^1^H NMR (400 MHz, DMSO-*d*_6_) δ 11.29 (s, 1H), 7.61 (d, *J* = 7.5 Hz, 1H), 7.35 (t, *J* = 7.7 Hz, 1H), 7.30 (d, *J* = 8.4 Hz, 1H), 7.22 (dd, *J* = 14.4, 6.6 Hz, 2H), 7.15 (t, *J* = 7.9 Hz, 1H), 7.05–6.95 (m, 4H), 6.92 (d, *J* = 7.6 Hz, 1H), 6.57–6.49 (m, 1H), 3.82 (td, *J* = 10.1, 2.5 Hz, 1H), 3.60–3.50 (m, 1H), 2.74–2.64 (m, 1H), 2.58 (ddd, *J* = 14.5, 7.1, 2.5 Hz, 1H), 2.25 (s, 3H), 2.20 (s, 3H).

^13^C NMR (101 MHz, DMSO-*d*_6_) δ 174.0, 166.6, 163.3, 142.6, 139.1, 138.3, 138.2, 136.1, 131.3, 129.4, 128.7, 127.7, 125.9, 125.5, 122.1, 120.8, 117.5, 117.4, 113.0, 111.0, 71.7, 67.9, 45.7, 22.2, 21.1.

IR (cm^−1^): 3206, 1767, 1716, 1684, 1357, 1237, 1209, 759, 684.

HRMS (ESI) Calculated for C_27_H_24_N_3_O_3_^+^ [M + H+]: 438.1812, found: 438.1805.

(3S*,3′S*)-1″*-(4-Chlorophenyl)-1*′*-(3-tolyl)dispiro[indoline-3,2*′*-azetidine-3*′,*3*″*-pyrrolidine]-2,2*″,*4*′*-trione (**5e**).* A reaction of 3-(3-tolylimino)indolin-2-one **4b** (236 mg, 1 mmol), 1-(4-chlorophenyl)-2-oxopyrrolidine-3-carboxylic acid **3d** (359 mg, 1.5 mmol), 4-toluenesulfonyl chloride (305 mg, 1.6 mmol), and DIPEA (388 mg, 523 mkl, 3 mmol) yields dispiro-β-lactam **5e** as a white solid (220 mg, 48%).

^1^H NMR (400 MHz, DMSO-*d*_6_) δ 11.30 (s, 1H), 7.60 (d, *J* = 7.6 Hz, 1H), 7.55 (d, *J* = 9.0 Hz, 2H), 7.41 (d, *J* = 9.0 Hz, 2H), 7.34 (t, *J* = 7.7 Hz, 1H), 7.15 (t, *J* = 7.9 Hz, 1H), 6.99 (dd, *J* = 16.7, 8.1 Hz, 3H), 6.92 (d, *J* = 7.6 Hz, 1H), 6.52 (d, *J* = 8.3 Hz, 1H), 3.85 (td, *J* = 9.6, 2.7 Hz, 1H), 3.58 (q, *J* = 7.7 Hz, 1H), 2.71 (dt, *J* = 16.4, 8.3 Hz, 1H), 2.59 (ddd, *J* = 14.5, 7.3, 2.8 Hz, 1H), 2.19 (s, 3H).

^13^C NMR (101 MHz, DMSO-*d*_6_) δ 173.9, 166.9, 163.2, 142.6, 139.1, 137.2, 136.1, 131.3, 129.4, 128.8, 127.7, 125.5, 122.1, 121.5, 120.5, 117.4, 113.1, 111.0, 71.6, 67.9, 45.5, 22.0, 21.1.

IR (cm^−1^): 3288, 1743, 1731, 1699, 1492, 1372, 1245, 1210, 754, 685.

HRMS (ESI) Calculated for C_26_H_21_ClN_3_O_3_^+^ [M + H+]: 458.1266, found: 458.1257.

(3S*,3′S*)-1″*-(3-Chloro-4-fluorophenyl)-1*′*-(3-tolyl)dispiro[indoline-3,2*′*-azetidine-3*′,*3*″*-pyrrolidine]-2,2*″,*4*′*-trione (**5f**).* A reaction of 3-(3-tolylimino)indolin-2-one **4b** (236 mg, 1 mmol), 1-(3-chloro-4-fluorophenyl)-2-oxopyrrolidine-3-carboxylic acid **3e** (386 mg, 1.5 mmol), 4-toluenesulfonyl chloride (305 mg, 1.6 mmol), and DIPEA (388 mg, 523 mkl, 3 mmol) yields dispiro-β-lactam **5f** as a white solid (200 mg, 42%).

^1^H NMR (400 MHz, DMSO-*d*_6_) δ 11.31 (s, 1H), 7.75 (dd, *J* = 6.6, 2.6 Hz, 1H), 7.59 (d, *J* = 7.5 Hz, 1H), 7.50 (ddd, *J* = 9.1, 4.2, 2.7 Hz, 1H), 7.41 (t, *J* = 9.1 Hz, 1H), 7.35 (td, *J* = 7.7, 1.0 Hz, 1H), 7.15 (t, *J* = 7.9 Hz, 1H), 7.00 (dd, *J* = 13.2, 7.2 Hz, 3H), 6.92 (d, *J* = 7.7 Hz, 1H), 6.52 (d, *J* = 8.9 Hz, 1H), 3.86 (td, *J* = 9.4, 9.0, 2.7 Hz, 1H), 3.65–3.55 (m, 1H), 2.70 (dt, *J* = 14.8, 8.4 Hz, 1H), 2.59 (ddd, *J* = 14.4, 7.2, 2.8 Hz, 1H), 2.20 (s, 3H).

^13^C NMR (101 MHz, DMSO-*d*_6_) δ 173.9, 167.0, 163.0, 142.7, 139.2, 136.1, 135.4, 131.4, 129.5, 127.6, 125.6, 122.2, 122.0, 120.8, 120.7, 120.5, 117.4, 117.2, 117.0, 113.08, 111.1, 71.5, 67.9, 45.7, 22.0, 21.1.

IR (cm^−1^): 3264, 1738, 1695, 1495, 1382, 1230, 1215, 685.

HRMS (ESI) Calculated for C_26_H_20_ClFN_3_O_3_^+^ [M + H+]: 476.1172, found: 476.1163.

### 3.6. Cell Lines

Human breast cancer cell line MCF7 and human lung adenocarcinoma cell line A549 were kindly provided by Dr. S. Dmitriev, immortalized human fibroblasts cell line VA13 was kindly provided by Dr. M. Rubtsova, human embryonic kidney HEK293T cell line was kindly provided by Dr. E. Knyazhanskaya. MCF7, VA13, A549, and HEK293T cell lines were maintained in DMEM/F-12 (Thermo Fisher Scientific, Waltham, MA, USA) culture medium containing 10% fetal bovine serum (Thermo Fisher Scientific, USA) and 50 µg/mL penicillin and 0.05 mg/mL streptomycin at 37 °C (Thermo Fisher Scientific, USA) in 5% CO2. Medium F-12 (Paneco LLC, Russia) containing 10% fetal bovine serum (Thermo Fisher Scientific, USA), 50 µg/mL penicillin, and 0.05 mg/mL streptomycin (Thermo Fisher Scientific, USA) was used instead of DMEM/F-12 in some cytotoxicity assays. PC-3 cell line (ATCC) was cultured in RPMI-1640 medium supplemented with 10% fetal bovine serum (FBS) and 2 mM L-glutamine (Gibco, New York, NY, USA). Cells were maintained at 37 °C in a humidified incubator MCO-18AC (Sanyo, Osaka, Japan) supplied with 5% CO_2_. After attaining 80% confluence, the cells were harvested with TrypLE (Gibco) and subcultured 1:8. Cell cultures were tested for the absence of mycoplasma.

### 3.7. In Vitro Survival Assay (MTT Assay)

The cytotoxicity of tested substances was tested using the MTT (3-(4,5-dimethylthiazol-2-yl)2,5-diphenyl tetrazolium bromide) assay [39] with some modifications. A total of 2500 cells per well for the MCF7, HEK293T, and A549 cell lines, or 4000 cells per well for the VA-13 cell line, were plated out in 135 µL of DMEM-F12 media (Gibco, USA) in a 96-well plate and incubated in the 5% CO_2_ incubator for first 16 h without treating. Then, 15 µL of media-DMSO solutions of tested substances to the cells (final DMSO concentrations in the media were 1% or less) and treated cells 72 h with 50 nM–100 µM (eight dilutions) of our substances (triplicate each) and doxorubicin as a control substance. The MTT reagent (Paneco LLC, Moscow, Russia) was then added to cells up to final concentration of 0.5 g/L (10 × stock solution in PBS was used) and incubated for 2 h at 37 °C in the incubator, under an atmosphere of 5% CO_2_. The MTT solution was then discarded, and 140 µL of DMSO (PharmaMed LLC, Russia) was added. The plates were swayed on a shaker (60 rpm) to dissolve the formazan. The absorbance was measured using a microplate reader (VICTOR ×5 Light Plate Reader, PerkinElmer, Waltham, MA, USA) at a wavelength of 565 nm (in order to measure formazan concentration). The results were used to construct a dose–response graph and to estimate IC_50_ value (concentration of compounds, which caused 50% death of cells) with GraphPadSoftware, Inc., San Diego, CA, USA.

### 3.8. Plate Test and Determination of Mechanism of Action

A total of 1.5 µL of 20 mM solution of each sample was applied to an agar plate containing a lawn of the *E. coli DTC, E. coli LPTD*, or the reporter strain (*E.coli. DTC*-pDualrep2 was used as previously described [44]). After overnight incubation at 37 °C, the plate was scanned by the ChemiDoc (Bio-Rad, Hercules, CA, USA) system with two channels, including “Cy3-blot” (553/574 nm, green pseudocolor) for RFP fluorescence and “Cy5-blot” (588/633 nm, red pseudocolor) for Katushka2S fluorescence.Cells without reporter construction could be detected in both channels. The induction of the expression of Katushka2S is triggered by translation inhibitors, while RFP is up-regulated by the induction of DNA damage SOS response. In addition, 2 µL of levofloxacin (25 μg/mL) and erythromycin (5 mg/mL) were used as positive controls for DNA biosynthesis and ribosome inhibitors, respectively.

### 3.9. Determination of Minimal Inhibitory Concentration

Minimal inhibitory concentration values were determined by monitoring growth in 96-well plates of E. coli cultures exposed to serial dilutions of compounds **5a**–**f**. Specifically, overnight E. coli LPTD cultures were diluted in 96-well plates to an OD600 of 0.01 in LB medium. The wells were then supplemented with antibiotic at concentrations of 200.00, 100.00, 50.00, 25.00, 12.50, 6.25, 3.13, 1.56, 0.78, and 0.39 µM. After antibiotic addition, the plates were incubated with shaking (200 r.p.m.) overnight at 37 °C, and cell growth was assessed by scanning each well with a VictorX5 reader.

## 4. Conclusions

In the present study, the first successful synthesis of dispirooxindole-β-lactam scaffold was demonstrated. It is also shown that the implementation of Staudinger ketene-imine cycloaddition allows the formation of the desired spirooxindoles with high diastereoselectivity and good yield. Meanwhile, an additional optimization of the synthetic procedure was necessary in order to improve the yield and lower the reaction time. Preliminary in vitro testing of obtained compounds demonstrated medium cytotoxicity of dispirooxindoles, but one of them shows antibacterial properties in the early micromolar range and has potential as an antibacterial agent, which provides data for further optimization of the structure. Therefore, this research offers important information for the development of bioactive compounds, including cytotoxic ones and antibacterials based on such a rigid **3d** scaffold as dispirooxindole-β-lactams.

## Figures and Tables

**Table 1:** Affinity energy comparison of dispirooxindole (**a**) and the previously described [27] spirooxindole-β-lactam (**b**).

Compound	Figure 2 Entry#	Stereoisomer	Affinity (kcal/mol)
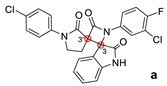	**a1**	*(3R,3′S)*	−10.1
	**a2**	*(3S,3′R)*	−9.1
	**a3**	*(3R,3′R)*	−8.8
	**a4**	*(3S,3′S)*	−8.1
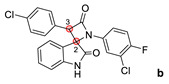	**b1**	*(2R,3S)*	−9.2
	**b2**	*(2S,3R)*	−9.4
	**b3**	*(2R,3R)*	−8.1
	**b4**	*(2S,3S)*	−7.3

**Table 2:** Selected bond lengths and angles for compound **5a**.

Bond	(Å)	Angle; Torsion	(°)
N(2)-C(23)	1.410(3)	C(11)-N(2)-C(3)	95.8(2)
N(2)-C(11)	1.348(4)	N(2)-C(11)-C(12)	93.2(2)
N(2)-C(3)	1.467(3)	N(2)-C(3)-C(12)	86.3(2)
C(3)-C(12)	1.596(4)	C(11)-C(12)-C(3)	83.8(2)
C(11)-C(12)	1.534(4)	C(13)-C(12)-C(15)	104.3(2)
O(2)-C(11)	1.202(3)	C(13)-C(12)-C(15)	104.3(2)
N(3)-C(16)	1.434(3)	C(14)-C(13)-C(12)	106.9(2)
N(3)-C(15)	1.342(3)	C(15)-N(3)-C(14)	112.7(2)
N(3)-C(14)	1.455(3)	N(3)-C(14)-C(13)	106.3(3)
C(12)-C(13)	1.518(4)	C(11)-N(2)-C(3)-C(10)	134.3(2)
C(12)-C(15)	1.519(3)	N(2)-C(11)-C(12)-C(13)	131.6(2)
C(13)-C(14)	1.478(4)	C(11)-N(2)-C(23)-C(28)	160.1(3)
O(3)-C(15)	1.224(3)	C(23)-N(2)-C(3)-C(2)	70.4(3)

**Table 3:** IC_50_ (μM) values and calculated LogP (octanol/water) for dispirooxindole-β-lactams **5a**–**f** and some previously synthesized [25,38] spirooxindole-β-lactams.

Compound	#	Ar^1^	Ar^2^	Mcf7	VA13	A549	Hek293t	LogP(o/w) *
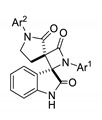	**5a**	4-OMe-Ph	4-OMe-Ph	104.3 ± 20.0	103.1 ± 2.0	66.0 ± 8.7	27.0 ± 3.6	3.12
	**5b**	4-OMe-Ph	4-Me-Ph	27.1 ± 2.1	23. 5 ± 7.2	17.4 ± 1.7	14.0 ± 0.9	3.46
	**5c**	4-OMe-Ph	4-Cl-Ph	5. 7 ± 1.1	~10	6.4 ± 0.4	~5	3.76
	**5d**	3-Me-Ph	3-Me-Ph	17.9 ± 3.0	12.5 ± 0.4	8.0 ± 1.10	8.8 ± 0.8.	3.88
	**5e**	3-Me-Ph	4-Cl-Ph	7.6 ± 0.7	7.7 ± 1.0	7.10 ± 0.5	6.5 ± 0.4	4.14
	**5f**	3-Me-Ph	4-F,3-Cl-Ph	5.6 ± 0.6	7.3 ± 1.1	6.9 ± 0.4	6.1 ± 0.4	4.32
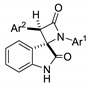	***cis* [27]**	4-Cl-Ph	4-F,3-Cl-Ph	14.4 ± 1.9	16.2 ± 2.2	8.6 ± 0.6	12.7 ± 0.7	4.85
	***cis* [38]**	4-Cl-Ph	4-OMe-Ph	41.9 ± 3.4	25.0 ± 1.9	28.4 ± 11.3	27.6 ± 4.1	4.30
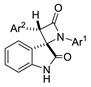	***trans* [27]**	4-Cl-Ph	4-F,3-Cl-Ph	18.4 ± 2.1	16.6 ± 2.9	9.6 ± 0.6	17.8 ± 4.1	4.85

* LogP (o/w) was calculated with MOE2014 software (CCG company).

## Data Availability

CCDC-2171648 (compound **5a**) and CCDC-2172682 (compound **6**) contain the supplementary crystallographic data for this paper. The data can be obtained free of charge from The Cambridge Crystallographic Data Centre via www.ccdc.cam.ac.uk/data_request/cif website.

## Supplementary Materials

The following supporting information can be downloaded at: https://www.mdpi.com/article/10.3390/ijms23126666/s1: NMR spectra of synthesized compounds, X-ray data.
