# Supplementary material for: Dispirooxindole-β-Lactams: Synthesis via Staudinger Ketene-Imine Cycloaddition and Biological Evaluation
Source: Int J Mol Sci. 2022 Jun 15;23(12):6666. doi: 10.3390/ijms23126666 (PMC9223813; doi:10.3390/ijms23126666)
Supplement: Supplementary file 1 [file ijms-23-06666-s001.zip › ijms-1752679-supplementary.pdf]

Supporting Information

Dispirooxindole-β-lactams: Synthesis via Staudinger ketene-imine cycloaddition and biological evaluation

Vadim E. Filatov [1], Dmitrii A. Iuzabchuk [1], Viktor A. Tafeenko [1], Yuri K. Grishin [1], Vitaly A. Roznyatovsky, Dmitry A. Skvortsov, Ilya A. Osterman, Nikolai V. Zyk [1], Elena K. Beloglazkina*

Department of Chemistry, Lomonosov Moscow State University,119234 Moscow, Russian Federation

Table of contents

Spectral data 3

1-(4-methoxyphenyl)-2-oxopyrrolidine-3-carboxylic acid (3a). 3

2-oxo-1-(p-tolyl)pyrrolidine-3-carboxylic acid (3b) 5

2-oxo-1-(m-tolyl)pyrrolidine-3-carboxylic acid (3c) 7

1-(4-chlorophenyl)-2-oxopyrrolidine-3-carboxylic acid (3d) 9

1-(3-chloro-4-fluorophenyl)-2-oxopyrrolidine-3-carboxylic acid (3e) 11

(3S*,3'S*)-1',1''-bis(4-methoxyphenyl)dispiro[indoline-3,2'-azetidine-3',3''-pyrrolidine]-2,2'',4'-trione (5a) 13

(3S,3'S)-1',1''-bis(4-methoxyphenyl)-1-tosyldispiro[indoline-3,2'-azetidine-3',3''-pyrrolidine]-2,2'',4'-trione (6) 18

N,1-bis(4-methoxyphenyl)-2-oxopyrrolidine-3-carboxamide (7) 19

(3S,3'S)-1'-(4-methoxyphenyl)-1''-(p-tolyl)dispiro[indoline-3,2'-azetidine-3',3''-pyrrolidine]-2,2'',4'-trione (5b) 20

(3S,3'S)-1''-(4-chlorophenyl)-1'-(4-methoxyphenyl)dispiro[indoline-3,2'-azetidine-3',3''-pyrrolidine]-2,2'',4'-trione (5c) 22

(3S,3'S)-1',1''-di-m-tolyldispiro[indoline-3,2'-azetidine-3',3''-pyrrolidine]-2,2'',4'-trione (5d) 24

(3S,3'S)-1''-(4-chlorophenyl)-1'-(m-tolyl)dispiro[indoline-3,2'-azetidine-3',3''-pyrrolidine]-2,2'',4'-trione (5e) 26

(3S,3'S)-1''-(3-chloro-4-fluorophenyl)-1'-(m-tolyl)dispiro[indoline-3,2'-azetidine-3',3''-pyrrolidine]-2,2'',4'-trione (5f) 28

X-Ray 30

Spectral data

*1-(4-methoxyphenyl)-2-oxopyrrolidine-3-carboxylic acid (**3a**).*

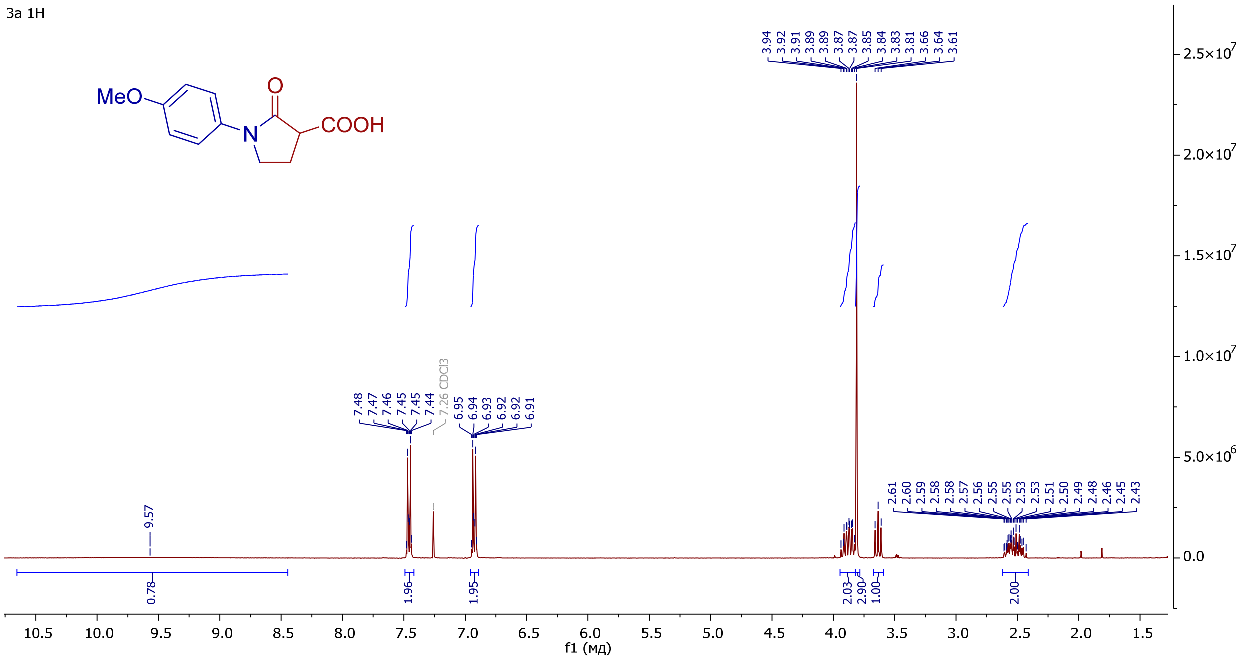

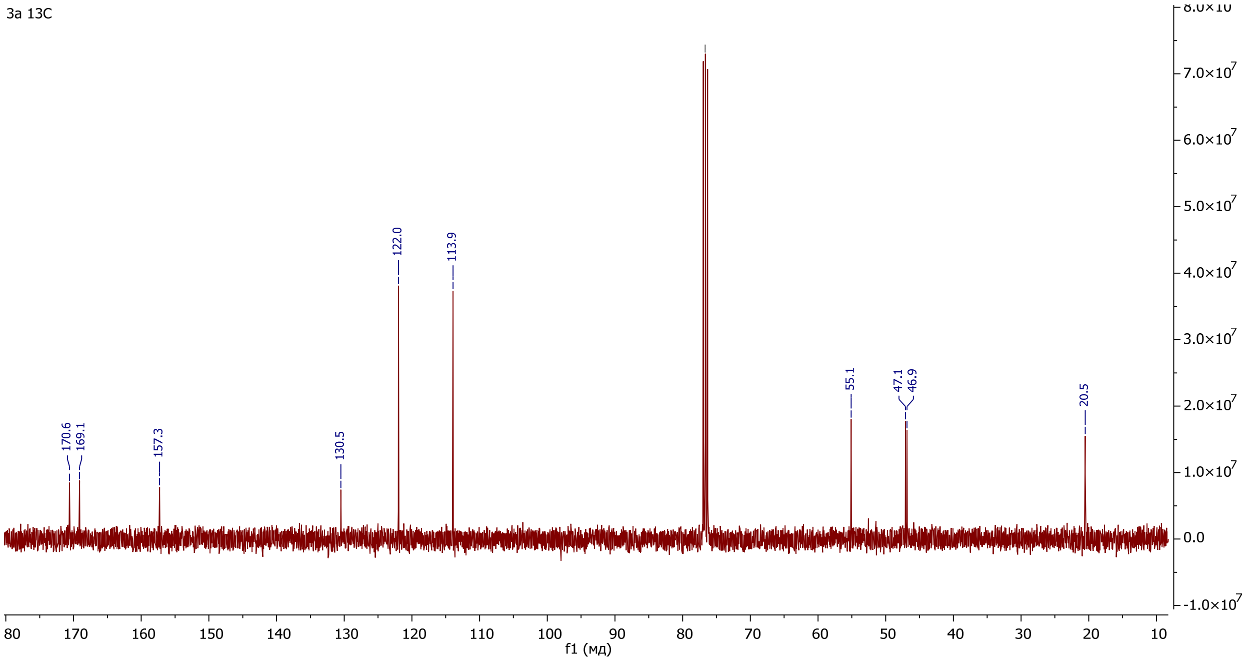

Figure S1

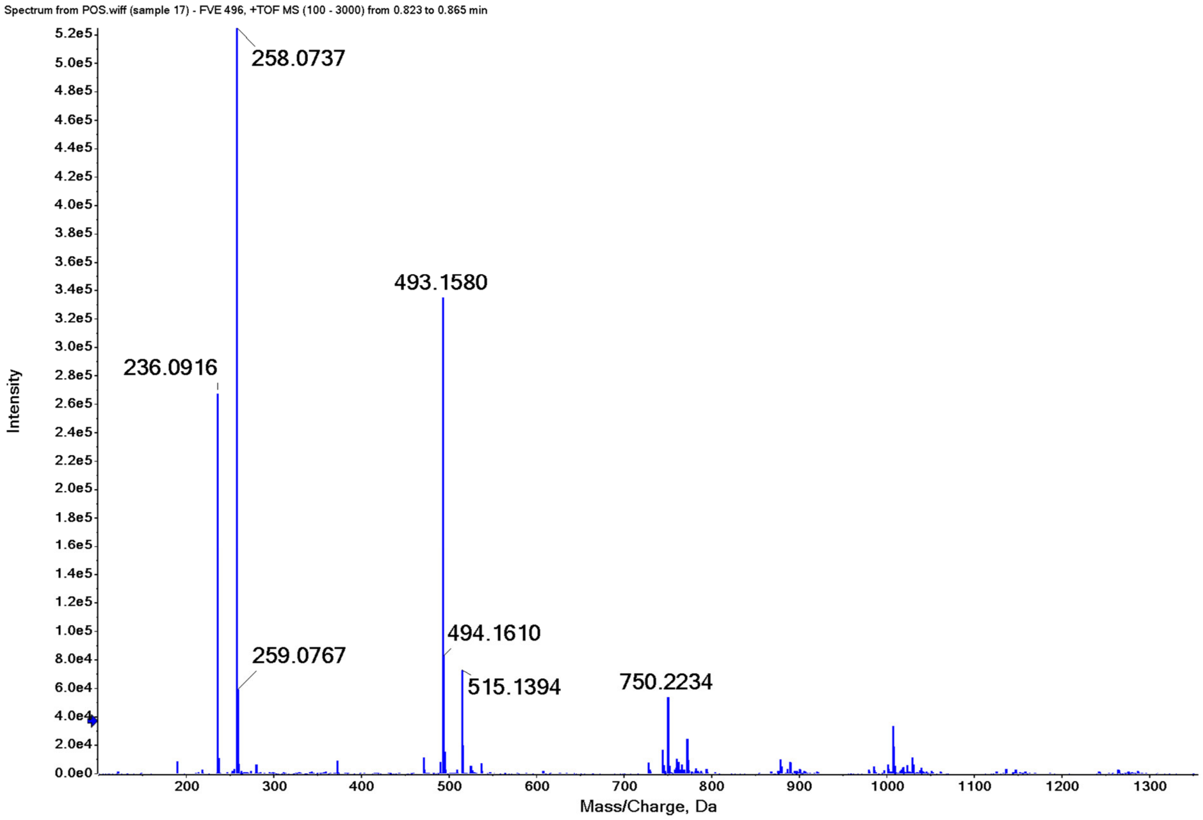
Spectrum from POS.wiff (sample 17) - FVE 496, +TOF MS (100 - 3000) from 0.823 to 0.865 min
258.0737
493.1580
236.0916
494.1610
259.0767
515.1394
750.2234
Intensity
Mass/Charge, Da

Figure S2

*2-oxo-1-(p-tolyl)pyrrolidine-3-carboxylic acid (**3b**)*

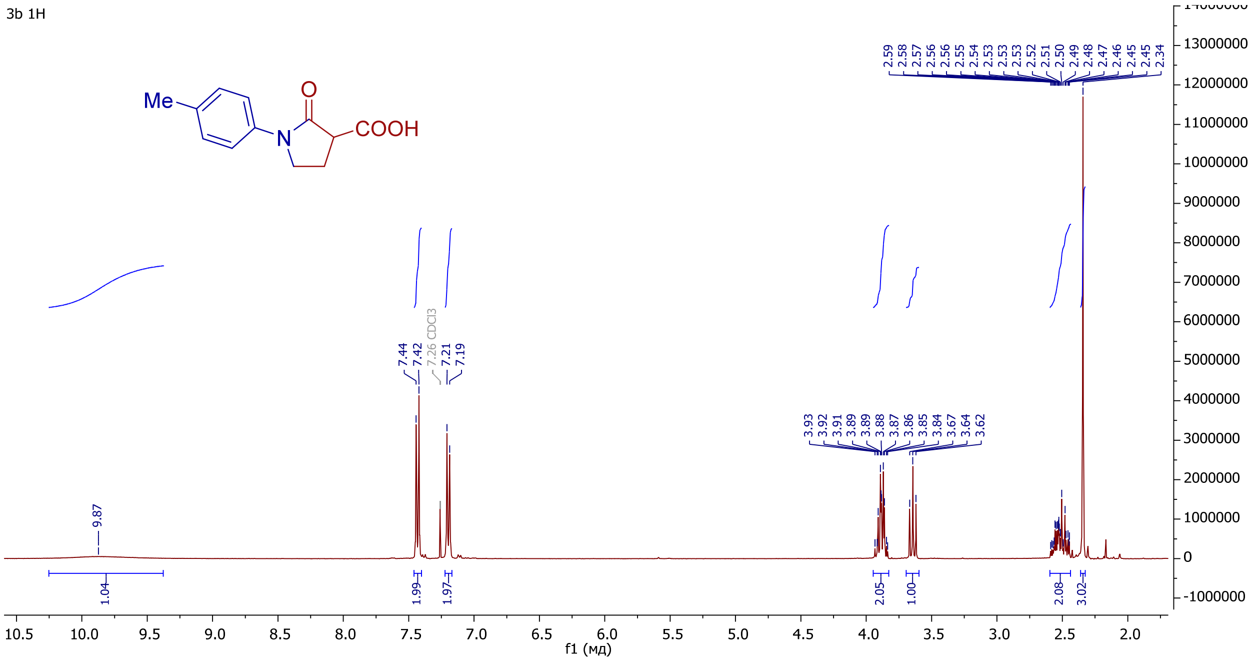

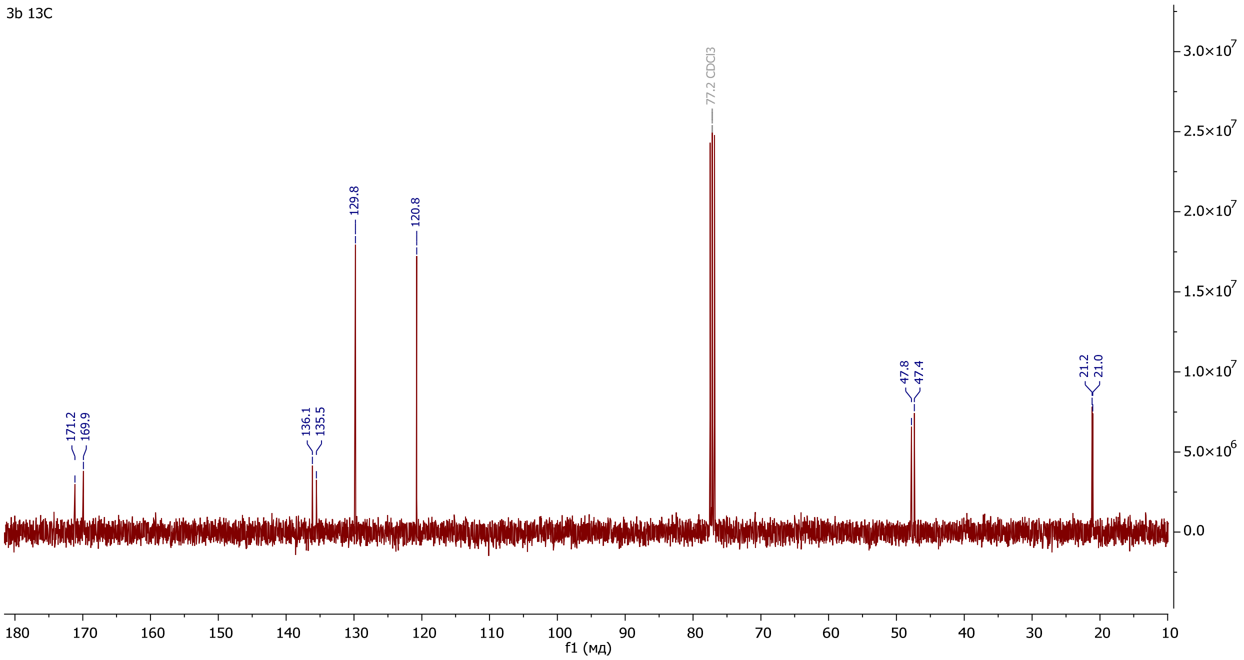

Figure S3

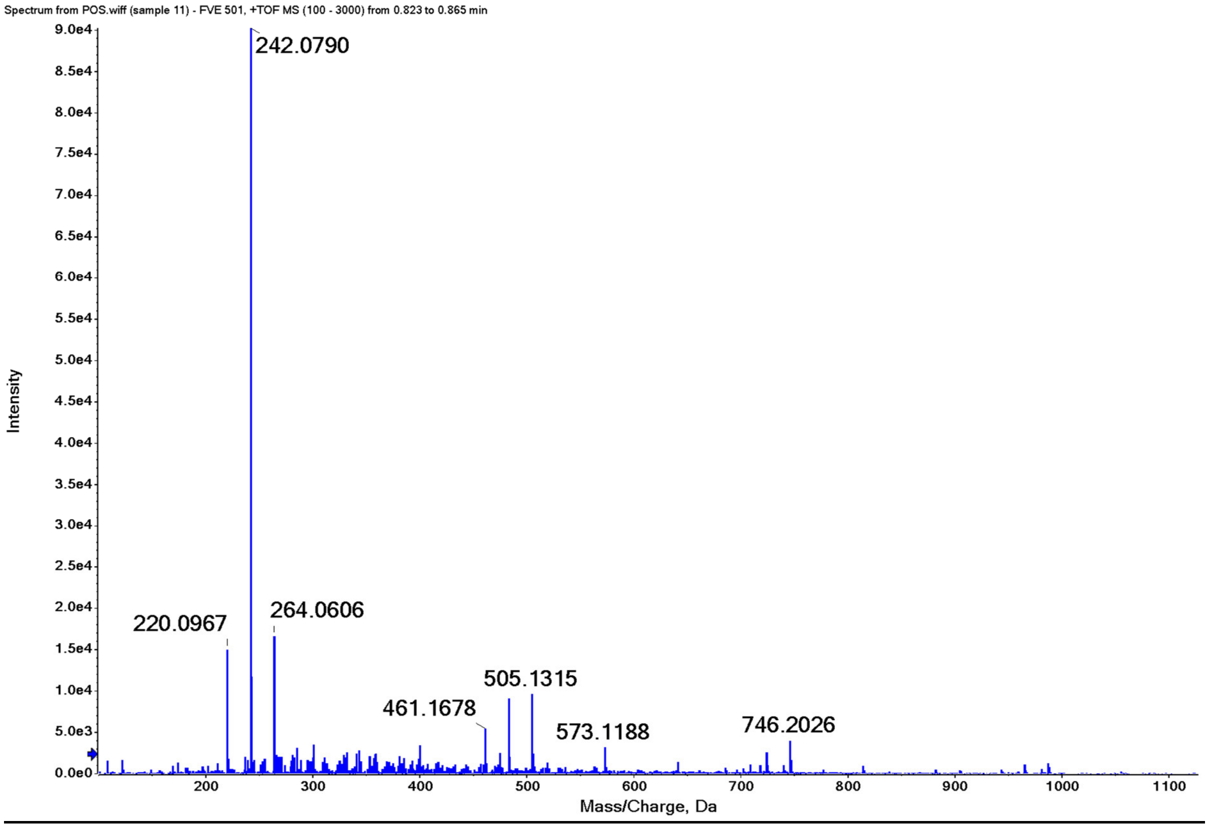
Spectrum from POS.wiff (sample 11) - FVE 501, +TOF MS (100 - 3000) from 0.823 to 0.865 min
Intensity
Mass/Charge, Da
242.0790
220.0967
264.0606
505.1315
461.1678
573.1188
746.2026

Figure S4

*2-oxo-1-(m-tolyl)pyrrolidine-3-carboxylic acid (**3c**)*

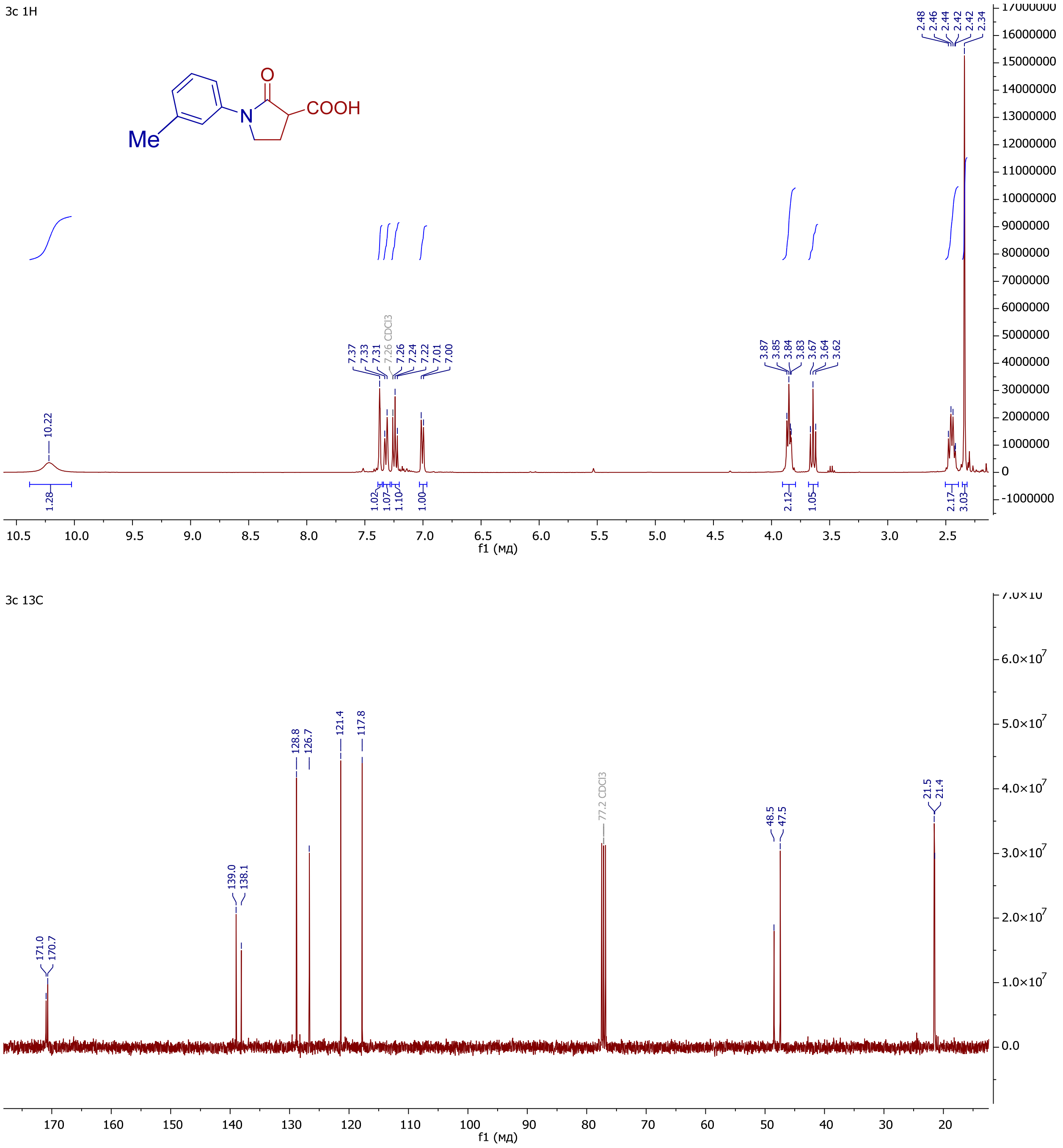

Figure S5

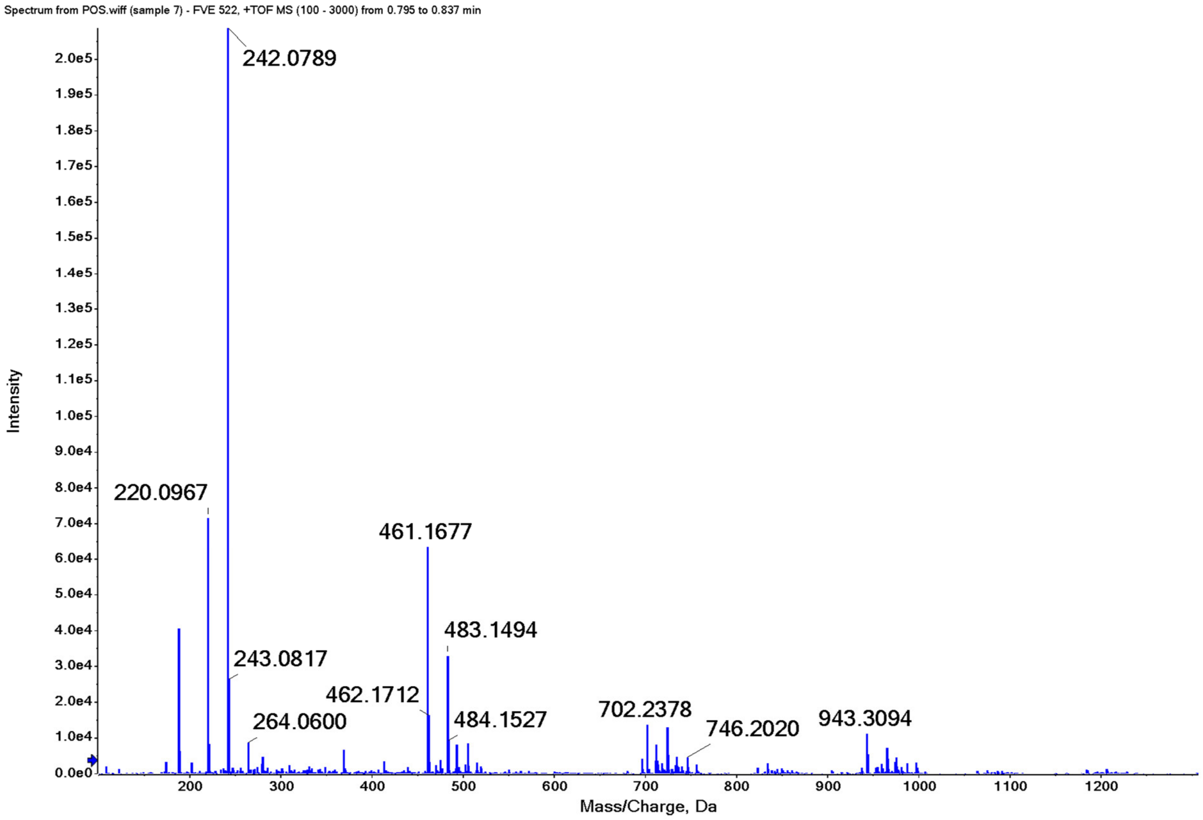

Spectrum from POS.wiff (sample 7) - FVE 522, +TOF MS (100 - 3000) from 0.795 to 0.837 min
Intensity
Mass/Charge, Da
242.0789
220.0967
461.1677
483.1494
243.0817
462.1712
264.0600
484.1527
702.2378
746.2020
943.3094

Figure S6

*1-(4-chlorophenyl)-2-oxopyrrolidine-3-carboxylic acid (**3d**)*

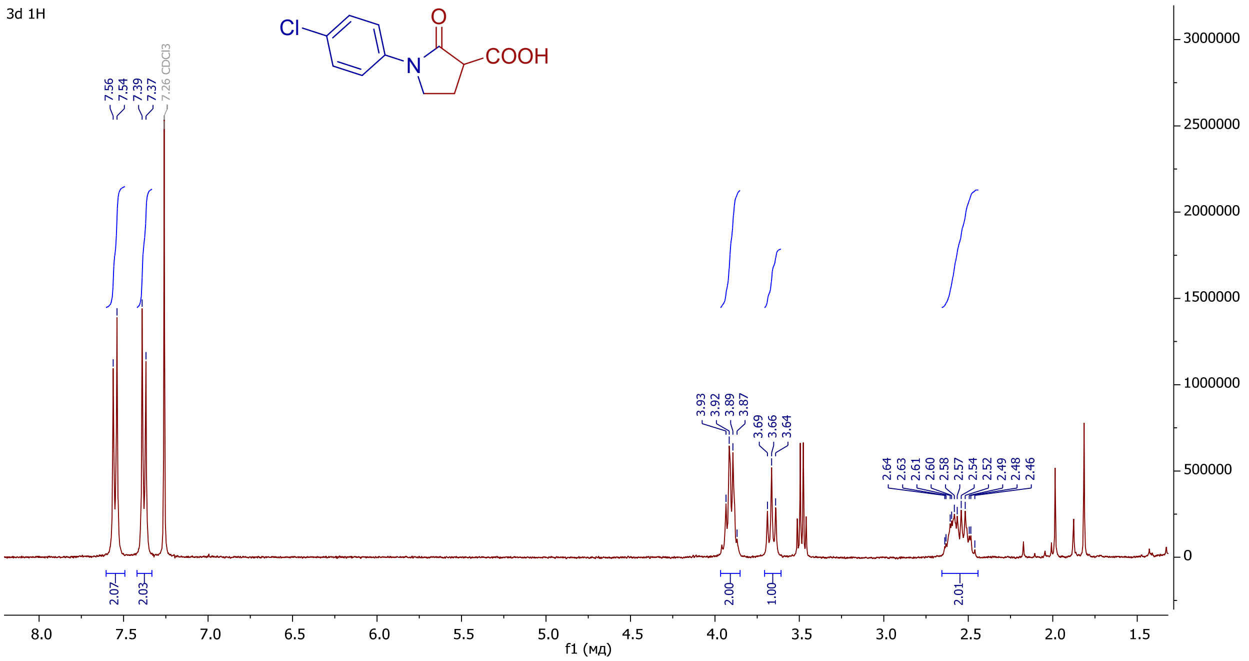

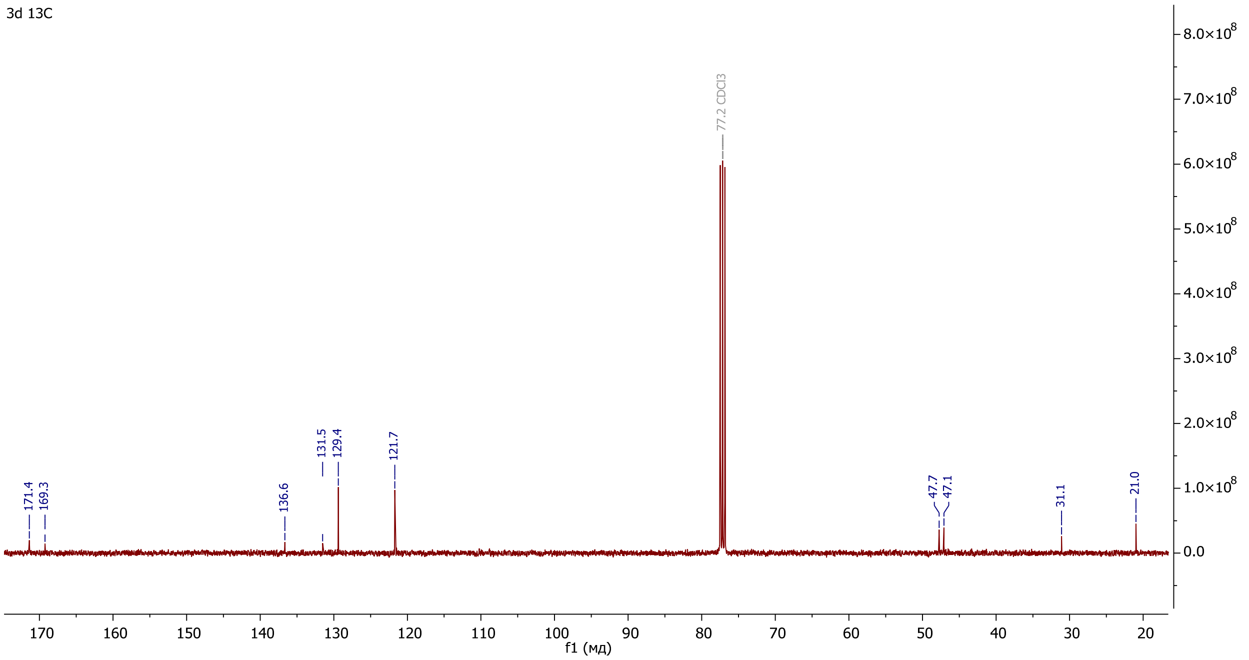

Figure S7

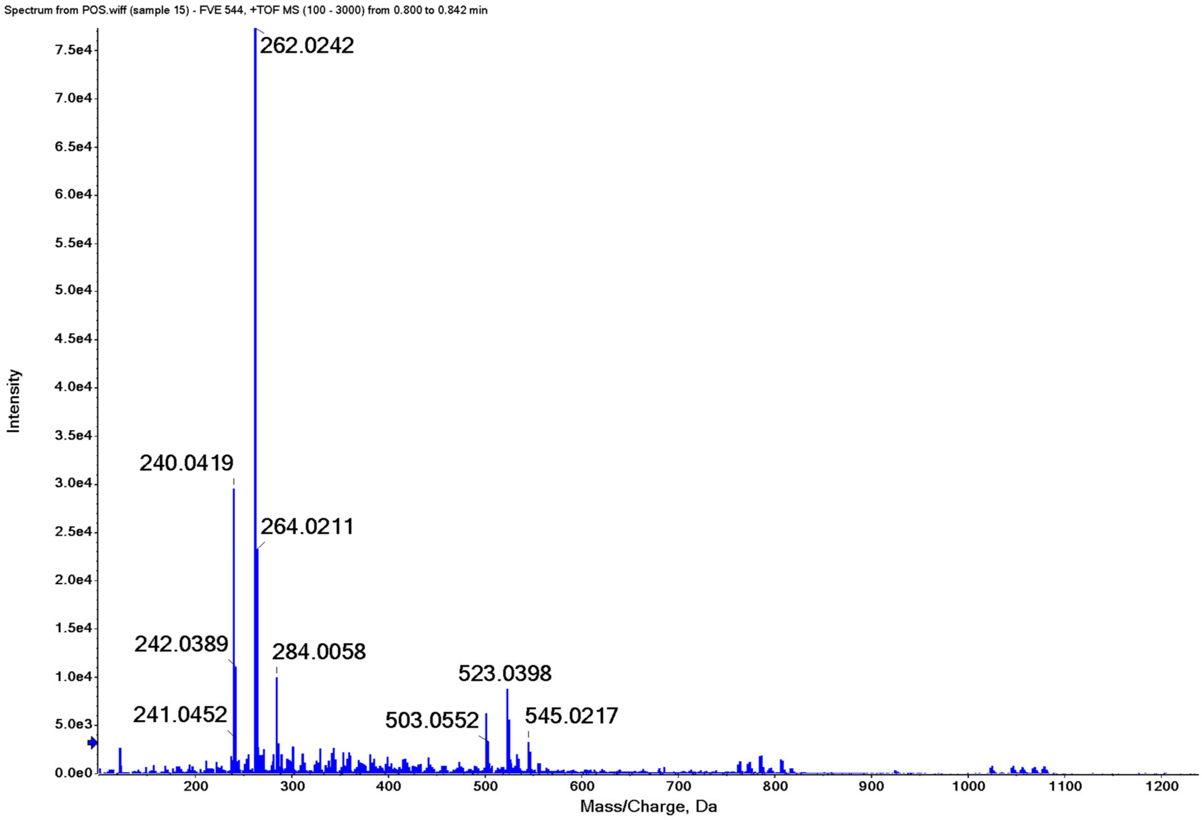
Spectrum from POS.wiff (sample 15) - FVE 544, +TOF MS (100 - 3000) from 0.800 to 0.842 min
262.0242
240.0419
264.0211
242.0389
284.0058
523.0398
241.0452
503.0552
545.0217
Intensity
Mass/Charge, Da

Figure S8

*1-(3-chloro-4-fluorophenyl)-2-oxopyrrolidine-3-carboxylic acid (**3e**)*

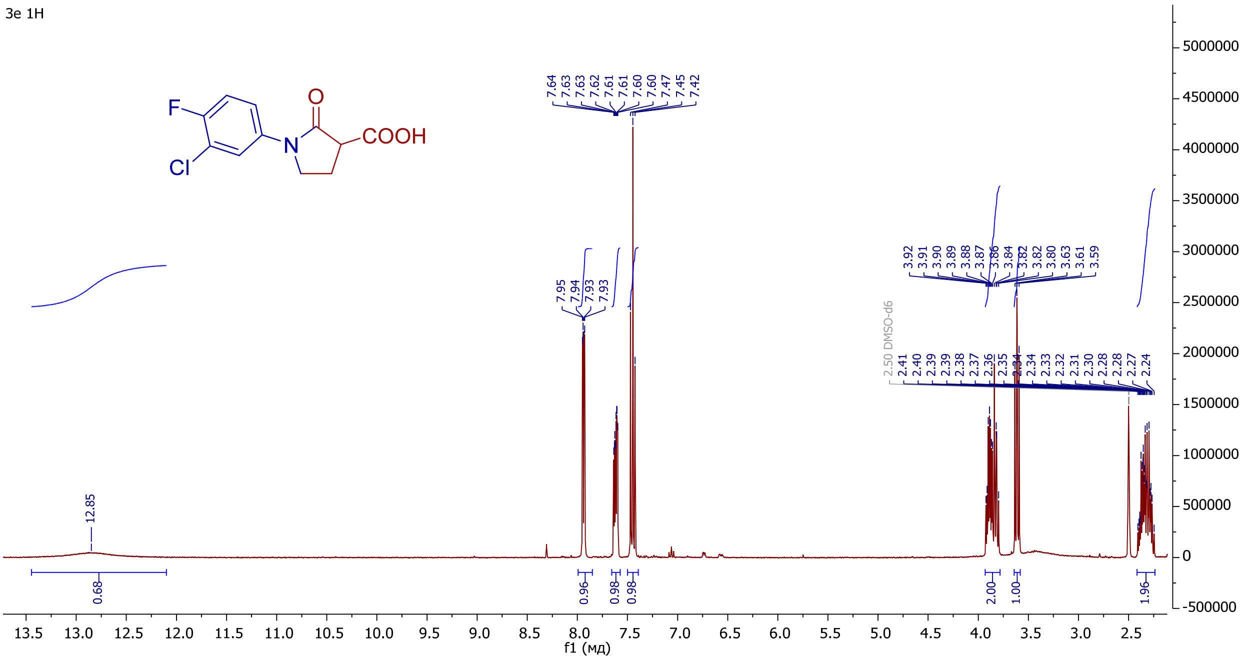

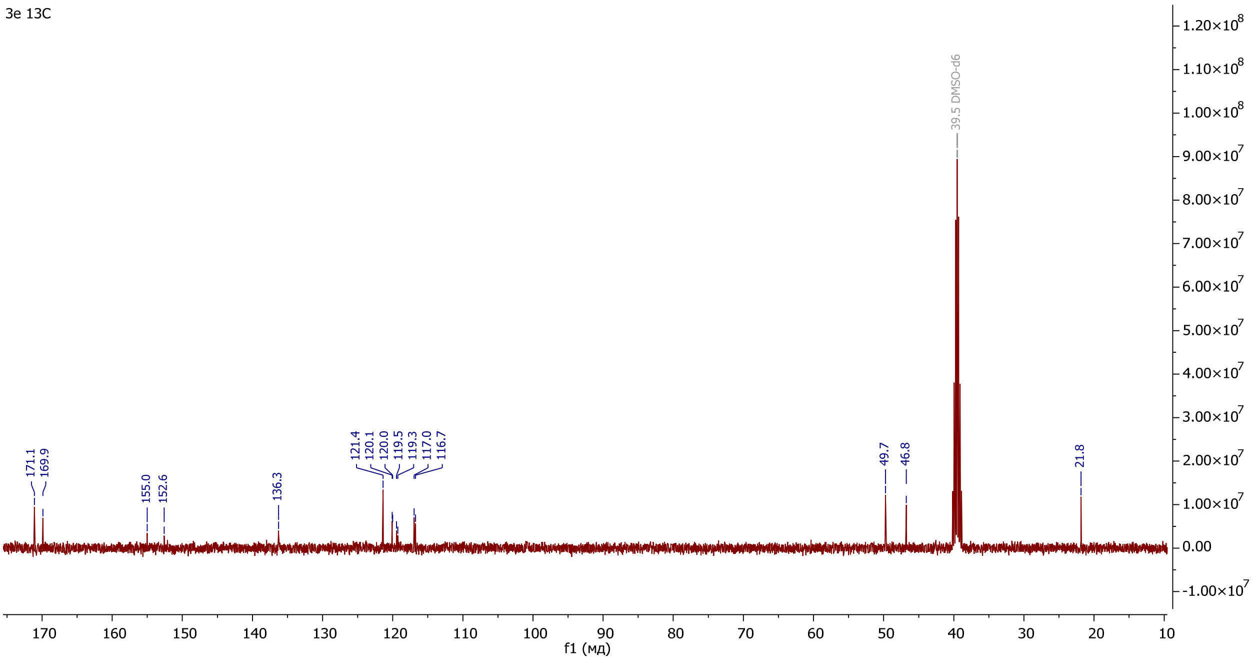

Figure S9

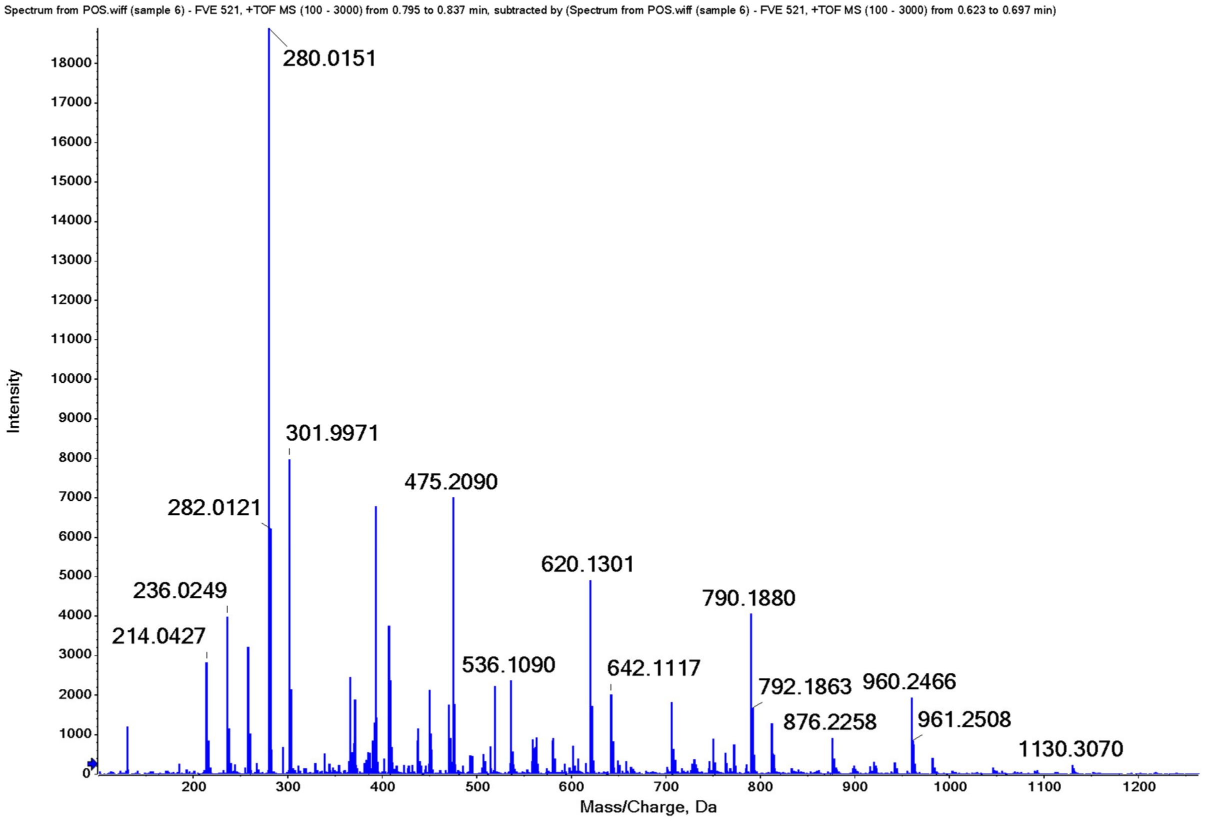
Spectrum from POS.wiff (sample 6) - FVE 521, +TOF MS (100 - 3000) from 0.795 to 0.837 min, subtracted by (Spectrum from POS.wiff (sample 6) - FVE 521, +TOF MS (100 - 3000) from 0.623 to 0.697 min)
Intensity
Mass/Charge, Da
280.0151
301.9971
282.0121
475.2090
620.1301
236.0249
214.0427
790.1880
536.1090
642.1117
792.1863
960.2466
876.2258
961.2508
1130.3070

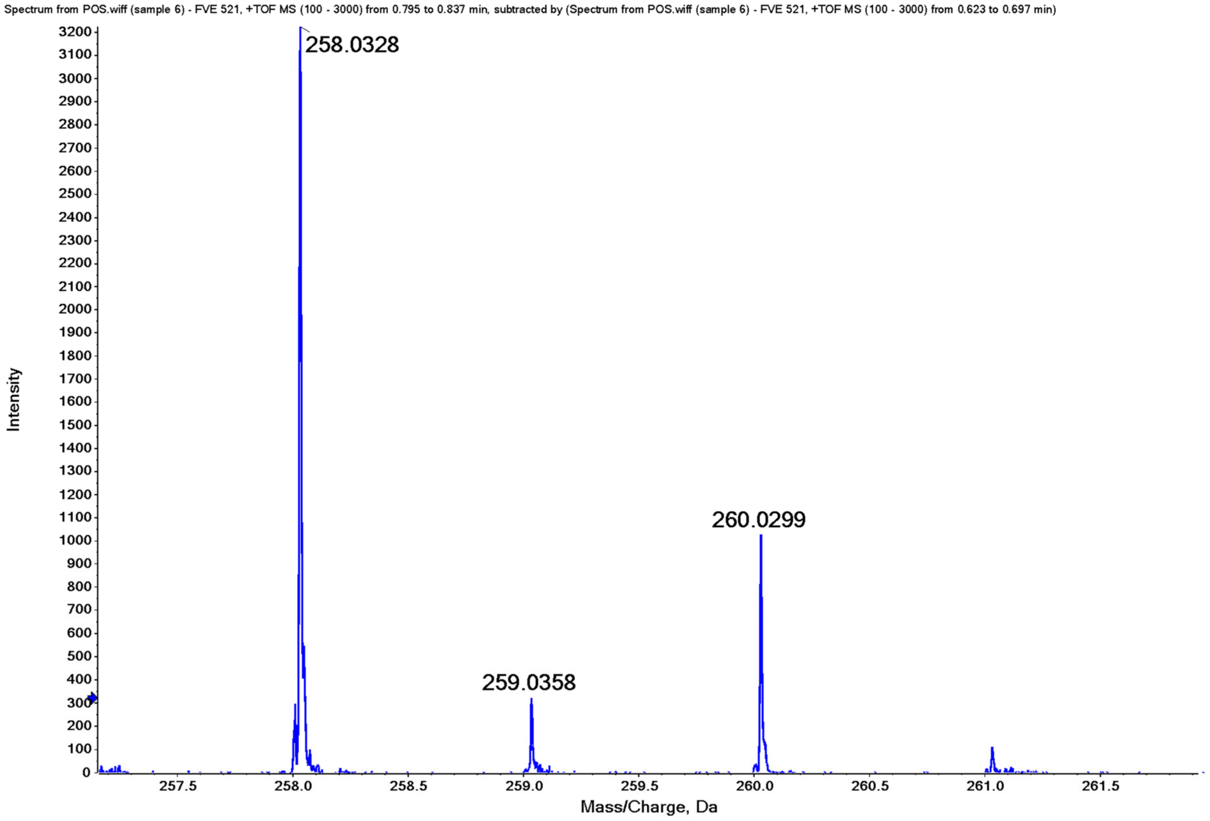
Spectrum from POS.wiff (sample 6) - FVE 521, +TOF MS (100 - 3000) from 0.795 to 0.837 min, subtracted by (Spectrum from POS.wiff (sample 6) - FVE 521, +TOF MS (100 - 3000) from 0.623 to 0.697 min)
Intensity
Mass/Charge, Da
258.0328
260.0299
259.0358

Figure S10

(3S,3′S*)-1′,1′′-bis(4-methoxyphenyl)dispiro[indoline-3,2′-azetidine-3′,3′′-pyrrolidine]-2,2′′,4′-trione* (***5a***)

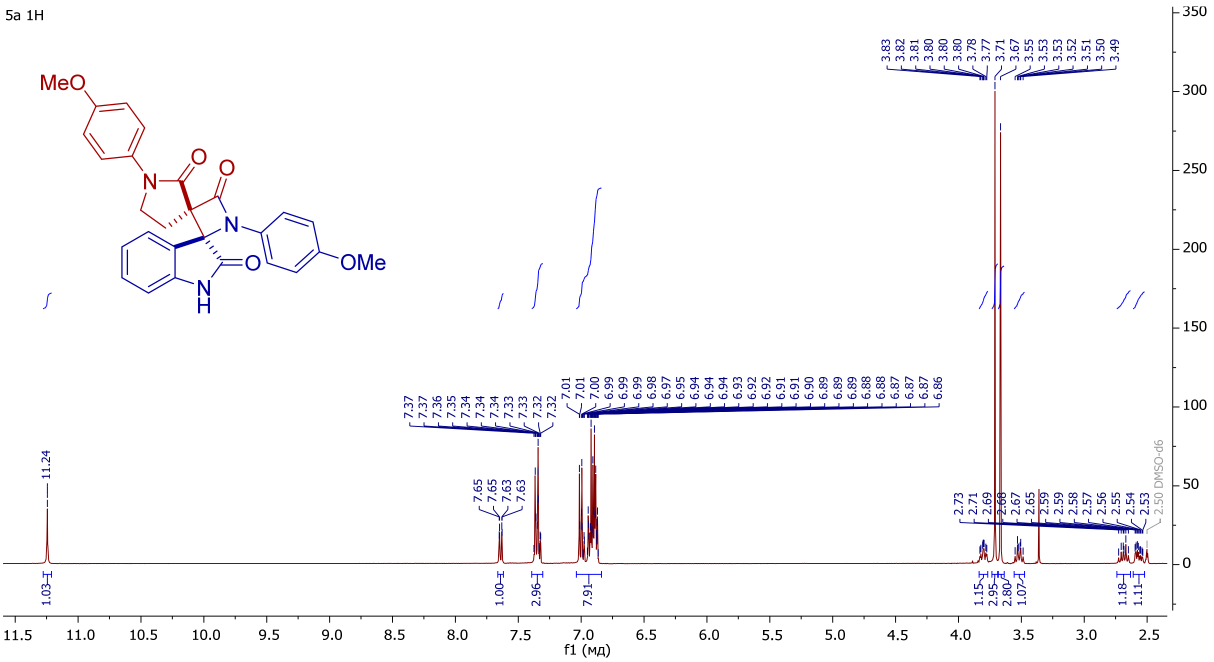

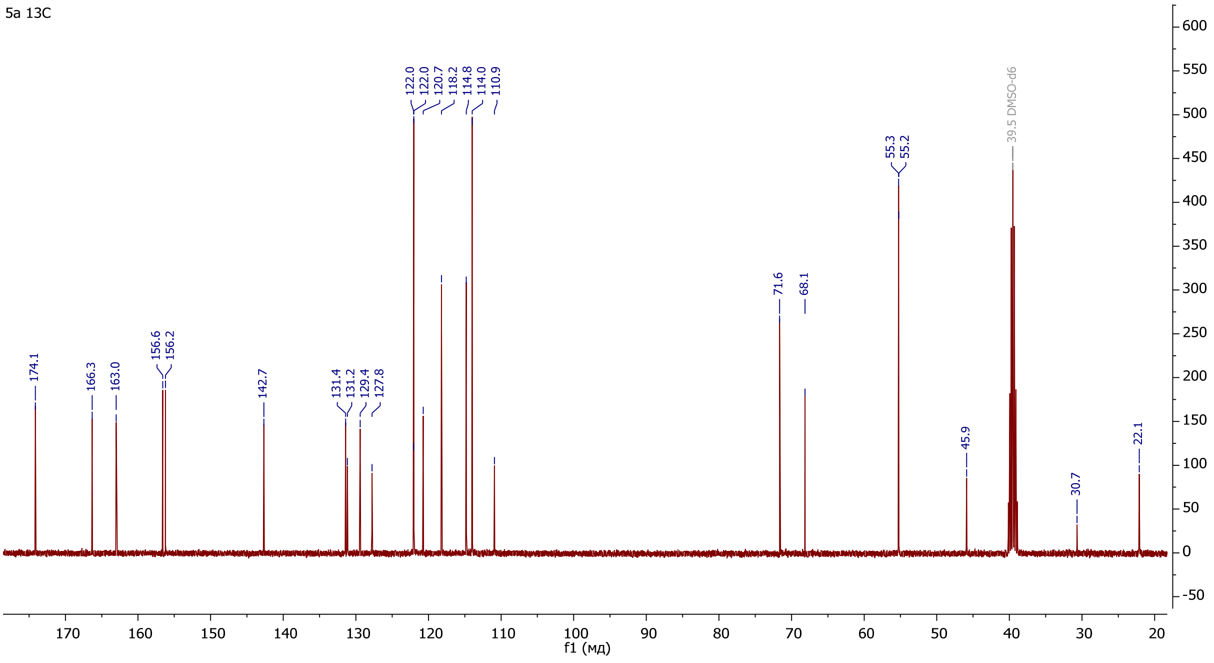

Figure S11

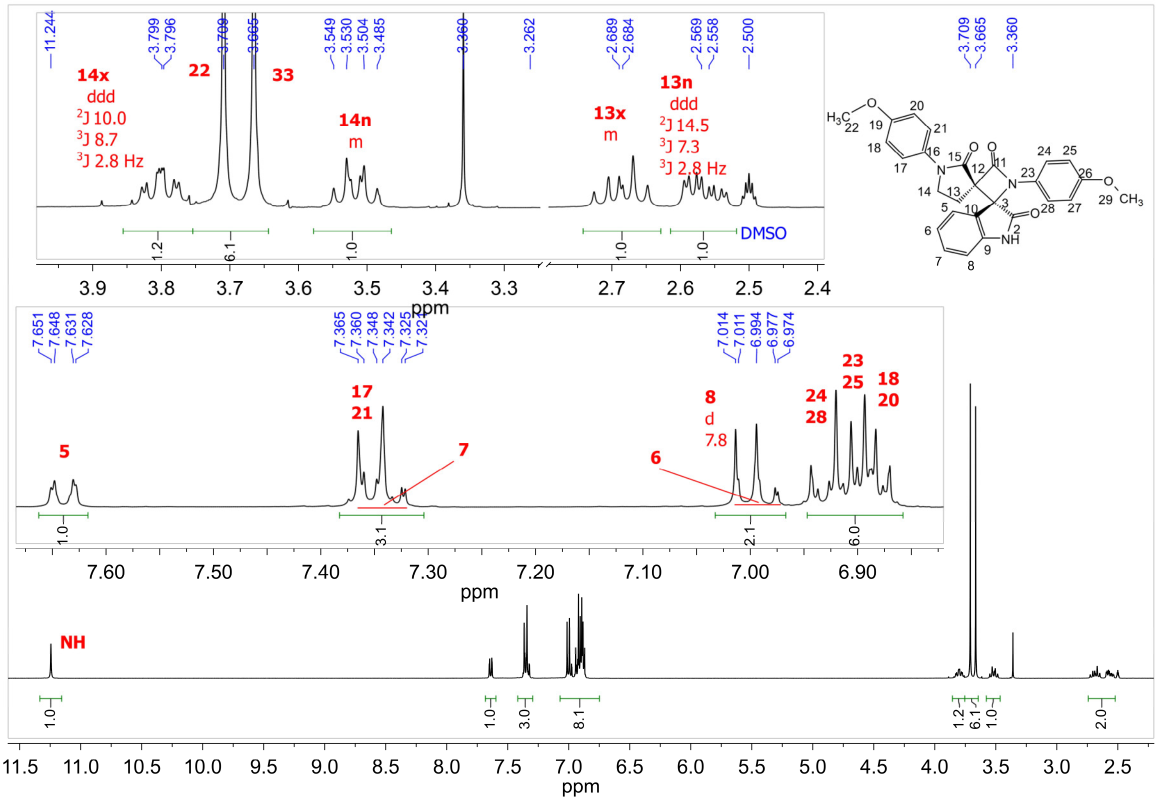
11.244
3.799
3.796
3.709
3.665
3.549
3.530
3.504
3.485
3.360
3.262
2.689
2.684
2.569
2.558
2.500
14x
ddd
^{2}J 10.0
^{3}J 8.7
^{3}J 2.8 Hz
22
33
14n
m
13x
m
13n
ddd
^{2}J 14.5
^{3}J 7.3
^{3}J 2.8 Hz
DMSO
1.2
6.1
1.0
1.0
1.0
ppm
7.651
7.648
7.631
7.628
7.365
7.360
7.348
7.342
7.325
7.324
7.014
7.011
6.994
6.977
6.974
5
17
21
7
6
8
d
7.8
24
28
23
25
18
20
1.0
3.1
2.1
6.0
ppm
NH
1.0
1.0
3.0
8.1
1.2
6.1
1.0
2.0
ppm

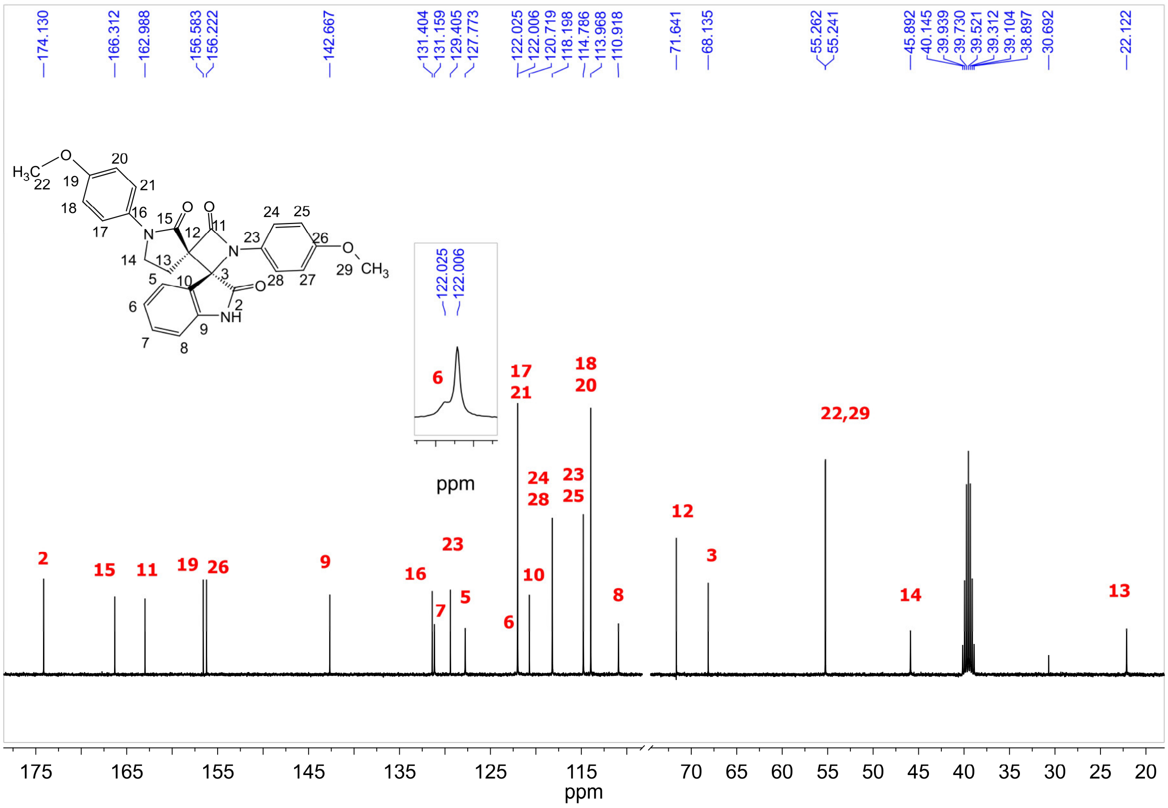
174.130
166.312
162.988
156.583
156.222
142.667
131.404
131.159
129.405
127.773
122.025
122.006
120.719
118.198
114.786
113.968
110.918
71.641
68.135
55.262
55.241
45.892
40.145
39.939
39.730
39.521
39.312
39.104
38.897
30.692
22.122
6
ppm
2
15
11
19
26
9
16
7
23
5
6
10
17
21
24
28
23
25
18
20
8
12
3
22,29
14
13
ppm

Figure S12

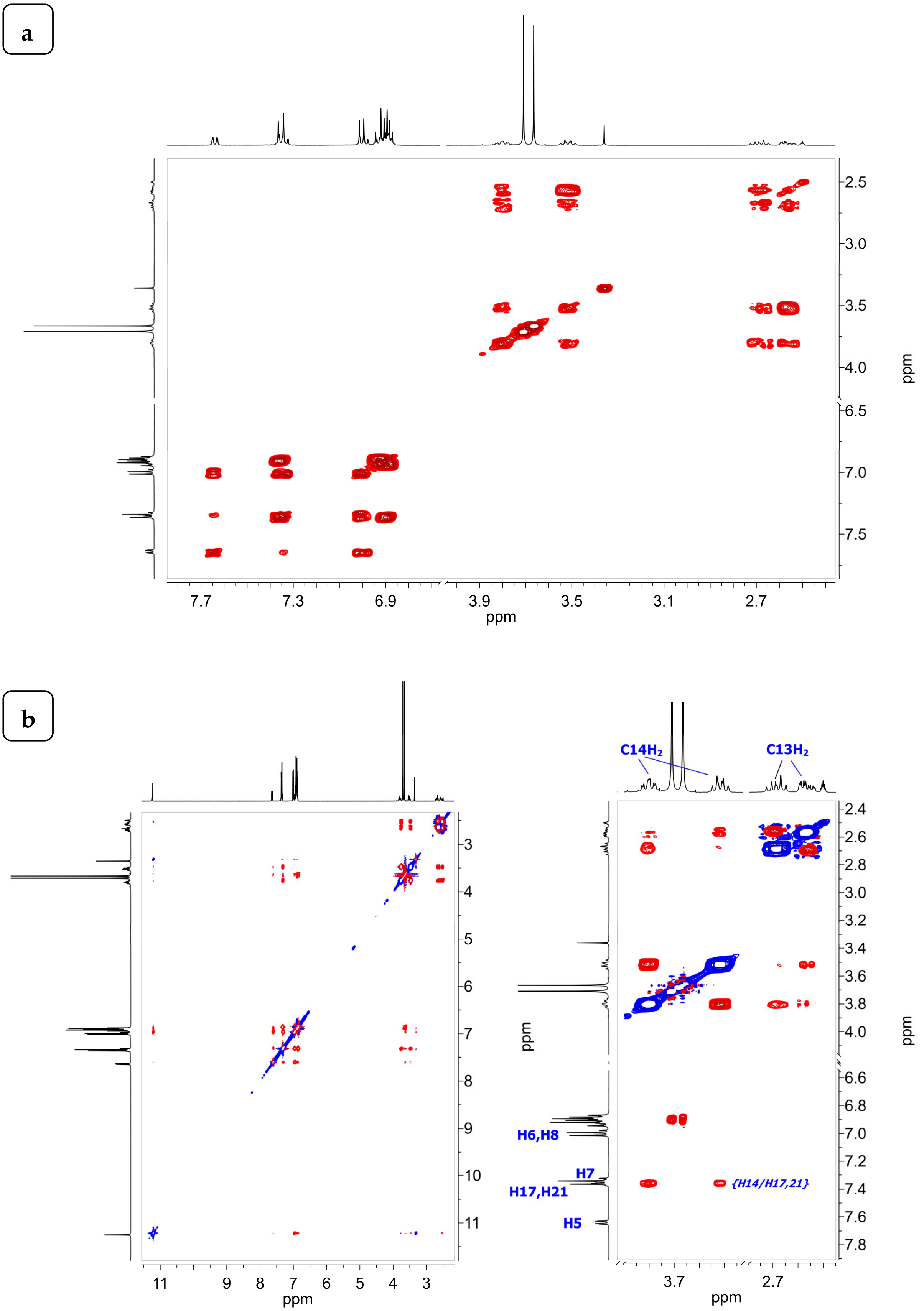

Figure S13: (a) gCOSY; (b)ROESYAD)

a

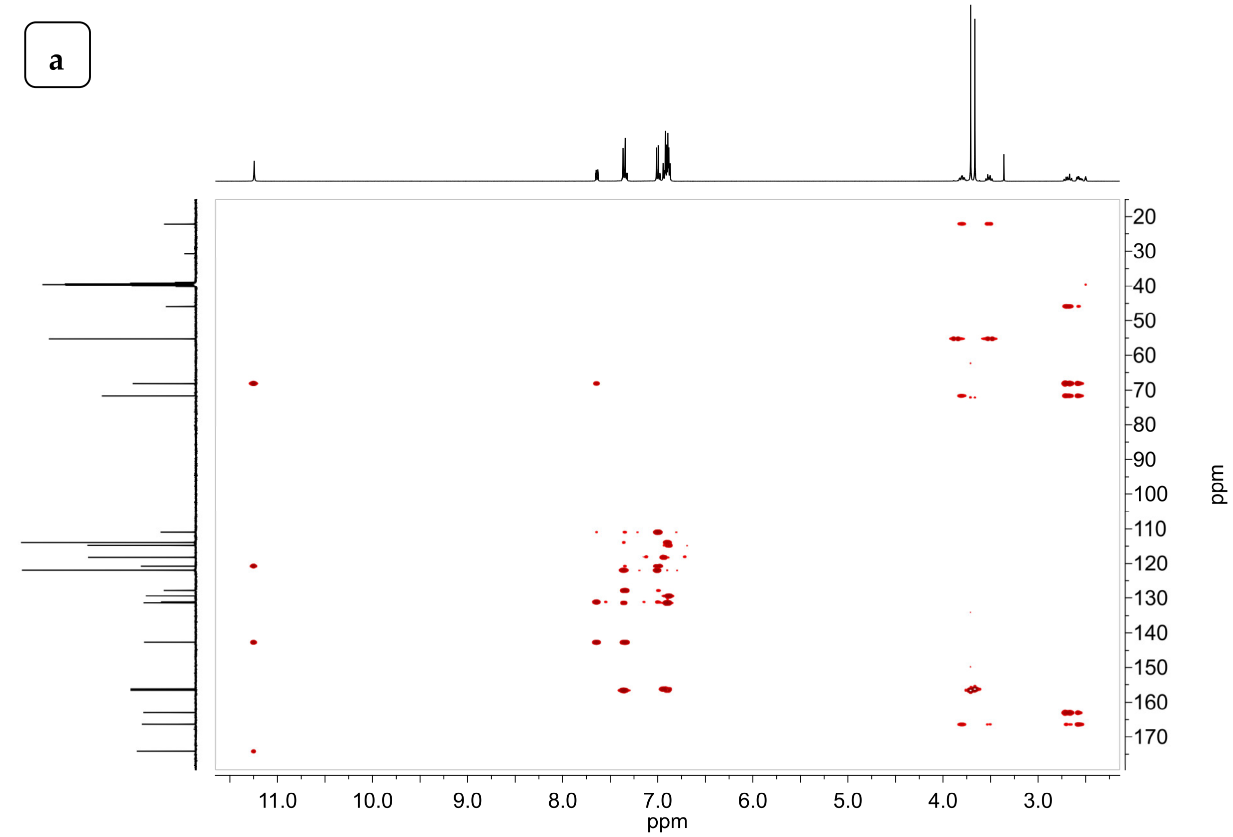

b

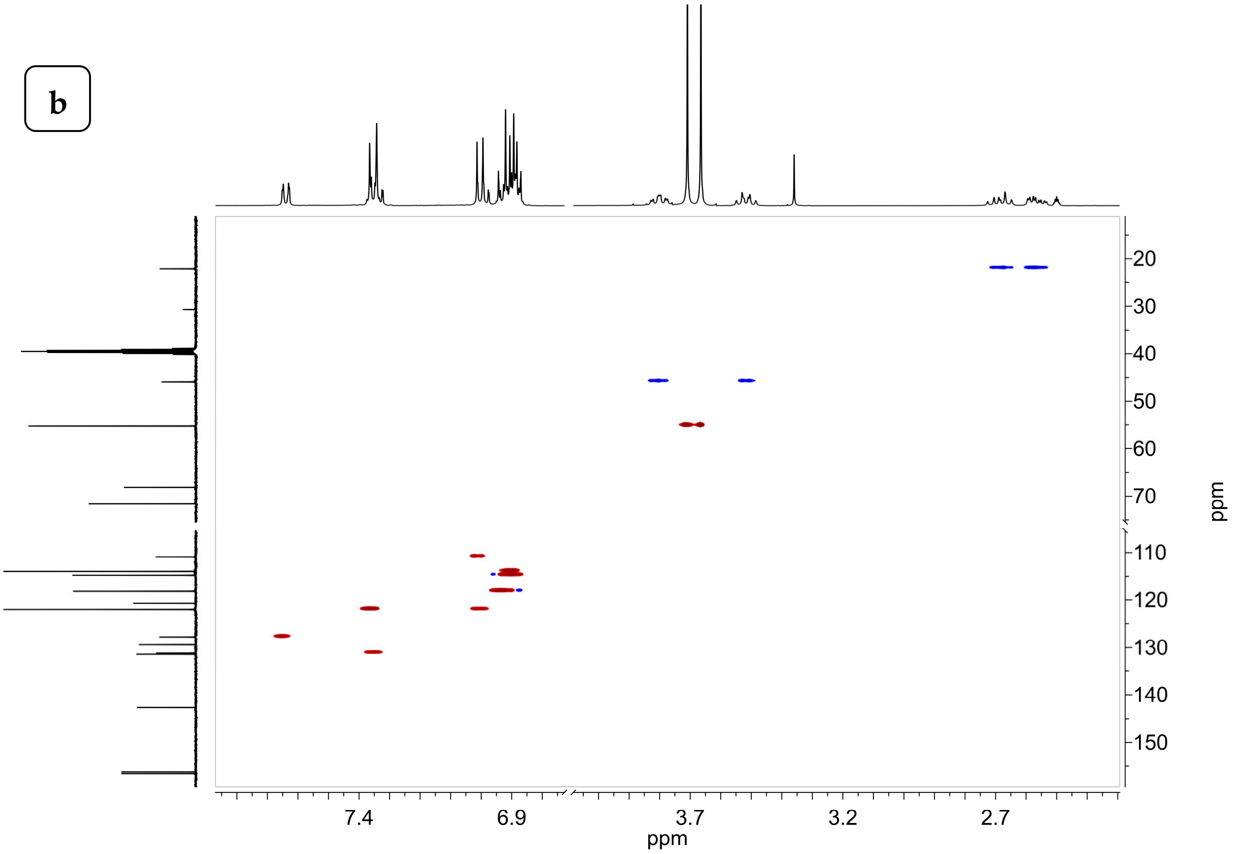

Figure S14: (a) gHSQCAD; (b)gHMBCAD

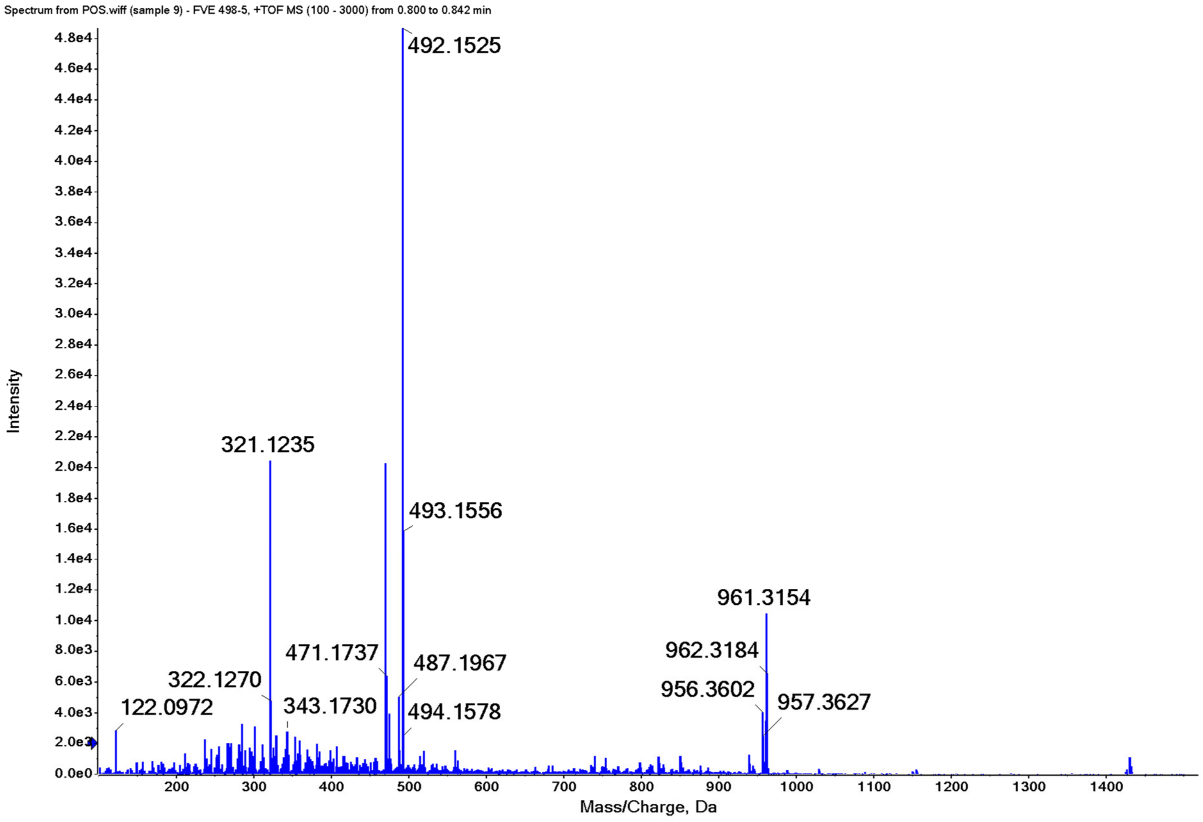

Spectrum from POS.wiff (sample 9) - FVE 498-5, +TOF MS (100 - 3000) from 0.800 to 0.842 min
Intensity
Mass/Charge, Da
492.1525
321.1235
493.1556
961.3154
962.3184
471.1737
487.1967
322.1270
956.3602
122.0972
343.1730
957.3627
494.1578

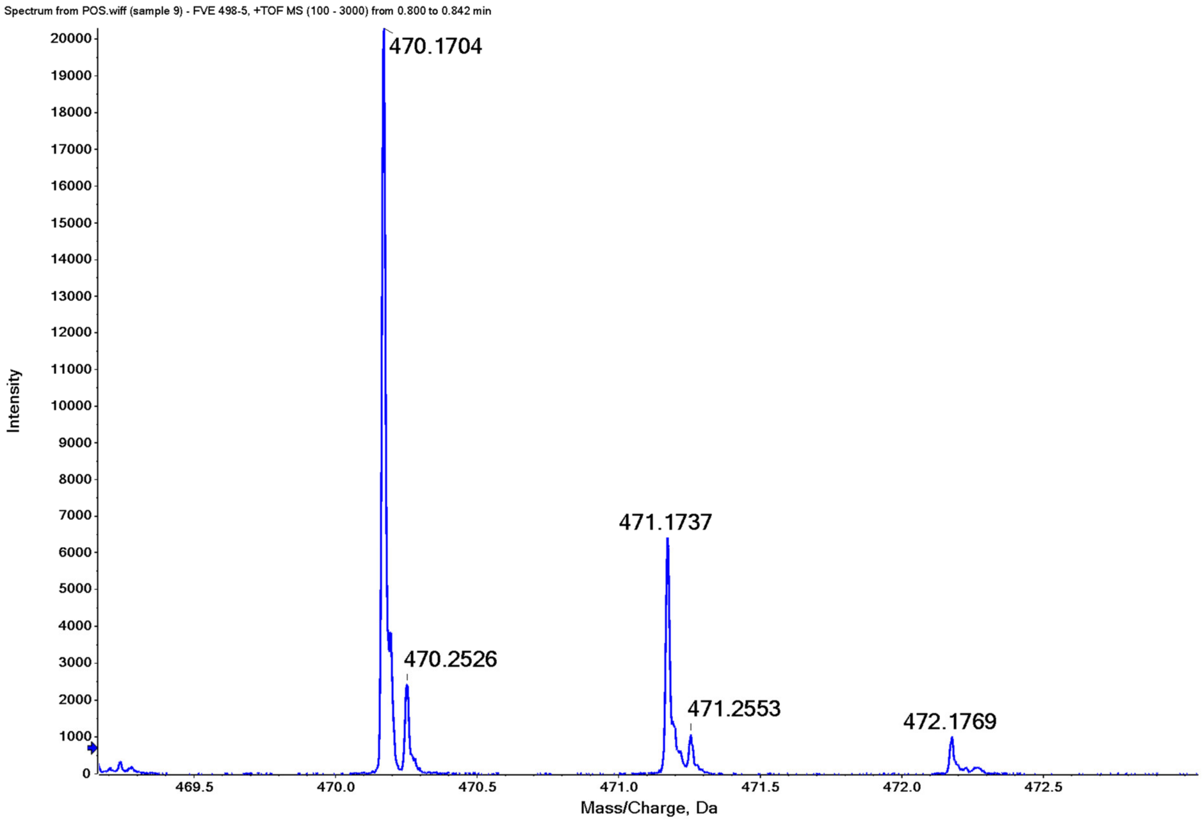

Spectrum from POS.wiff (sample 9) - FVE 498-5, +TOF MS (100 - 3000) from 0.800 to 0.842 min
Intensity
Mass/Charge, Da
470.1704
471.1737
470.2526
471.2553
472.1769

Figure S15

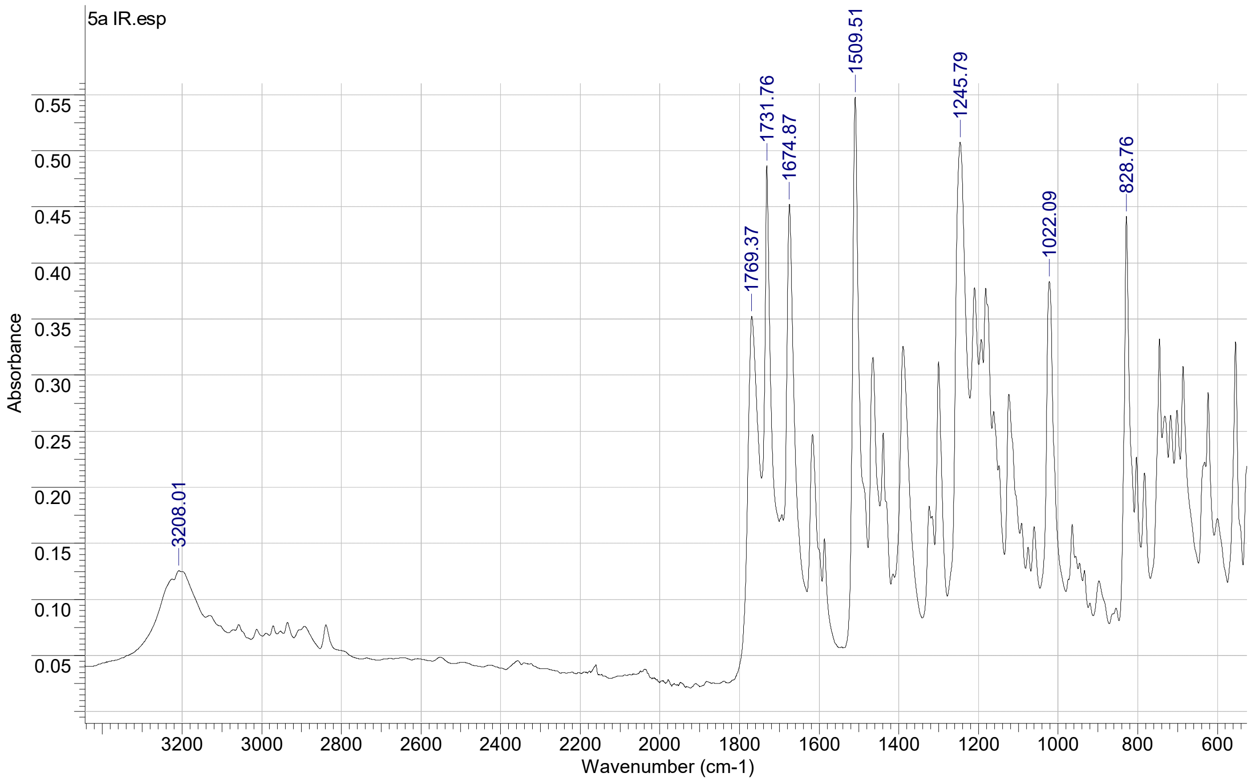
5a IR.esp
Absorbance
Wavenumber (cm-1)
3208.01
1769.37
1731.76
1674.87
1509.51
1245.79
1022.09
828.76
0.55
0.50
0.45
0.40
0.35
0.30
0.25
0.20
0.15
0.10
0.05
3200
3000
2800
2600
2400
2200
2000
1800
1600
1400
1200
1000
800
600

Figure S16

*(3S,3′S)-1′,1′′-bis(4-methoxyphenyl)-1-tosyldispiro[indoline-3,2′-azetidine-3′,3′′-pyrrolidine]-2,2′′,4′-trione (**6**)*

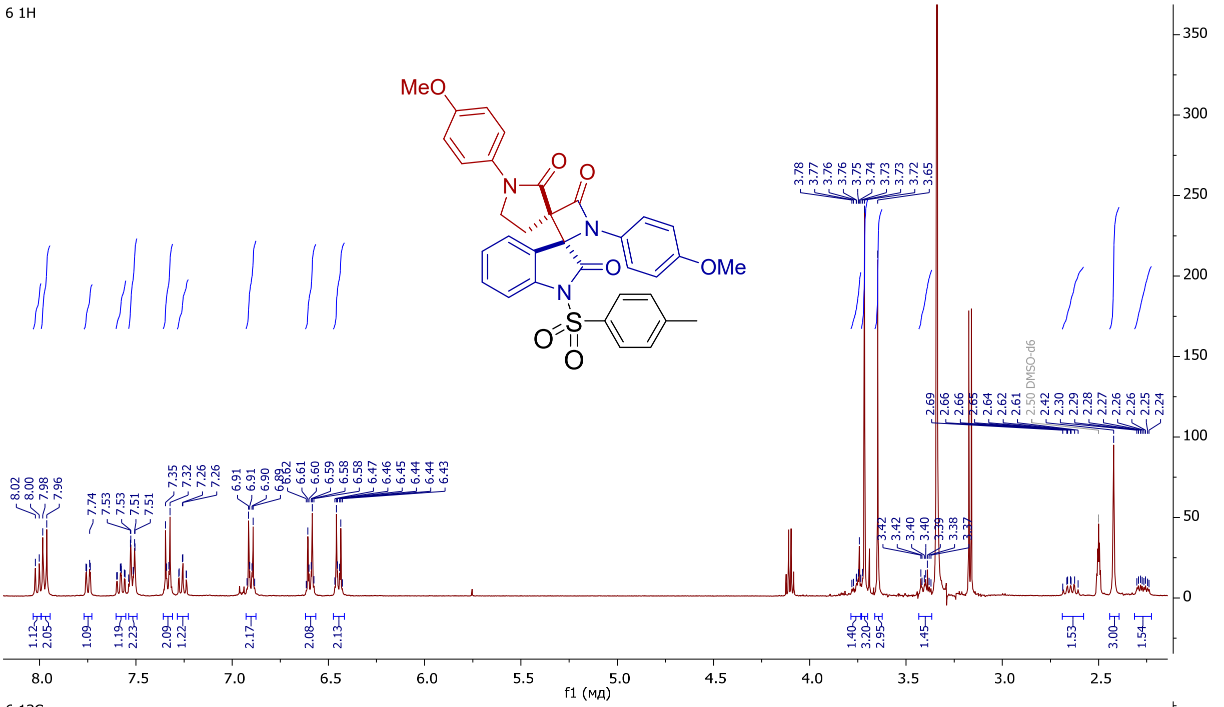

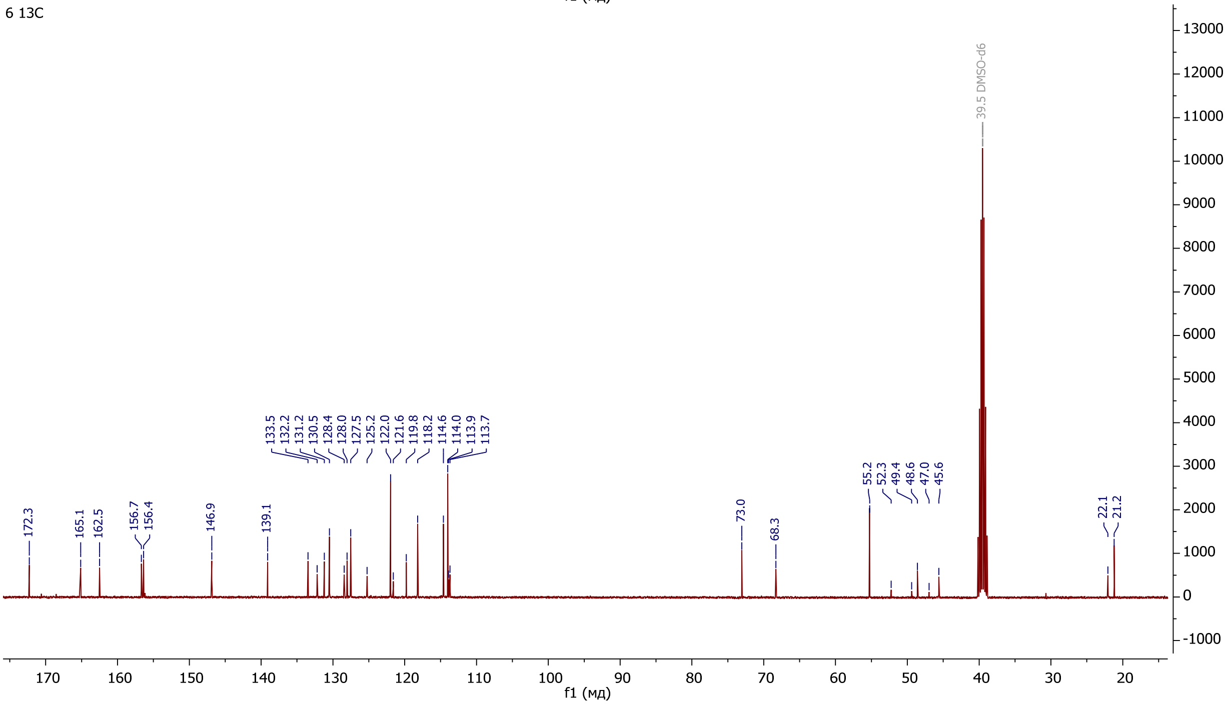

Figure S17

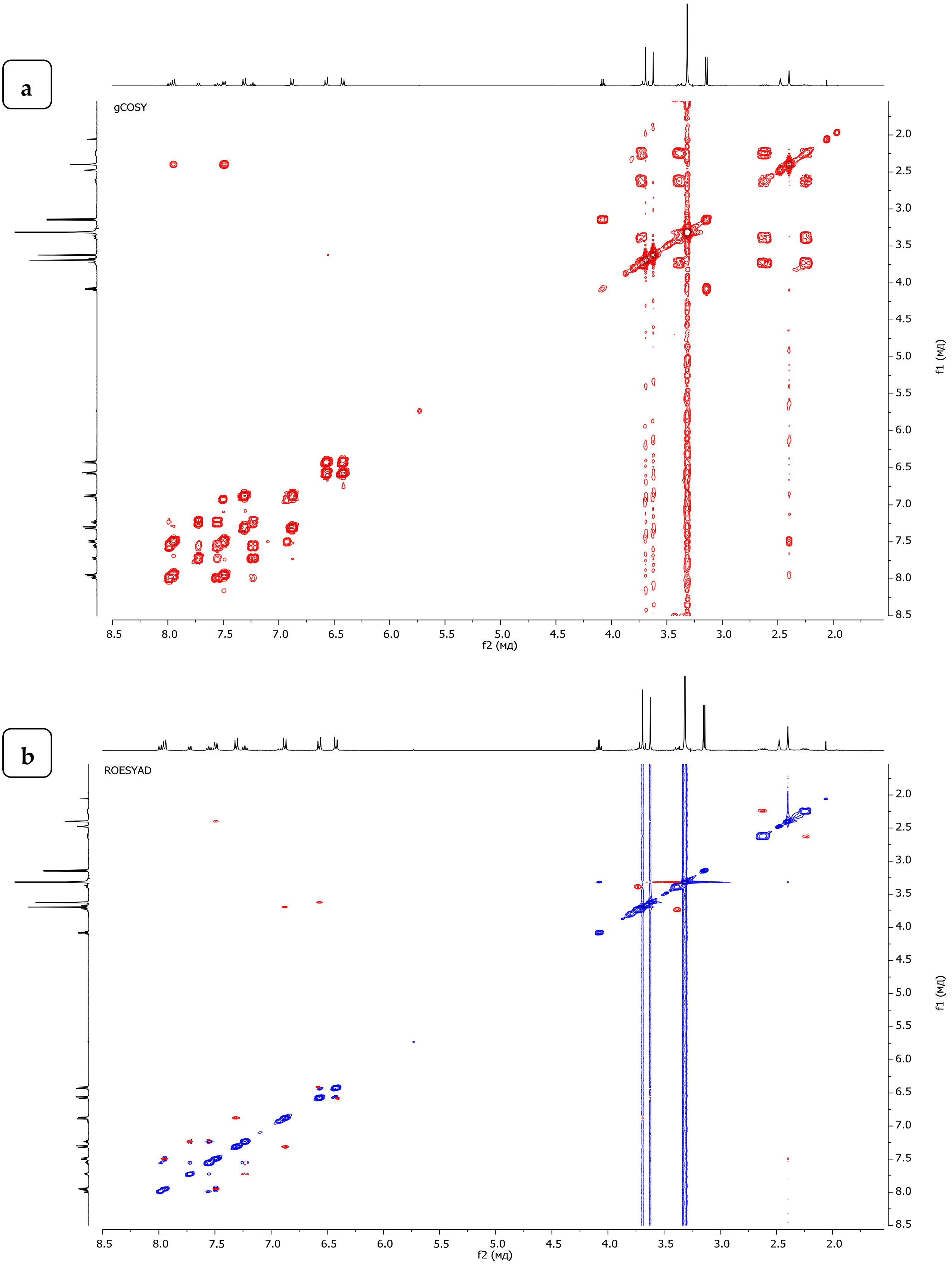

Figure S18: (a) gCOSY; (b)ROESYAD)

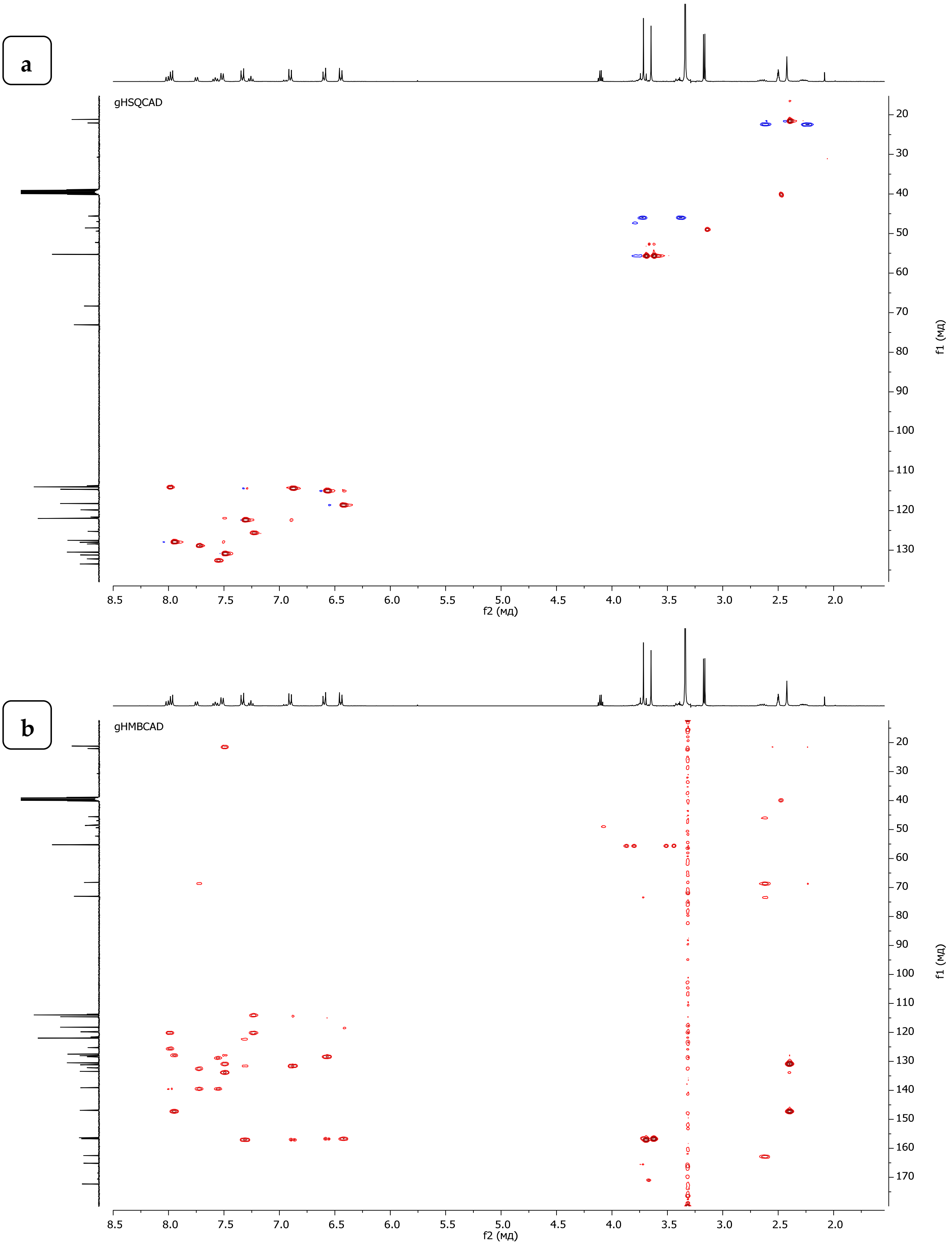

Figure S19: (a) gHSQCAD; (b)gHMBCAD

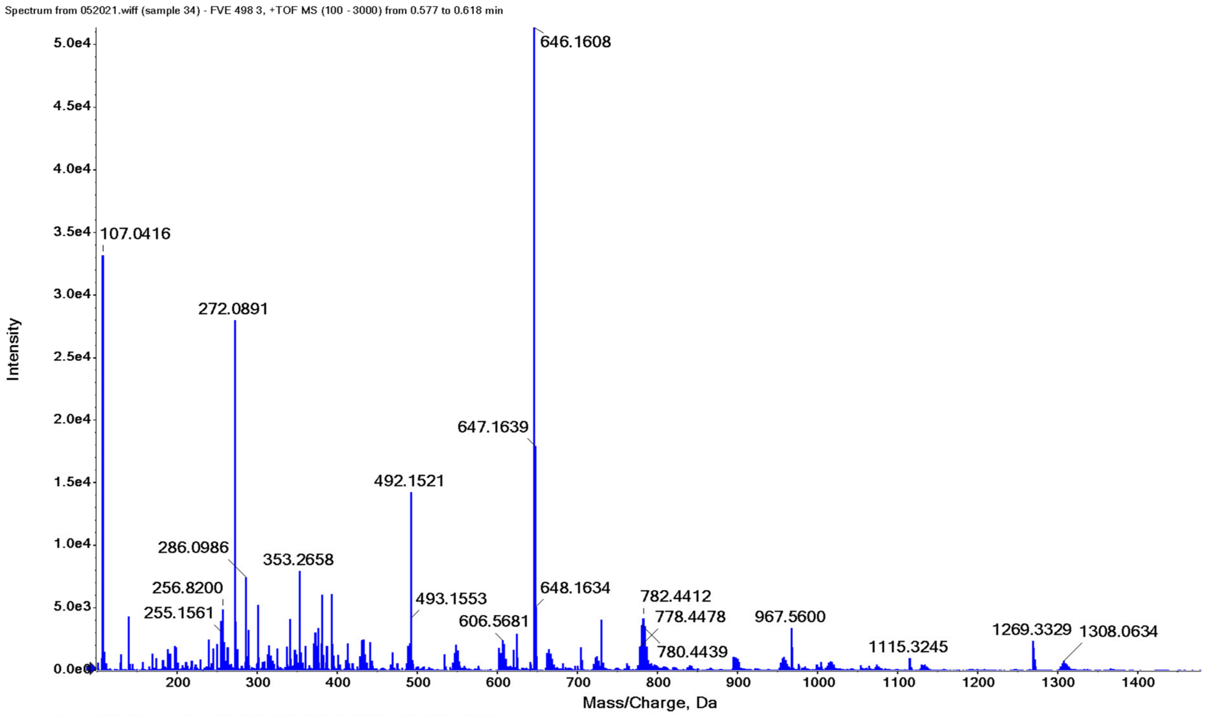
Spectrum from 052021.wiff (sample 34) - FVE 498 3, +TOF MS (100 - 3000) from 0.577 to 0.618 min
Intensity
Mass/Charge, Da
646.1608
107.0416
272.0891
647.1639
492.1521
286.0986
353.2658
256.8200
255.1561
493.1553
648.1634
606.5681
782.4412
778.4478
780.4439
967.5600
1115.3245
1269.3329
1308.0634

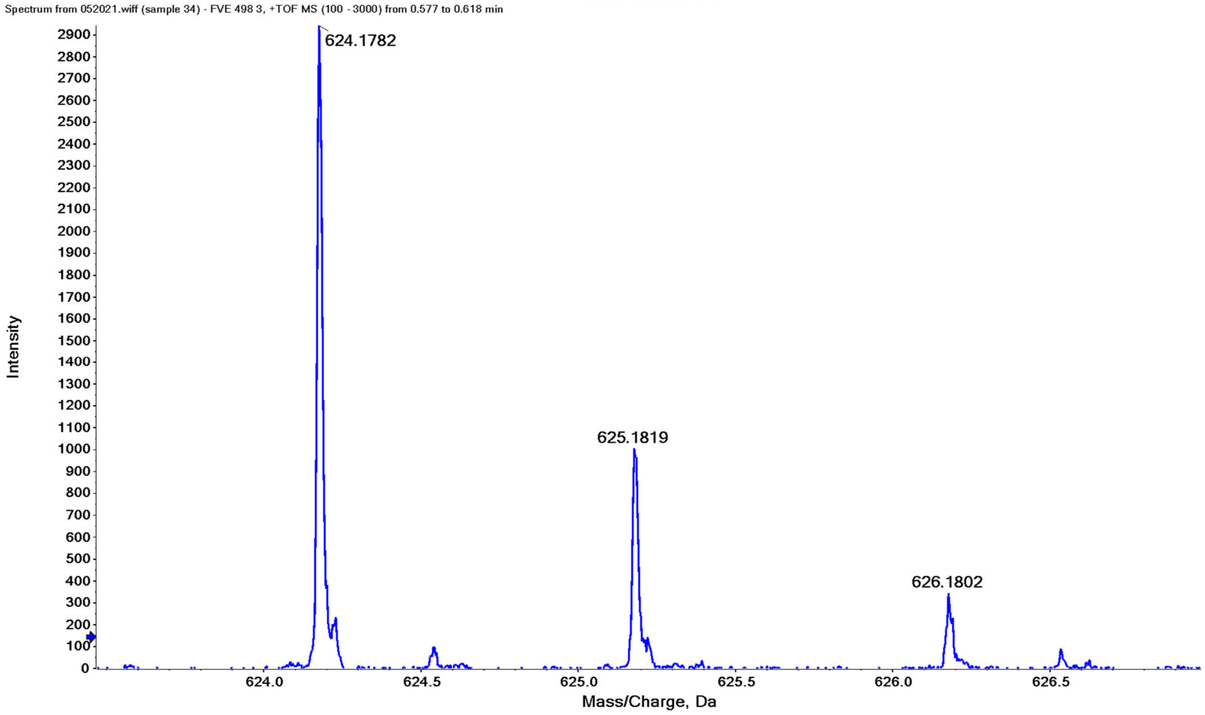
Spectrum from 052021.wiff (sample 34) - FVE 498 3, +TOF MS (100 - 3000) from 0.577 to 0.618 min
Intensity
Mass/Charge, Da
624.1782
625.1819
626.1802

Figure S20

N,1-bis(4-methoxyphenyl)-2-oxopyrrolidine-3-carboxamide (**7**)

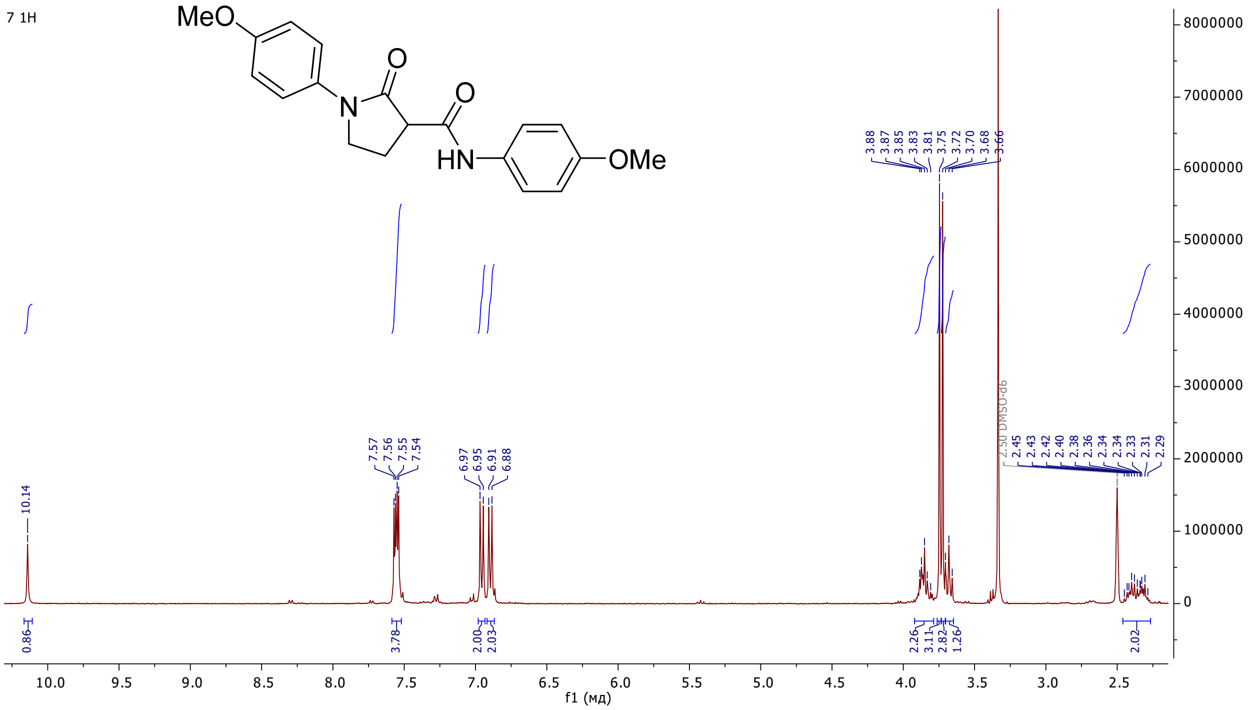

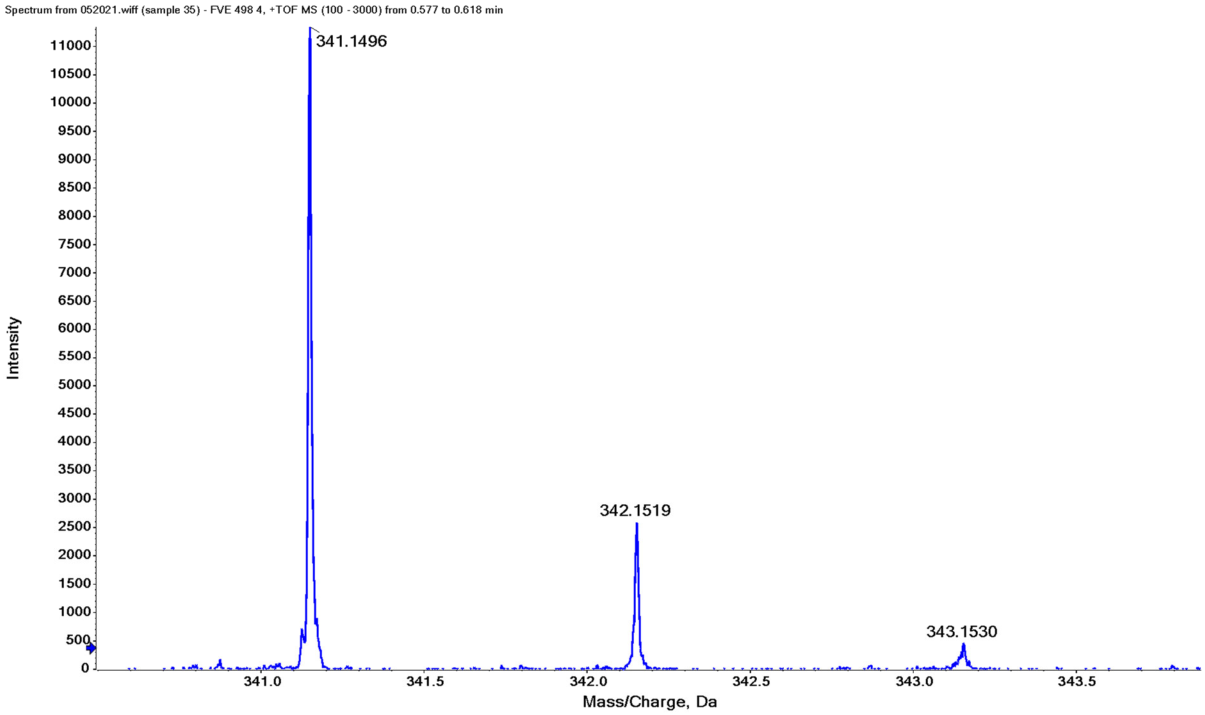

Figure S21

*(3S,3′S)-1′-(4-methoxyphenyl)-1′′-(p-tolyl)dispiro[indoline-3,2′-azetidine-3′,3′′-pyrrolidine]-2,2′′,4′-trione (**5b**)*

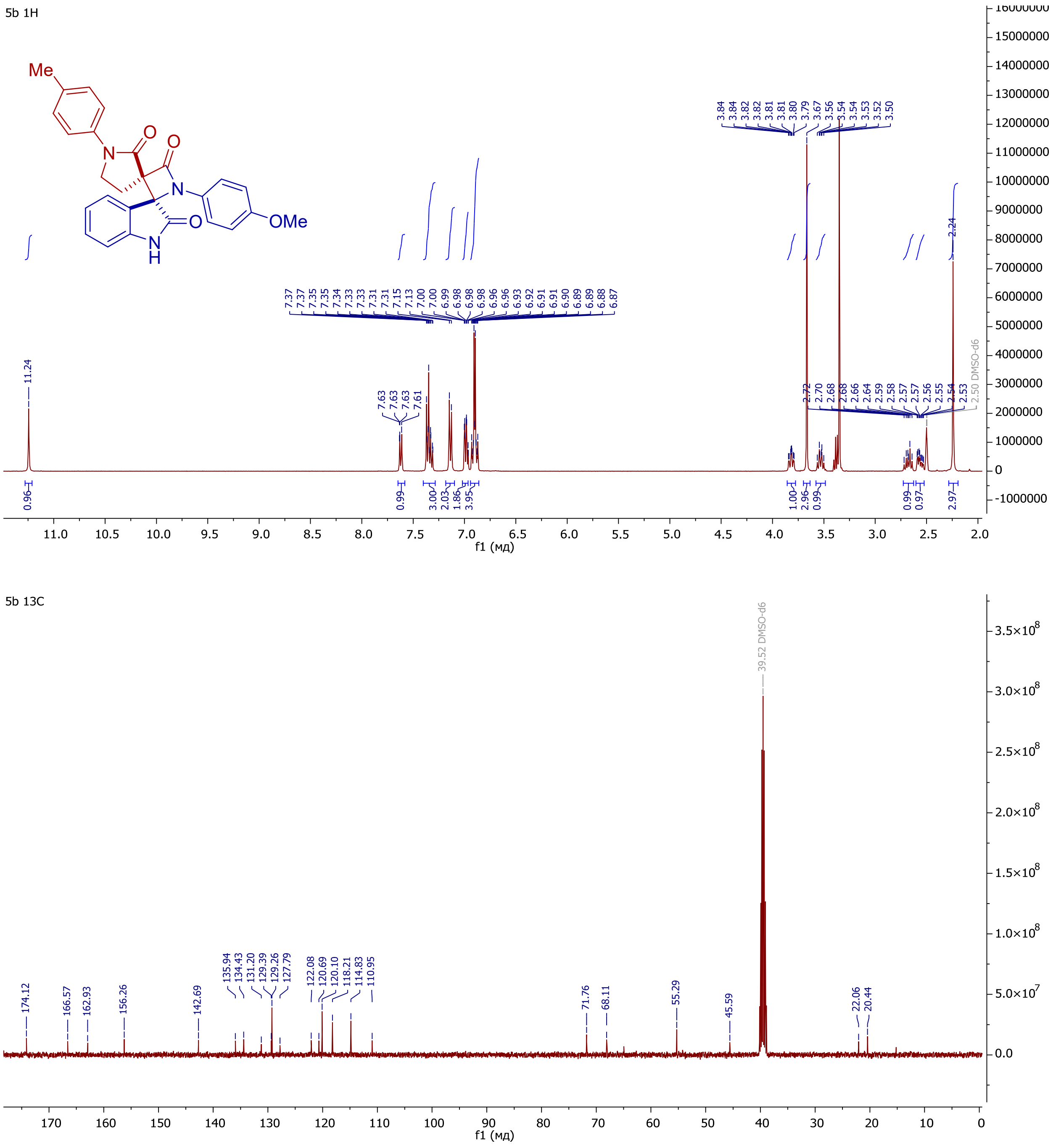

Figure S22

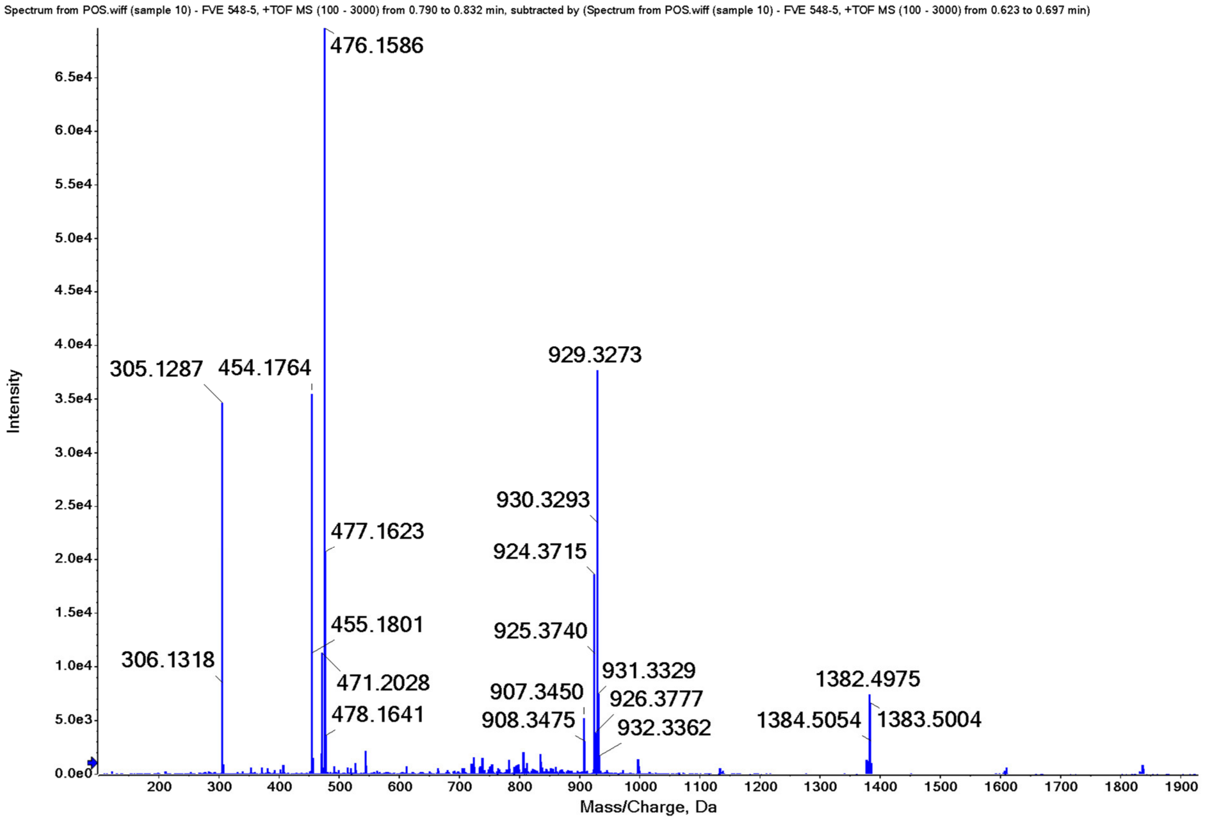
Spectrum from POS.wiff (sample 10) - FVE 548-5, +TOF MS (100 - 3000) from 0.790 to 0.832 min, subtracted by (Spectrum from POS.wiff (sample 10) - FVE 548-5, +TOF MS (100 - 3000) from 0.623 to 0.697 min)
476.1586
305.1287
454.1764
929.3273
930.3293
477.1623
924.3715
455.1801
925.3740
306.1318
931.3329
471.2028
907.3450
926.3777
478.1641
908.3475
932.3362
1382.4975
1384.5054
1383.5004
Intensity
Mass/Charge, Da

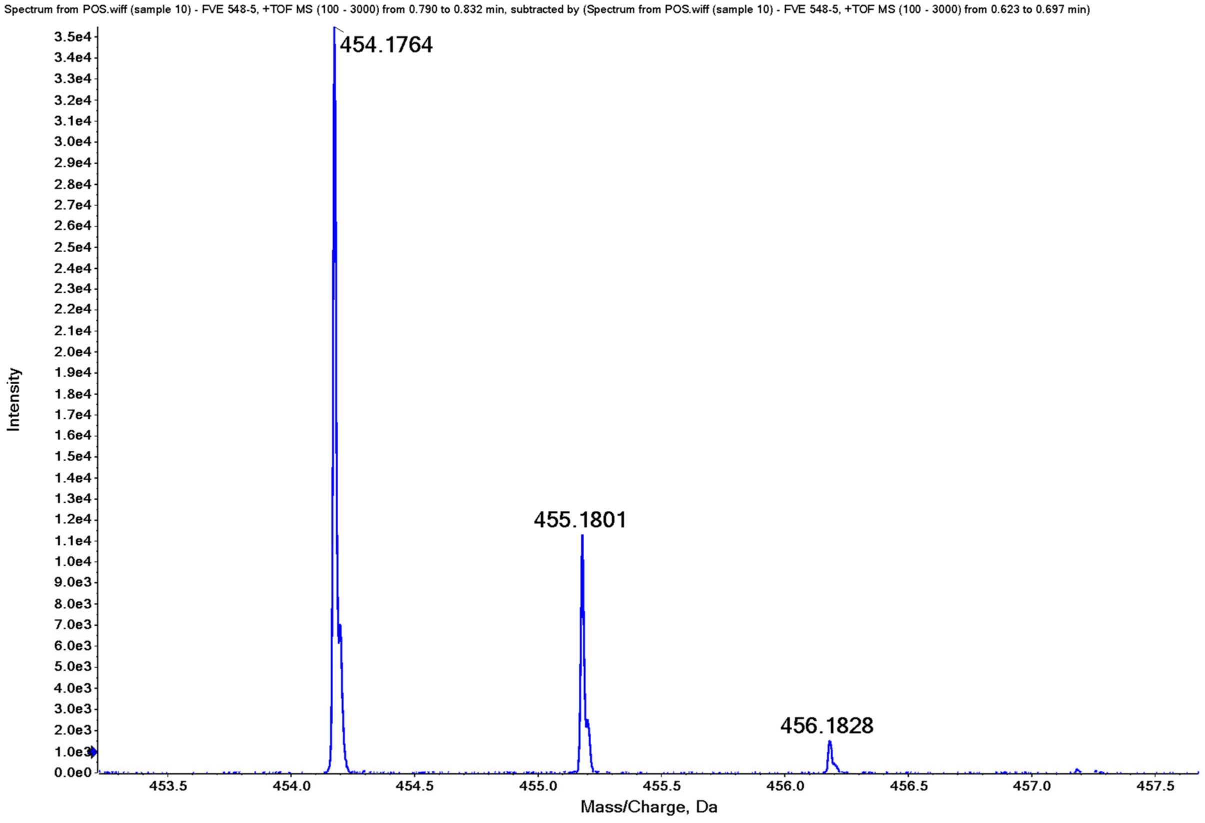
Spectrum from POS.wiff (sample 10) - FVE 548-5, +TOF MS (100 - 3000) from 0.790 to 0.832 min, subtracted by (Spectrum from POS.wiff (sample 10) - FVE 548-5, +TOF MS (100 - 3000) from 0.623 to 0.697 min)
454.1764
455.1801
456.1828
Intensity
Mass/Charge, Da

Figure S23

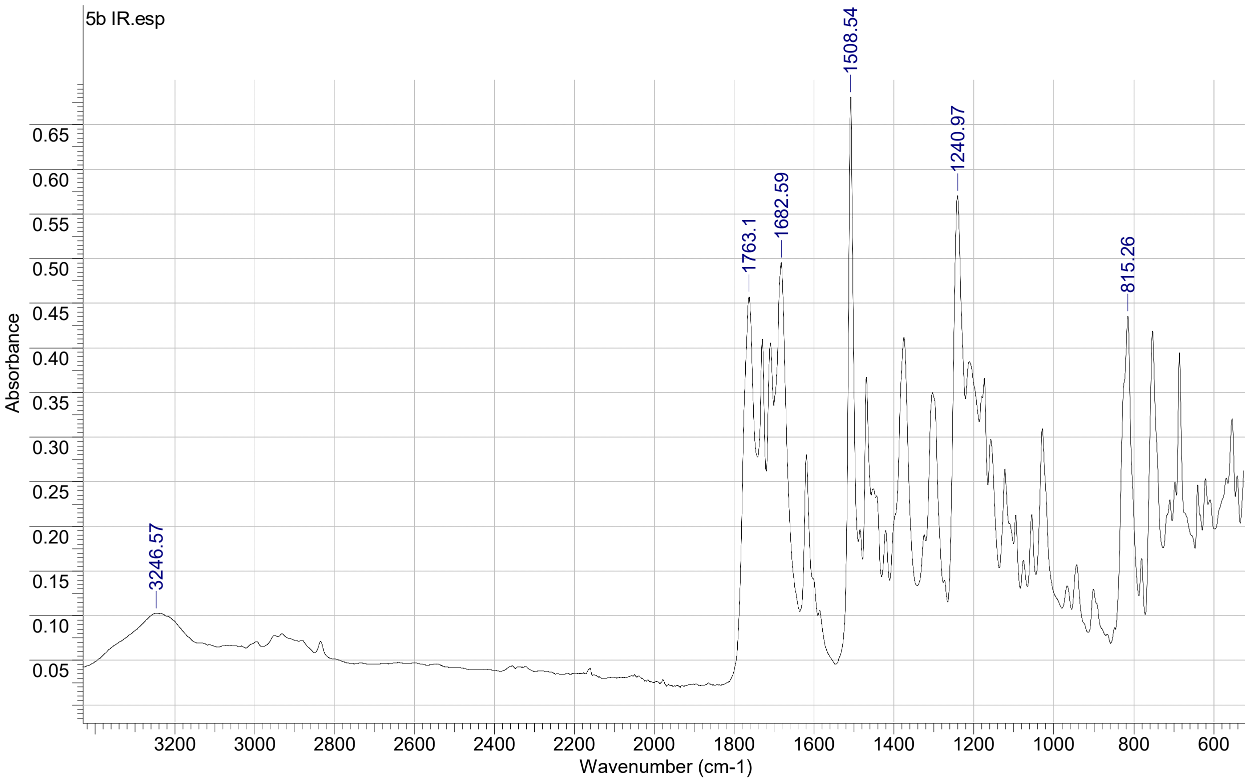
5b IR.esp
3246.57
1763.1
1682.59
1508.54
1240.97
815.26
Absorbance
0.65
0.60
0.55
0.50
0.45
0.40
0.35
0.30
0.25
0.20
0.15
0.10
0.05
3200
3000
2800
2600
2400
2200
2000
1800
1600
1400
1200
1000
800
600
Wavenumber (cm-1)
Figure S24

(3S,3′S)-1′′-(4-chlorophenyl)-1′-(4-methoxyphenyl)dispiro[indoline-3,2′-azetidine-3′,3′′-pyrrolidine]-2,2′′,4′-trione (***5c***)

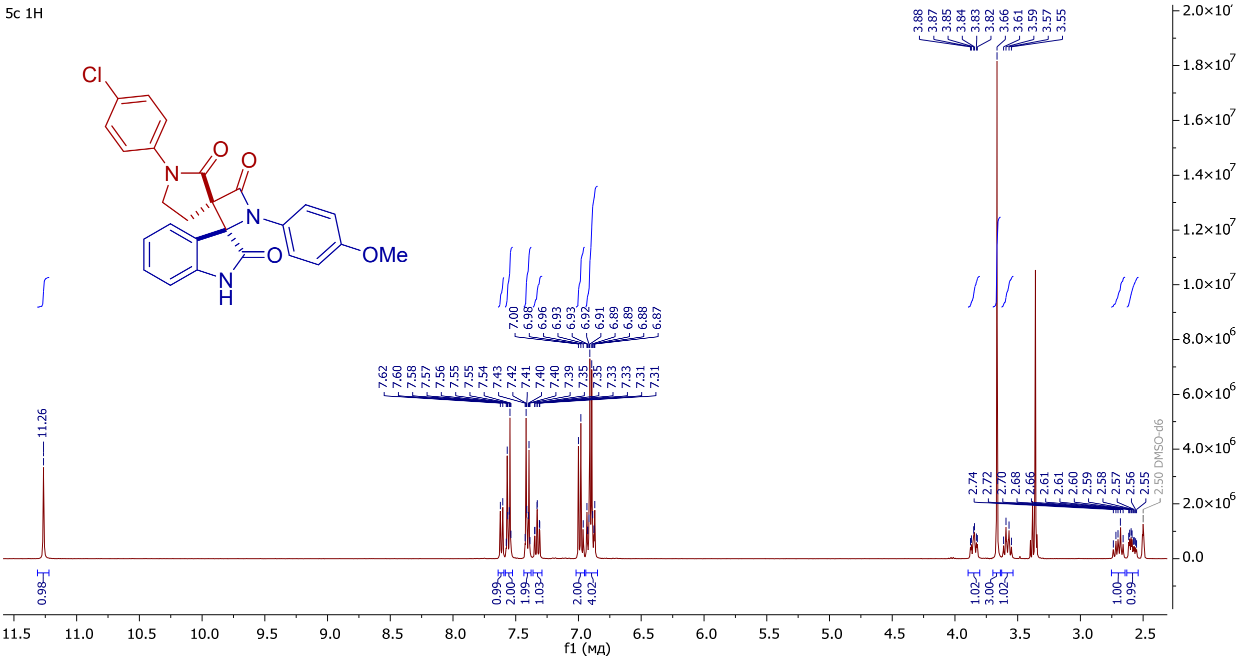

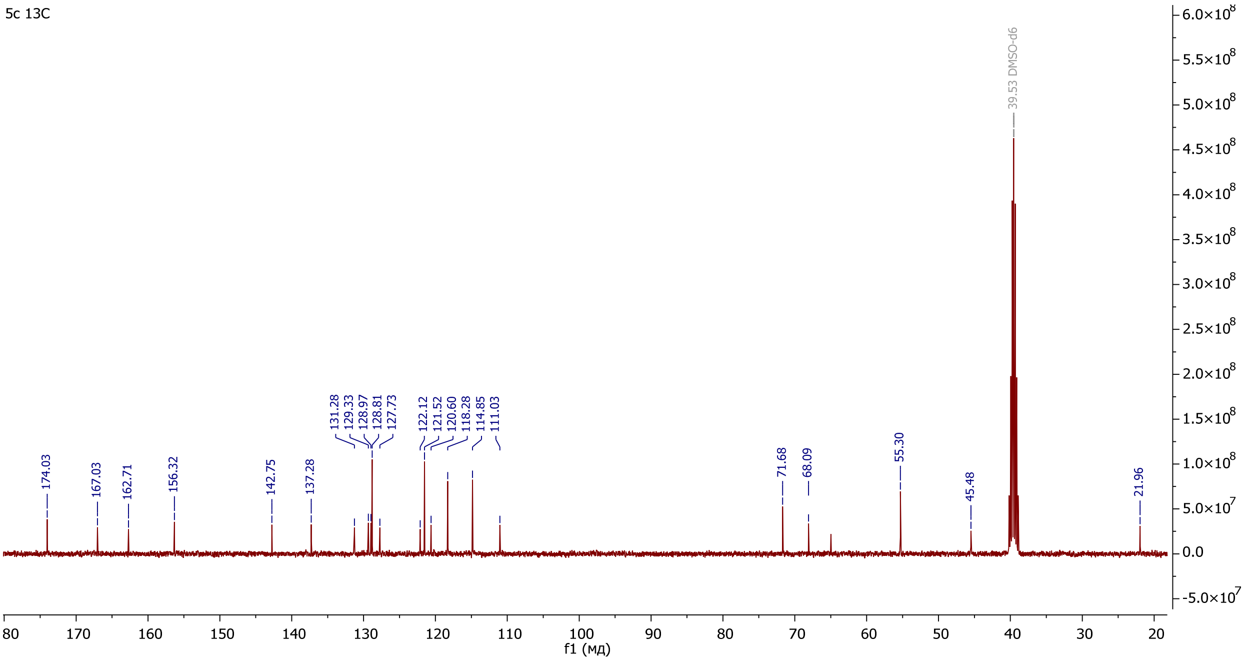

Figure S25

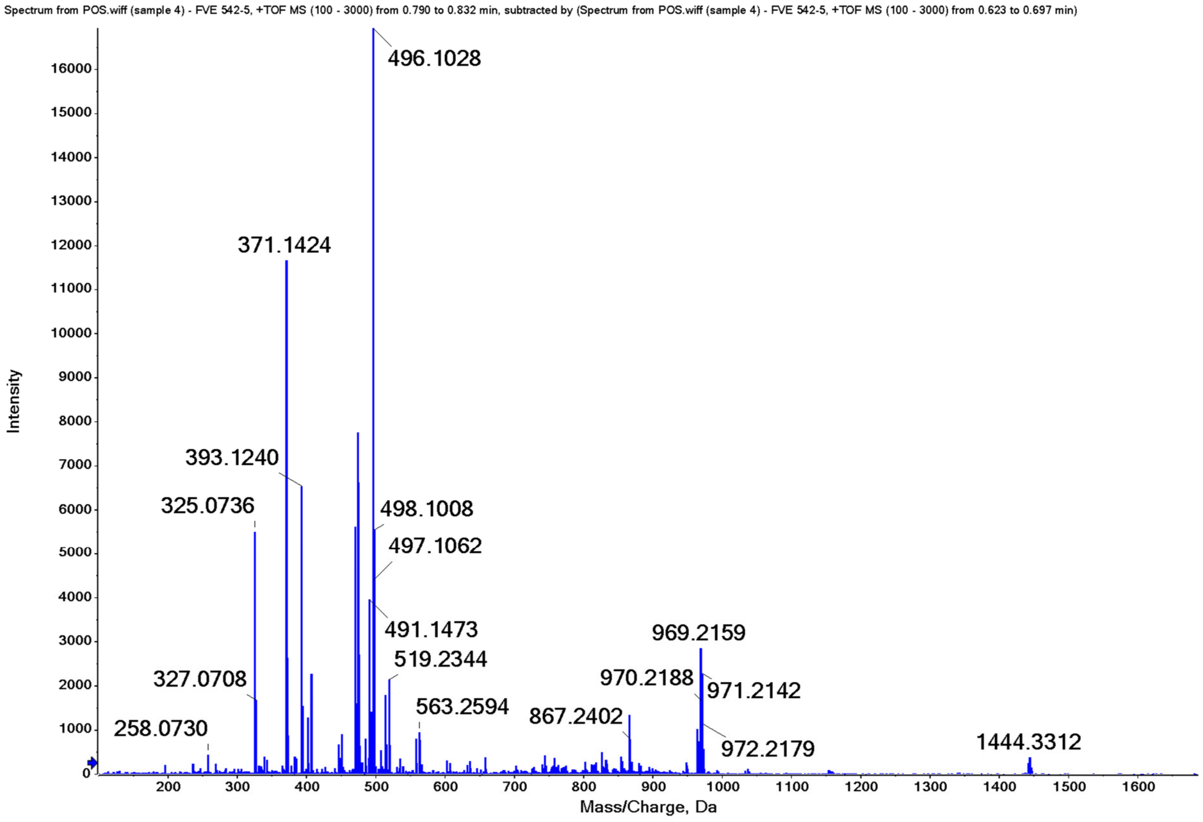

Spectrum from POS.wiff (sample 4) - FVE 542-5, +TOF MS (100 - 3000) from 0.790 to 0.832 min, subtracted by (Spectrum from POS.wiff (sample 4) - FVE 542-5, +TOF MS (100 - 3000) from 0.623 to 0.697 min)
Intensity
Mass/Charge, Da
496.1028
371.1424
393.1240
325.0736
498.1008
497.1062
491.1473
519.2344
327.0708
563.2594
258.0730
969.2159
970.2188
971.2142
867.2402
972.2179
1444.3312

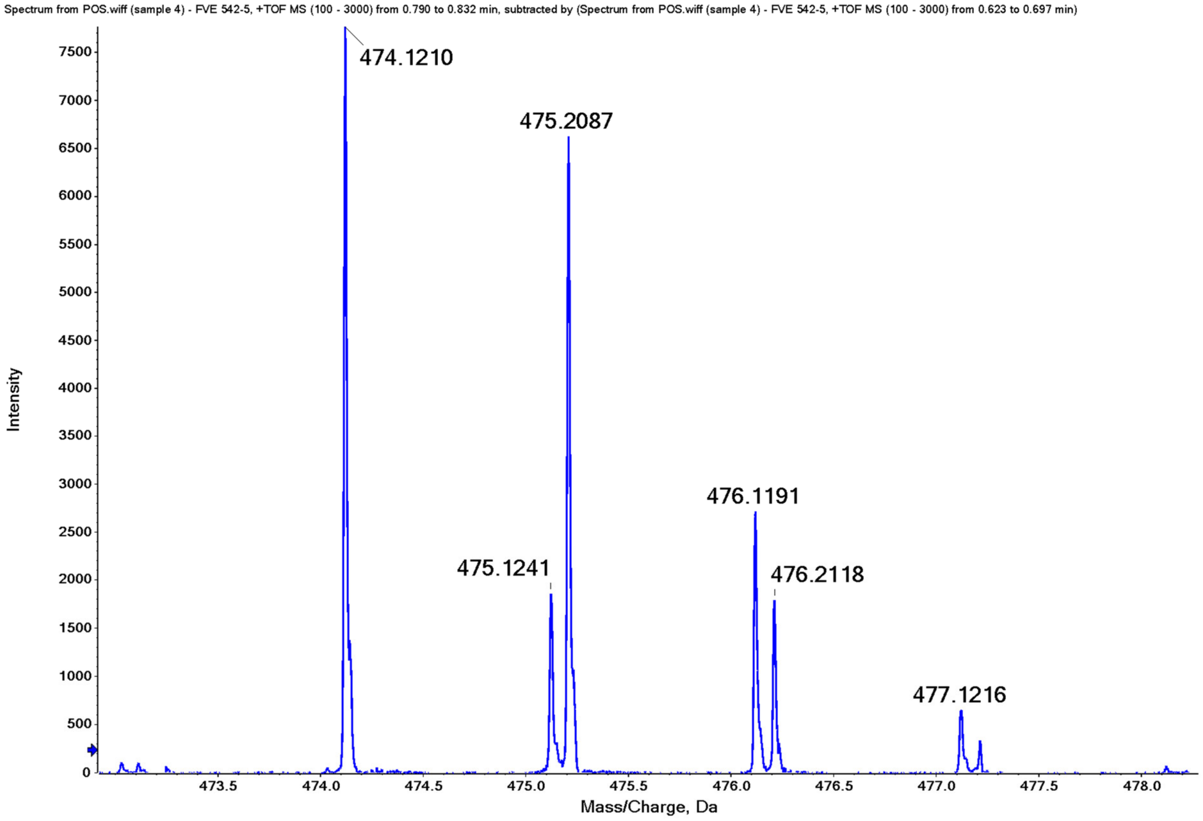

Spectrum from POS.wiff (sample 4) - FVE 542-5, +TOF MS (100 - 3000) from 0.790 to 0.832 min, subtracted by (Spectrum from POS.wiff (sample 4) - FVE 542-5, +TOF MS (100 - 3000) from 0.623 to 0.697 min)
Intensity
Mass/Charge, Da
474.1210
475.2087
476.1191
475.1241
476.2118
477.1216

Figure S26

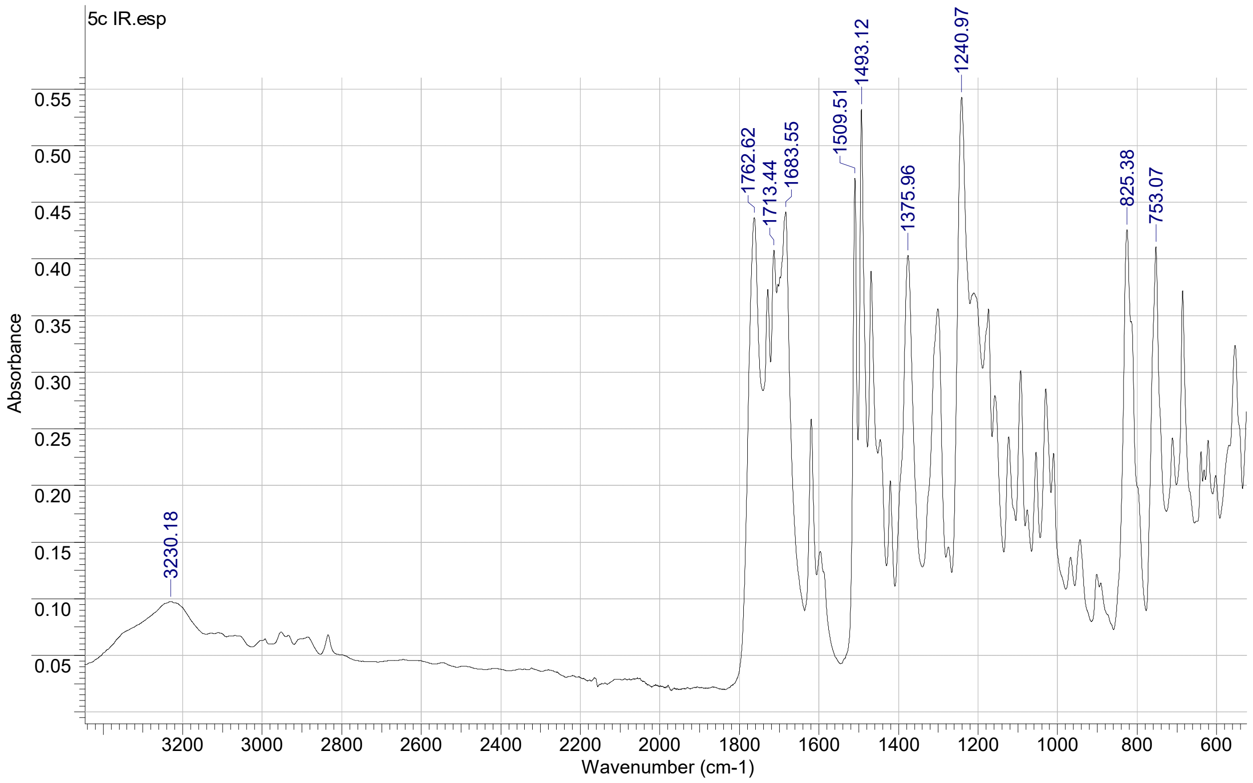
5c IR.esp
Absorbance
Wavenumber (cm-1)
0.55
0.50
0.45
0.40
0.35
0.30
0.25
0.20
0.15
0.10
0.05
3200
3000
2800
2600
2400
2200
2000
1800
1600
1400
1200
1000
800
600
3230.18
1762.62
1713.44
1683.55
1509.51
1493.12
1375.96
1240.97
825.38
753.07

Figure S27

*(3S,3′S)-1′,1′′-di-m-tolyldispiro[indoline-3,2′-azetidine-3′,3′′-pyrrolidine]-2,2′′,4′-trione (**5d**)*

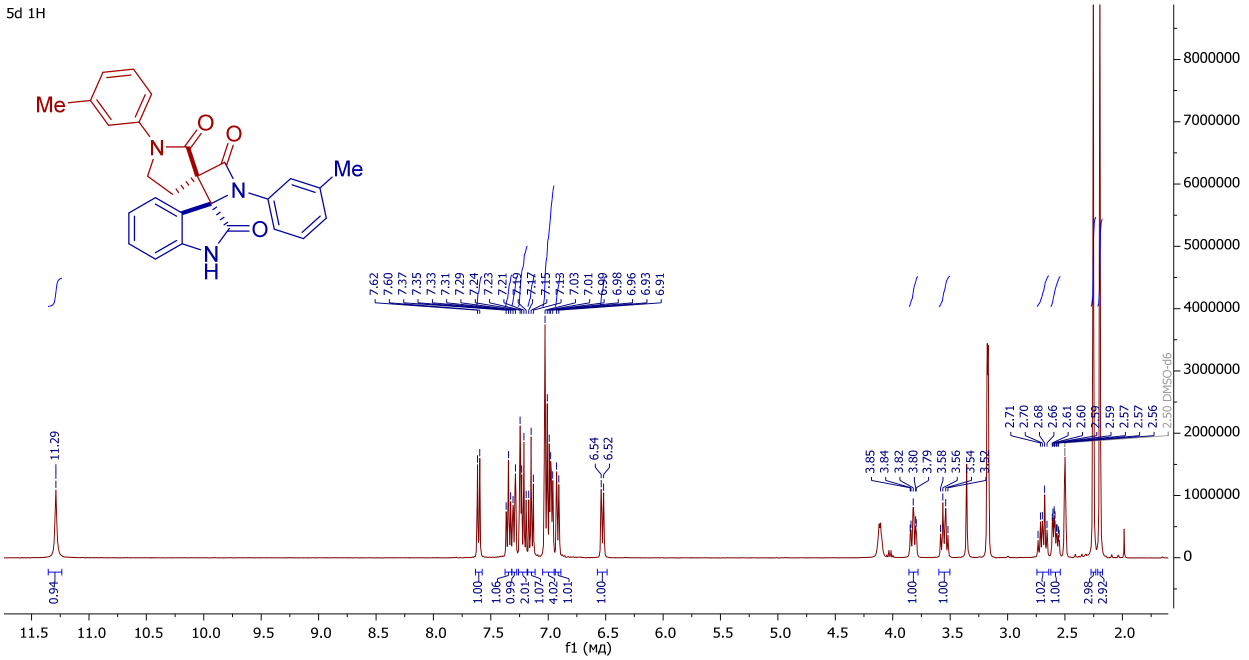

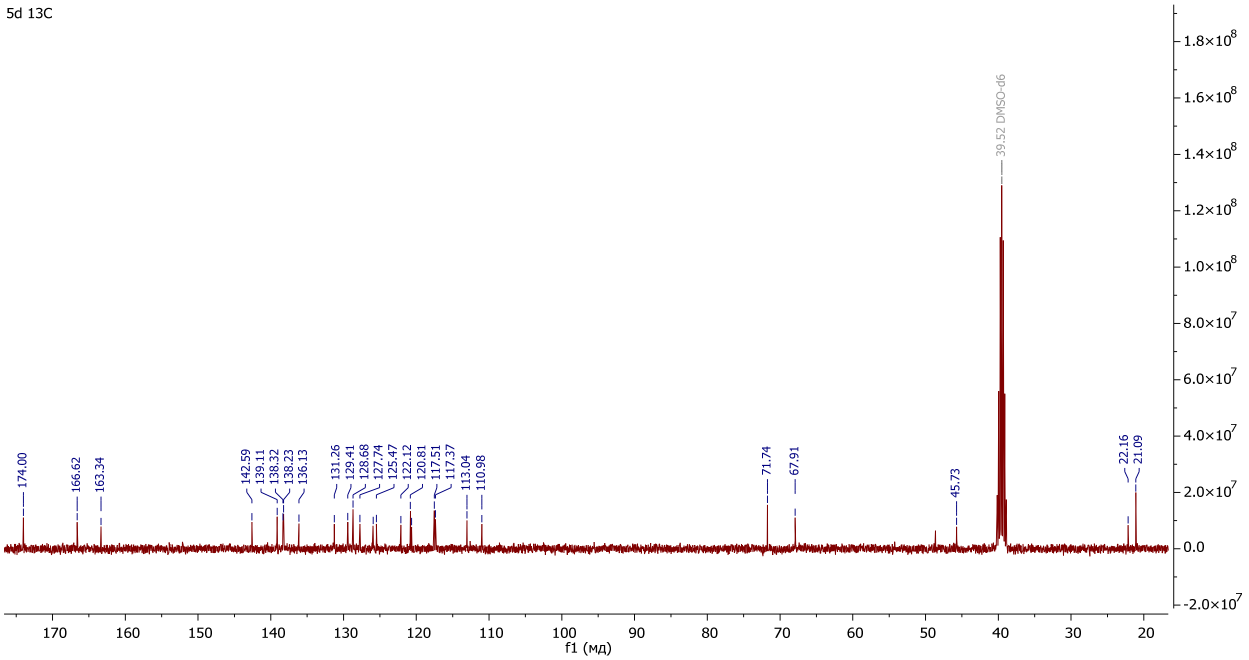

Figure S28

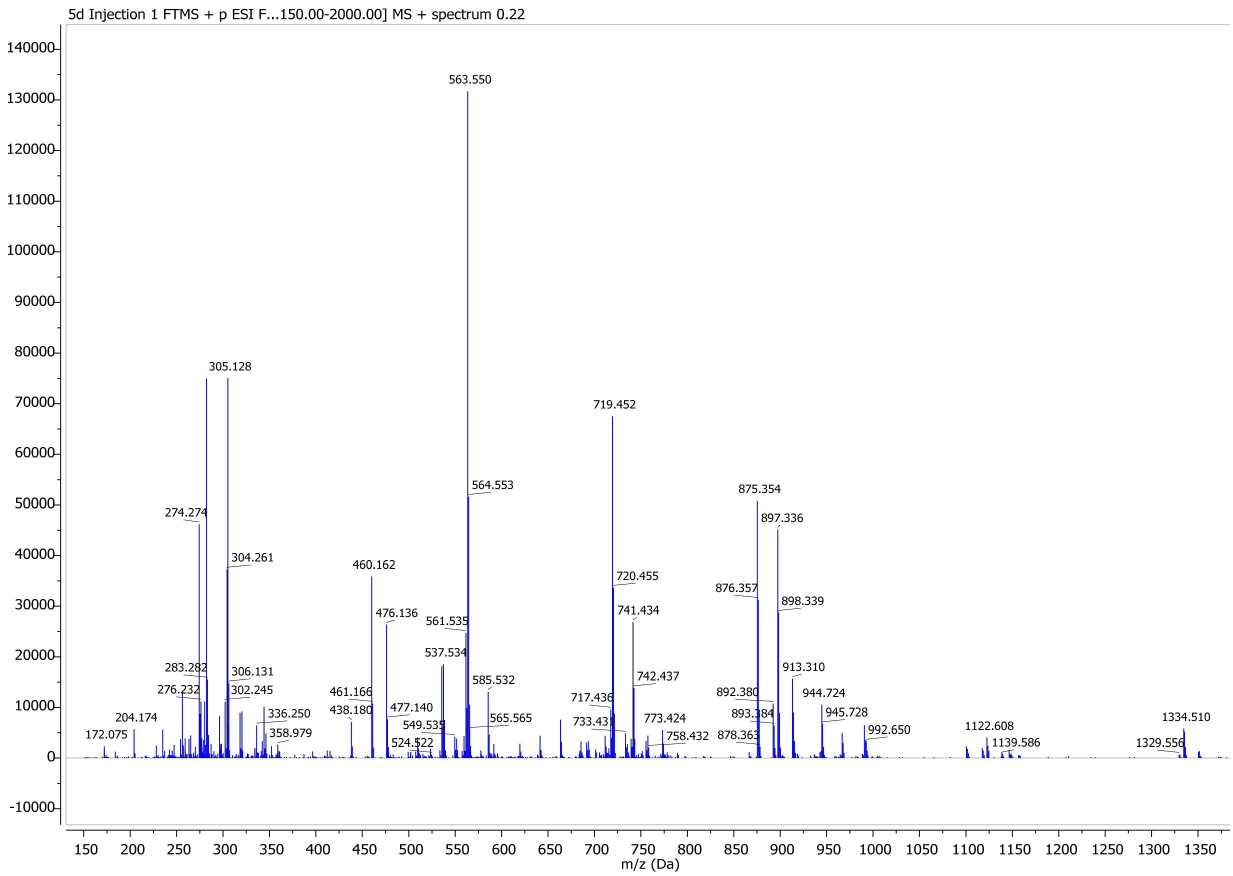
5d Injection 1 FTMS + p ESI F...150.00-2000.00] MS + spectrum 0.22
563.550
305.128
719.452
875.354
897.336
274.274
304.261
460.162
564.553
720.455
741.434
876.357
898.339
476.136
561.535
537.534
913.310
283.282
306.131
585.532
742.437
276.232
302.245
461.166
717.436
892.380
944.724
204.174
336.250
438.180
477.140
565.565
773.424
893.384
945.728
1334.510
172.075
358.979
549.535
733.431
758.432
878.363
992.650
1122.608
524.522
1139.586
1329.556
m/z (Da)

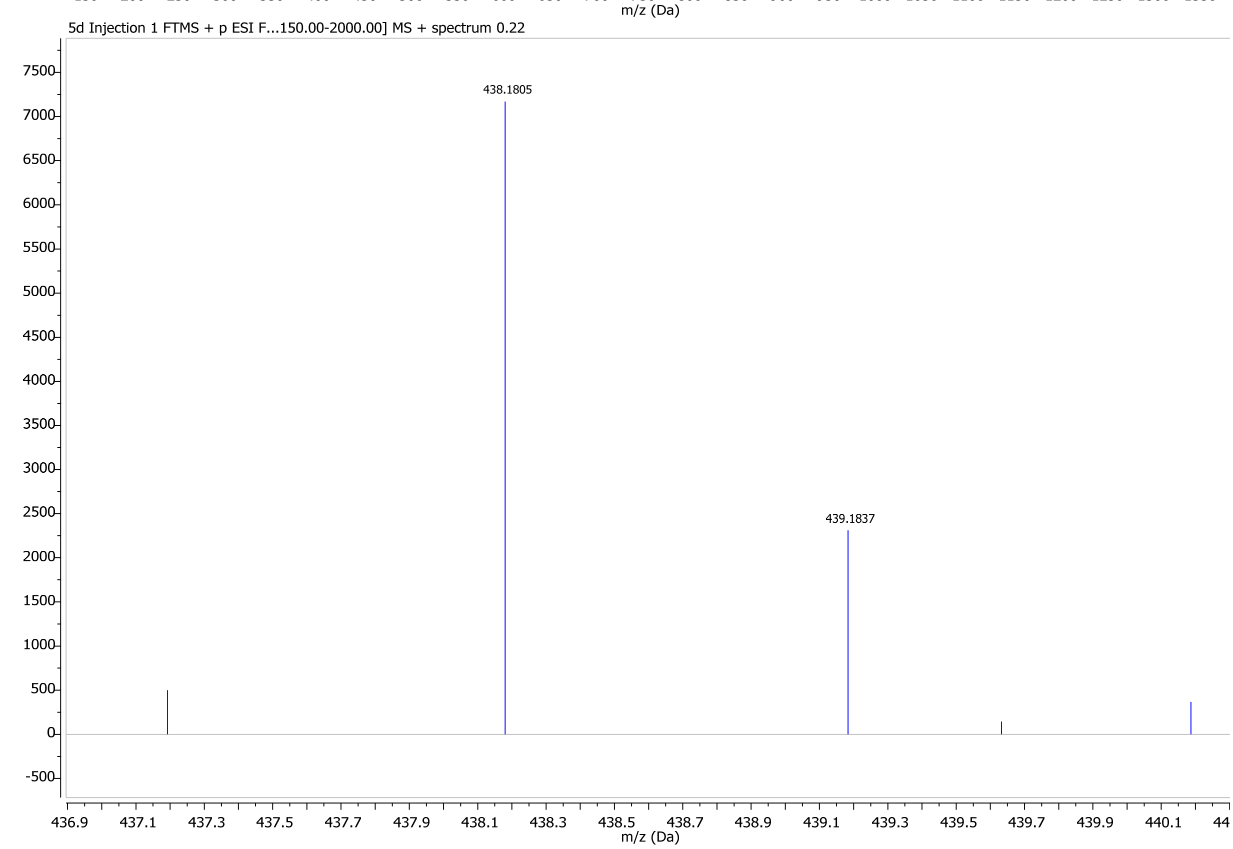
5d Injection 1 FTMS + p ESI F...150.00-2000.00] MS + spectrum 0.22
438.1805
439.1837
m/z (Da)

Figure S29

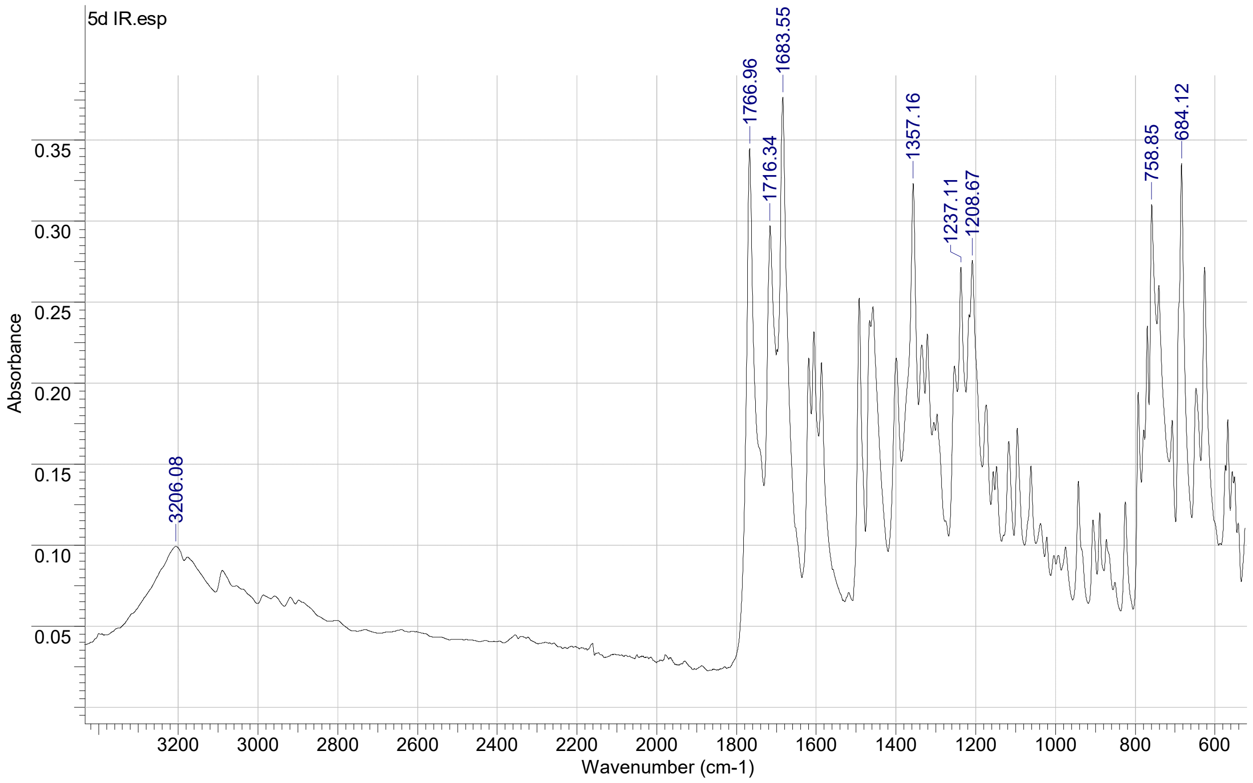
5d IR.esp
Absorbance
Wavenumber (cm-1)
3206.08
1766.96
1716.34
1683.55
1357.16
1237.11
1208.67
758.85
684.12
0.35
0.30
0.25
0.20
0.15
0.10
0.05
3200
3000
2800
2600
2400
2200
2000
1800
1600
1400
1200
1000
800
600

Figure S30

*(3S,3′S)-1′′-(4-chlorophenyl)-1′-(m-tolyl)dispiro[indoline-3,2′-azetidine-3′,3′′-pyrrolidine]-2,2′′,4′-trione (**5e**)*

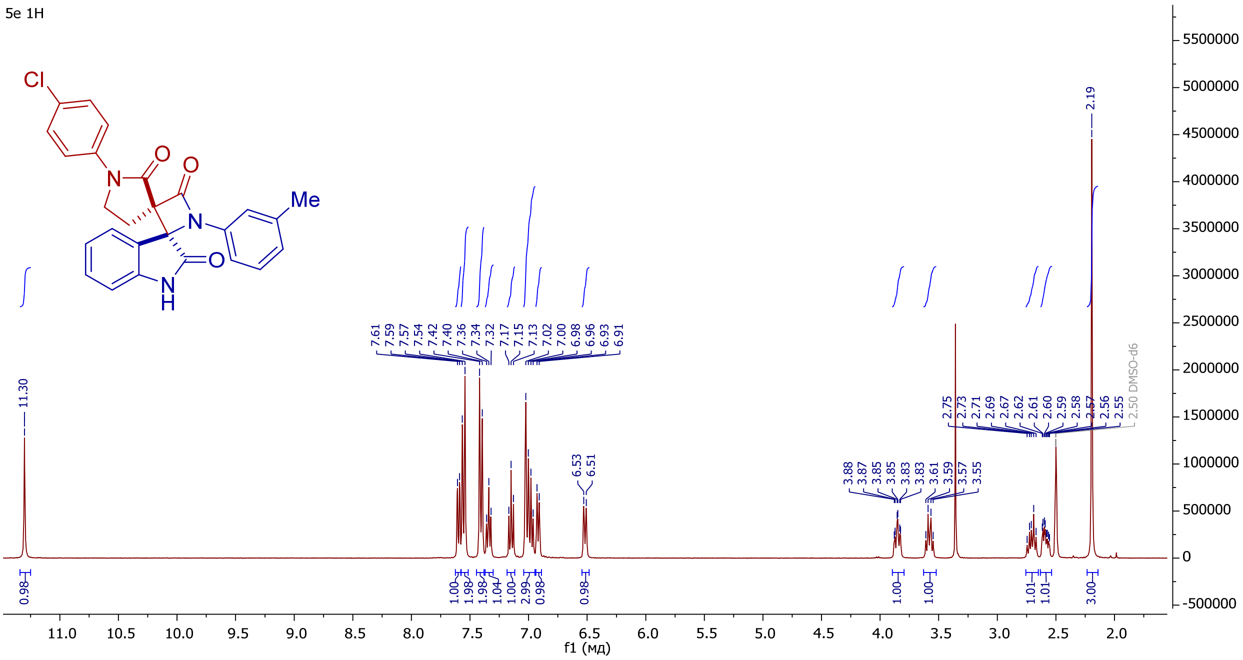

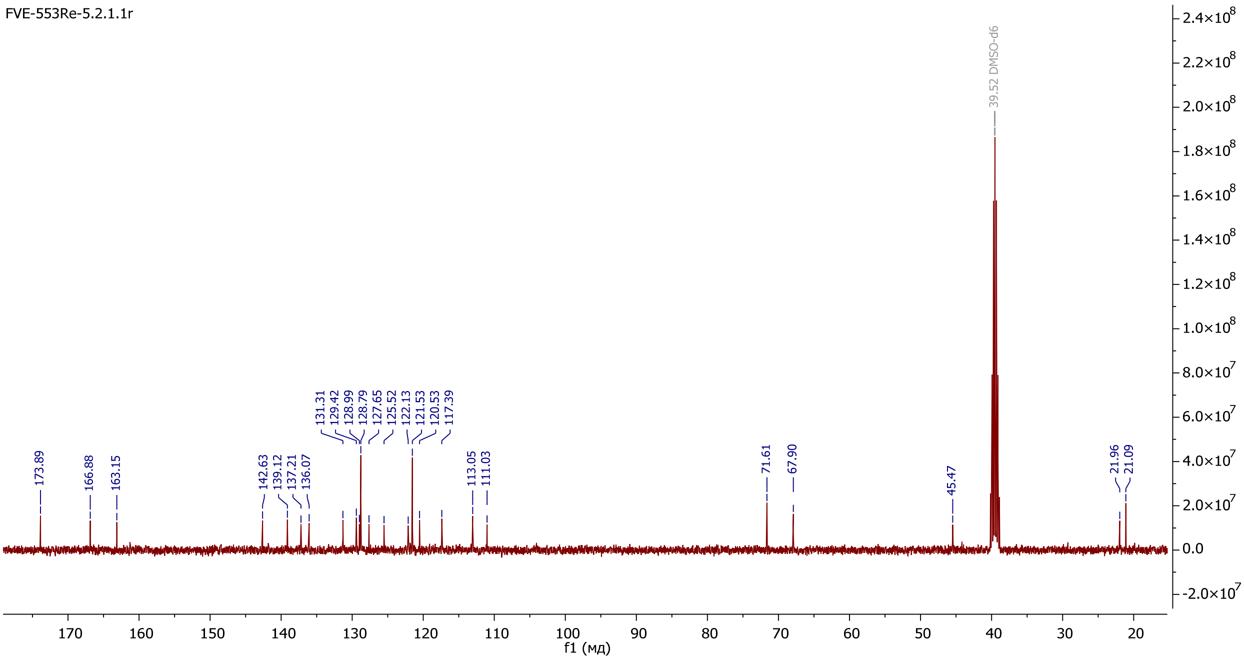

Figure S31

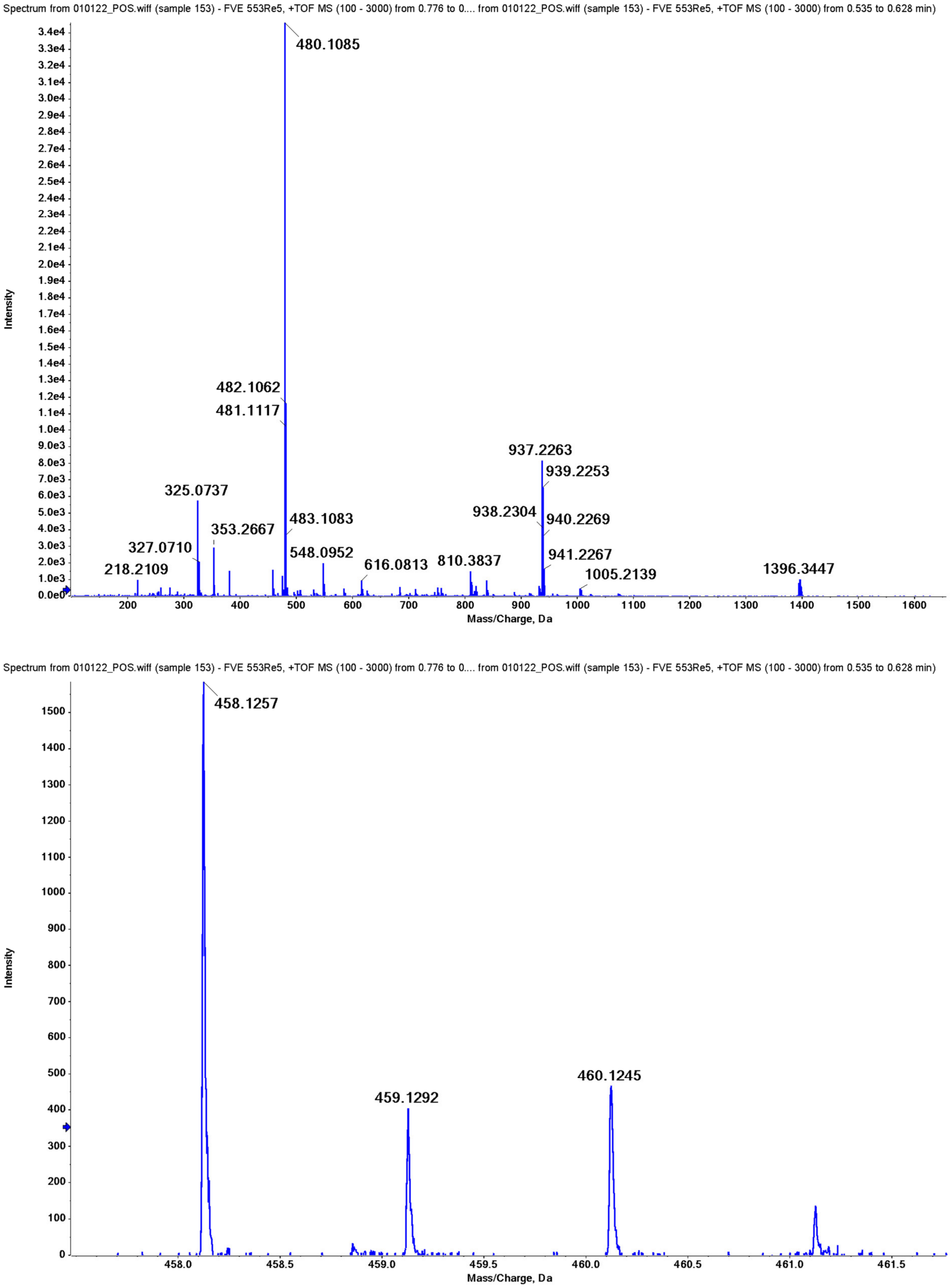
Spectrum from 010122_POS.wiff (sample 153) - FVE 553Re5, +TOF MS (100 - 3000) from 0.776 to 0.... from 010122_POS.wiff (sample 153) - FVE 553Re5, +TOF MS (100 - 3000) from 0.535 to 0.628 min)
480.1085
482.1062
481.1117
937.2263
939.2253
325.0737
938.2304
940.2269
483.1083
353.2667
327.0710
548.0952
941.2267
218.2109
616.0813
810.3837
1005.2139
1396.3447
Intensity
Mass/Charge, Da
Spectrum from 010122_POS.wiff (sample 153) - FVE 553Re5, +TOF MS (100 - 3000) from 0.776 to 0.... from 010122_POS.wiff (sample 153) - FVE 553Re5, +TOF MS (100 - 3000) from 0.535 to 0.628 min)
458.1257
460.1245
459.1292
Intensity
Mass/Charge, Da

Figure S32

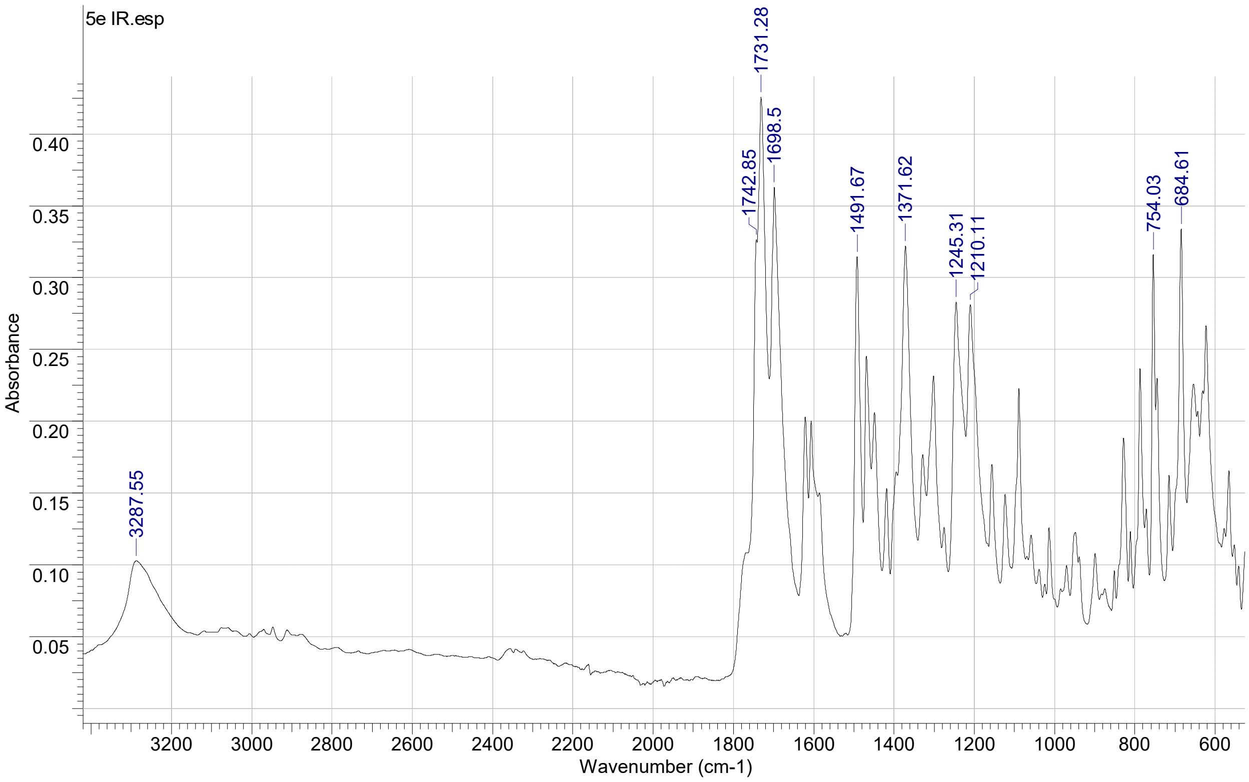
5e IR.esp
3287.55
1742.85
1731.28
1698.5
1491.67
1371.62
1245.31
1210.11
754.03
684.61
Absorbance
0.40
0.35
0.30
0.25
0.20
0.15
0.10
0.05
3200
3000
2800
2600
2400
2200
2000
1800
1600
1400
1200
1000
800
600
Wavenumber (cm-1)

Figure S33

*(3S,3′S)-1′′-(3-chloro-4-fluorophenyl)-1′-(m-tolyl)dispiro[indoline-3,2′-azetidine-3′,3′′-pyrrolidine]-2,2′′,4′-trione (**5f**)*

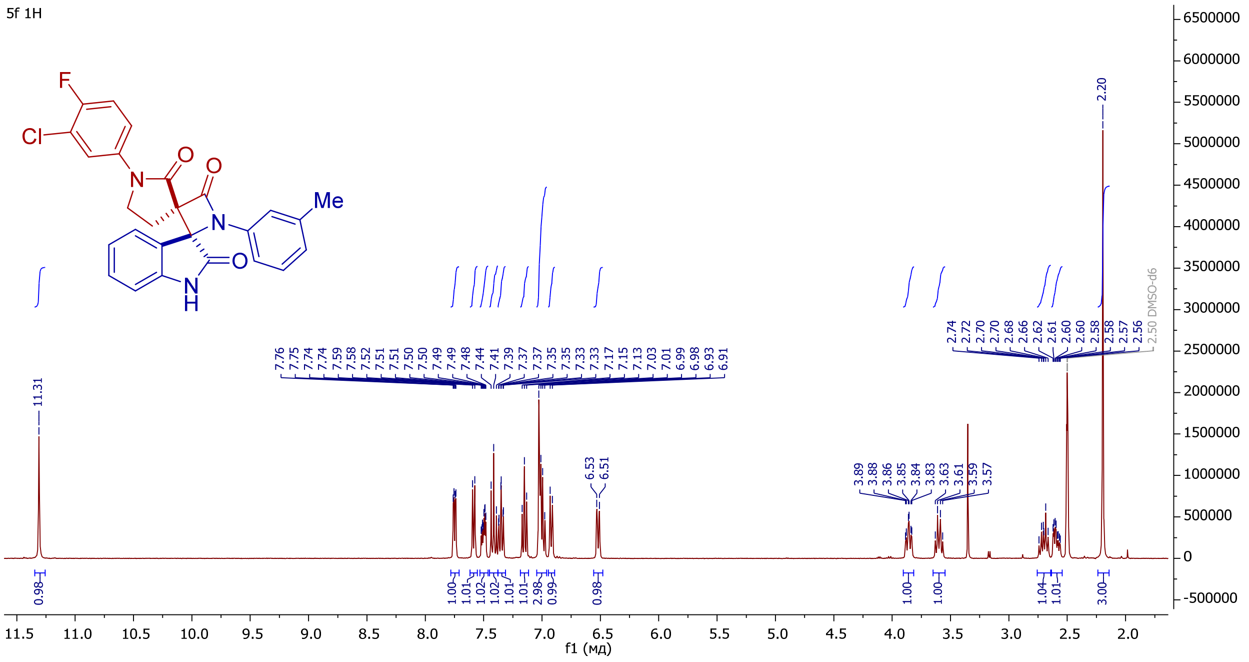

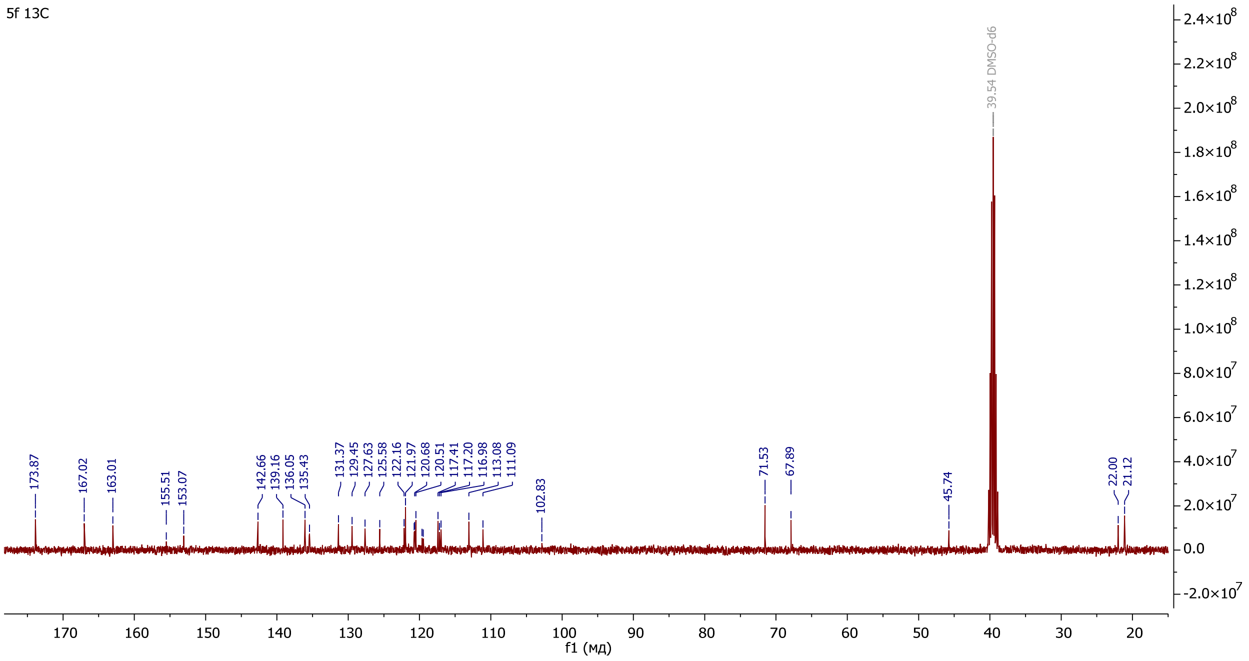

Figure S34

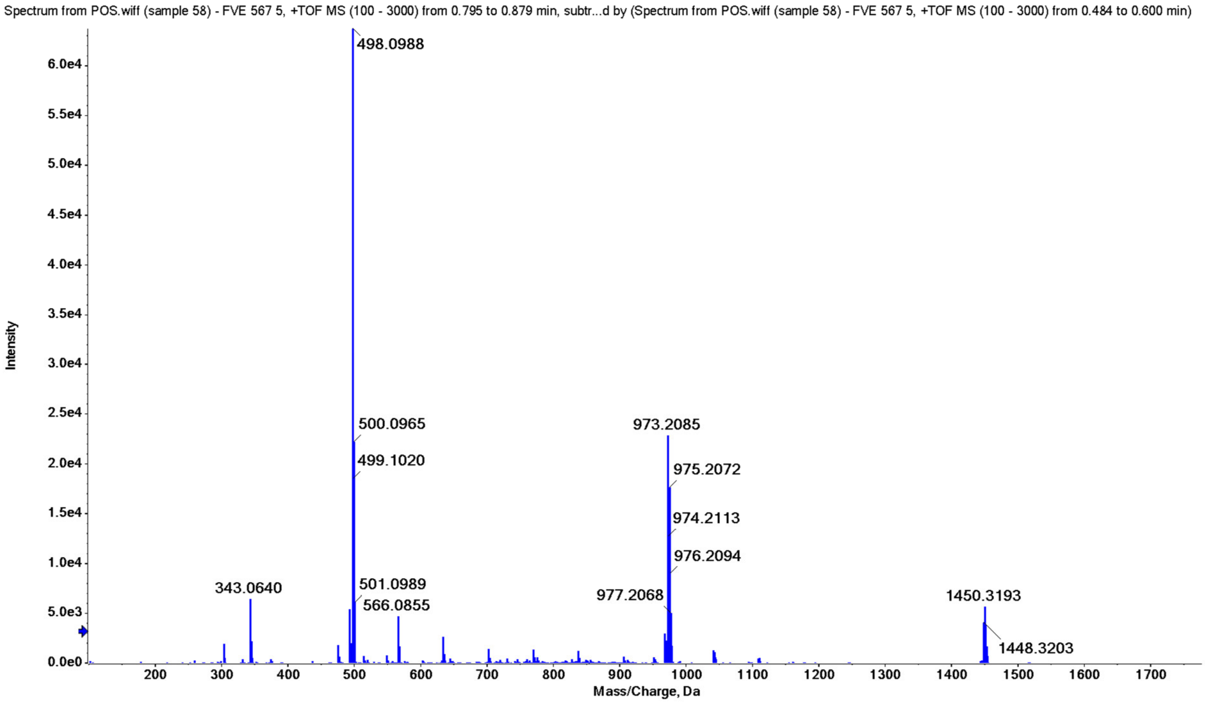

Spectrum from POS.wiff (sample 58) - FVE 567 5, +TOF MS (100 - 3000) from 0.795 to 0.879 min, subtr...d by (Spectrum from POS.wiff (sample 58) - FVE 567 5, +TOF MS (100 - 3000) from 0.484 to 0.600 min)
498.0988
500.0965
499.1020
501.0989
566.0855
343.0640
973.2085
975.2072
974.2113
976.2094
977.2068
1450.3193
1448.3203
Intensity
Mass/Charge, Da

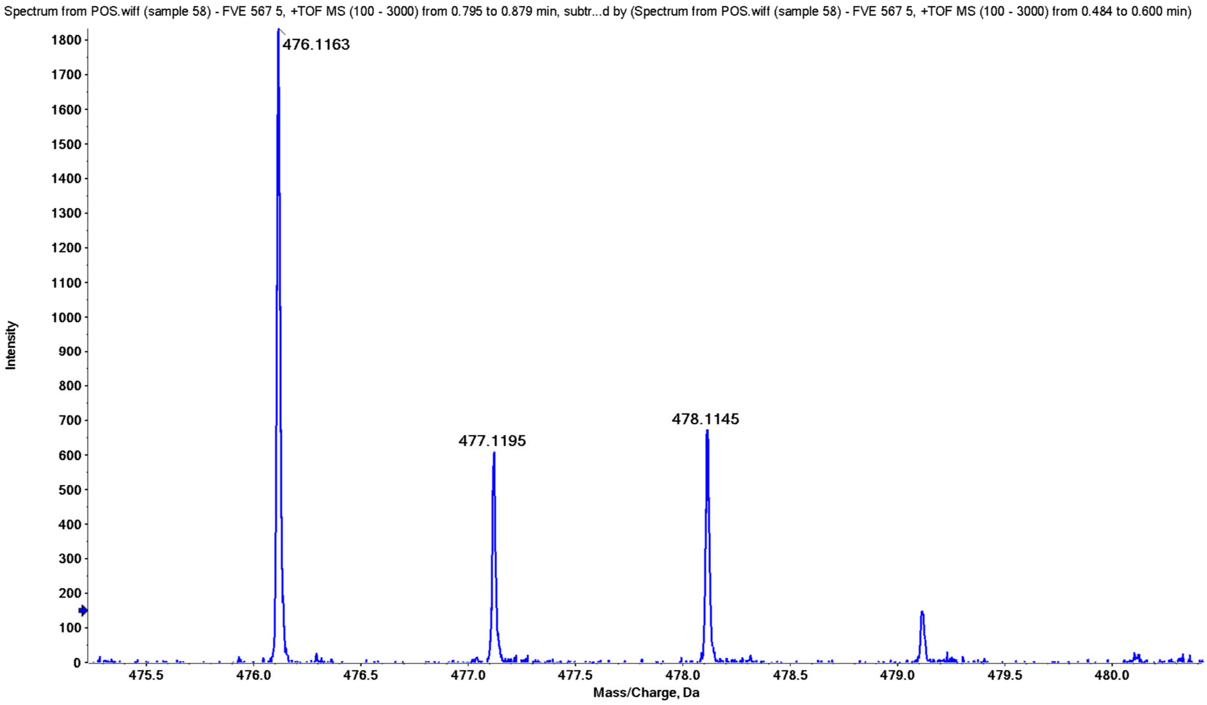

Spectrum from POS.wiff (sample 58) - FVE 567 5, +TOF MS (100 - 3000) from 0.795 to 0.879 min, subtr...d by (Spectrum from POS.wiff (sample 58) - FVE 567 5, +TOF MS (100 - 3000) from 0.484 to 0.600 min)
476.1163
477.1195
478.1145
Intensity
Mass/Charge, Da

Figure S35

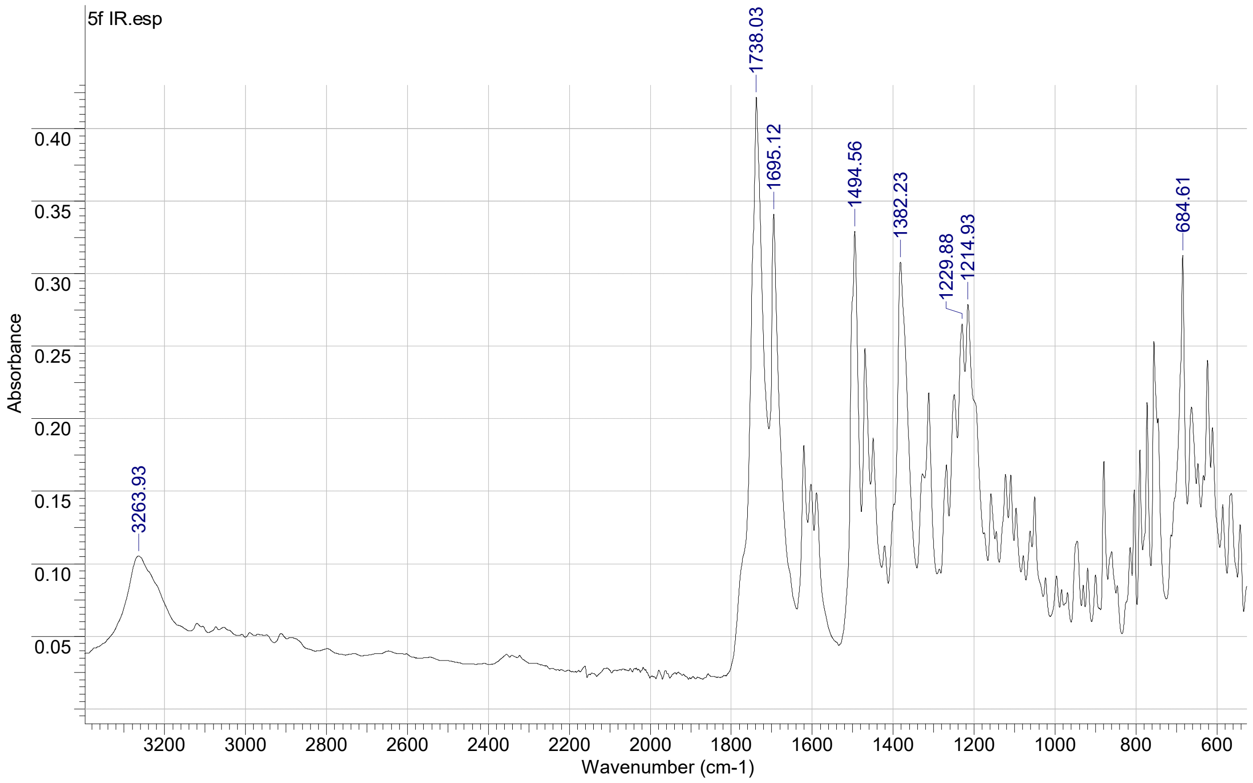

5f IR.esp
Absorbance
Wavenumber (cm-1)
3263.93
1738.03
1695.12
1494.56
1382.23
1229.88
1214.93
684.61

Figure S36

X-Ray

***5a* (CCDC-2171648)**

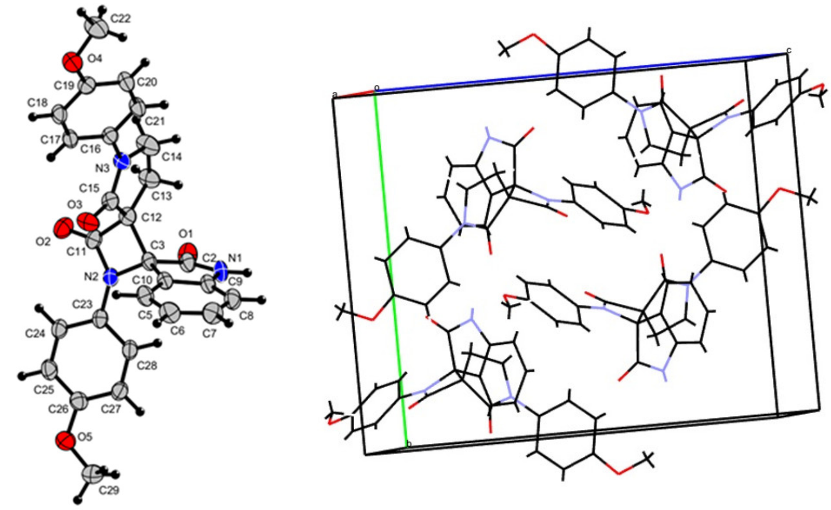

***6* (CCDC-2172682)**

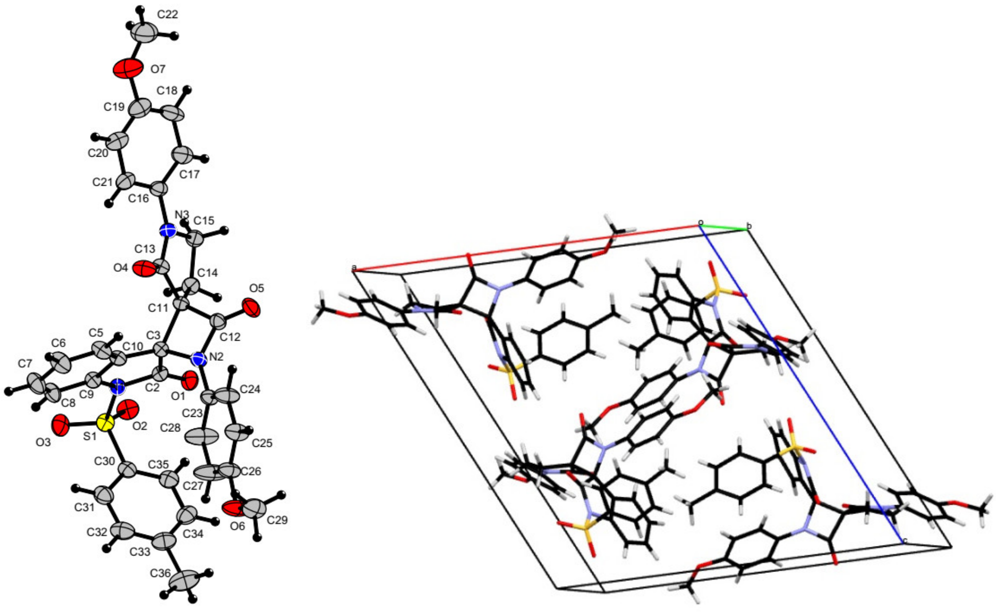

Figure S37

Table S1. Crystal data and structure refinement for compound **5a**.

Identification code	fve 4985	
Empirical formula	C27 H23 N3 O5	
Formula weight	469.48	
Temperature	295(2) K	
Wavelength	1.54186 Å	
Crystal system	Monoclinic	
Space group	P 21/n	
Unit cell dimensions	a = 10.2318(2) Å	α= 90°.
	b = 14.1437(3) Å	β= 102.589(3)°.
	c = 16.3407(4) Å	γ = 90°.
Volume	2307.90(9) $Å^3$	
Z	4	
Density (calculated)	1.351 Mg/m^3	
Absorption coefficient	0.777 mm^{-1}	
F(000)	984	
Theta range for data collection	4.178 to 68.269°.	
Index ranges	-12<=h<=8, -13<=k<=17, -16<=l<=19	
Reflections collected	17339	
Independent reflections	4170 [R(int) = 0.1830]	
Completeness to theta = 67.686°	99.4 %	
Refinement method	Full-matrix least-squares on F^2	
Data / restraints / parameters	4170 / 0 / 322	
Goodness-of-fit on F^2	0.893	
Final R indices [I>2sigma(I)]	R1 = 0.0632, wR2 = 0.1619	
R indices (all data)	R1 = 0.0925, wR2 = 0.1766	
Largest diff. peak and hole	0.308 and -0.311 e. $Å^{-3}$	

Table S2. Crystal data and structure refinement for compound 6.

Identification code	fve498_3	
Empirical formula	C34 H29 N3 O7 S	
Formula weight	623.66	
Temperature	295(2) K	
Wavelength	1.54186 Å	
Crystal system	Monoclinic	
Space group	P 21/c	
Unit cell dimensions	a = 17.7403(4) Å	α= 90°.
	b = 9.93690(10) Å	β= 116.337(2)°.
	c = 18.9331(5) Å	γ = 90°.
Volume	2991.15(12) Å^3	
Z	4	
Density (calculated)	1.385 Mg/m^3	
Absorption coefficient	1.430 mm^{-1}	
F(000)	1304	
Theta range for data collection	4.695 to 68.608°.	
Index ranges	-18<=h<=21, -11<=k<=6, -22<=l<=21	
Reflections collected	22548	
Independent reflections	5436 [R(int) = 0.0676]	
Completeness to theta = 67.686°	99.1 %	
Refinement method	Full-matrix least-squares on F^2	
Data / restraints / parameters	5436 / 0 / 410	
Goodness-of-fit on F^2	0.914	
Final R indices [I>2sigma(I)]	R1 = 0.0501, wR2 = 0.1264	
R indices (all data)	R1 = 0.0818, wR2 = 0.1376	
Extinction coefficient	0.0017(2)	
Largest diff. peak and hole	0.902 and -0.287 e. Å^{-3}	
